# Untargeted Metabolomics Sheds Light on the Diversity of Major Classes of Secondary Metabolites in the Malpighiaceae Botanical Family

**DOI:** 10.3389/fpls.2022.854842

**Published:** 2022-04-14

**Authors:** Helena Mannochio-Russo, Rafael F. de Almeida, Wilhan D. G. Nunes, Paula C. P. Bueno, Andrés M. Caraballo-Rodríguez, Anelize Bauermeister, Pieter C. Dorrestein, Vanderlan S. Bolzani

**Affiliations:** ^1^NuBBE, Department of Biochemistry and Organic Chemistry, Institute of Chemistry, São Paulo State University (UNESP), Araraquara, Brazil; ^2^Collaborative Mass Spectrometry Innovation Center, Skaggs School of Pharmacy and Pharmaceutical Sciences, University of California, San Diego, San Diego, CA, United States; ^3^Royal Botanical Gardens Kew, Science, Ecosystem Stewardship, Diversity and Livelihoods, Richmond, United Kingdom; ^4^Department of Biological Sciences, Lamol Lab, Feira de Santana State University (UEFS), Feira de Santana, Brazil; ^5^Federal Institute of Education, Science and Technology of Rondônia (IFRO), Ji-Paraná, Brazil; ^6^Max Planck Institute of Molecular Plant Physiology, Potsdam, Germany; ^7^Institute of Chemistry, Federal University of Alfenas (UNIFAL), Alfenas, Brazil

**Keywords:** chemotaxonomy, mass spectrometry, metabolite annotation, metabolomics, evolution, ancestral character reconstruction, systematics, malpighiales

## Abstract

Natural products produced by plants are one of the most investigated natural sources, which substantially contributed to the development of the natural products field. Even though these compounds are widely explored, the literature still lacks comprehensive investigations aiming to explore the evolution of secondary metabolites produced by plants, especially if classical methodologies are employed. The development of sensitive hyphenated techniques and computational tools for data processing has enabled the study of large datasets, being valuable assets for chemosystematic studies. Here, we describe a strategy for chemotaxonomic investigations using the Malpighiaceae botanical family as a model. Our workflow was based on MS/MS untargeted metabolomics, spectral searches, and recently described *in silico* classification tools, which were mapped into the latest molecular phylogeny accepted for this family. The metabolomic analysis revealed that different ionization modes and extraction protocols significantly impacted the chemical profiles, influencing the chemotaxonomic results. Spectral searches within public databases revealed several clades or genera-specific molecular families, being potential chemical markers for these taxa, while the *in silico* classification tools were able to expand the Malpighiaceae chemical space. The classes putatively annotated were used for ancestral character reconstructions, which recovered several classes of metabolites as homoplasies (i.e., non-exclusive) or synapomorphies (i.e., exclusive) for all sampled clades and genera. Our workflow combines several approaches to perform a comprehensive evolutionary chemical study. We expect it to be used on further chemotaxonomic investigations to expand chemical knowledge and reveal biological insights for compounds classes in different biological groups.

## Introduction

Plant metabolites have been widely explored since the 1930s, mainly aiming at an in-depth study of species with ethnopharmacological value, bioactive extracts and compounds, and new chemical structures (Newman and Cragg, 2020; Atanasov et al., 2021). Despite its invaluable importance, the establishment of phylogenetic diversification and distribution patterns of plant secondary metabolites is still in its early steps, and several plant families have not been deeply explored to date in this context.

The development of highly sensitive detection techniques, such as mass spectrometry (MS), allowed the investigation of plant extracts as a whole, providing a comprehensive overview of the metabolites biosynthesized by plant species (De Vos et al., 2007; Gemperline et al., 2016). In this context, chemosystematics studies employing MS, multivariate analyses, and bioinformatic tools for exploring large plant datasets have been successfully performed, raising valuable insights regarding the biosynthetic pathways involved in different phylogenetic groups (Gallon et al., 2018; Martucci et al., 2018; Ernst et al., 2019; Kang et al., 2019). It is now possible to support DNA-based phylogenetic studies at a molecular level and assist in elaborating evolutionary hypotheses based on natural products and metabolomics analyses (Schmitt and Barker, 2009). The rapid development of natural product bioinformatics tools and databases (Li and Gaquerel, 2021; Medema, 2021; Bauermeister et al., 2022) can be of great value to assist and accelerate more comprehensive chemosystematics studies aiming at taxa-specific metabolic pathways.

Even though evolutionary studies at a metabolite level are of great interest, certain factors can significantly impact the detection of metabolites and, consequently, the conclusions drawn. For instance, the extraction solvent used and the ionization mode in MS can prioritize one class of compounds over another (Floros et al., 2016; Creydt and Fischer, 2017), leading to biased results. Even though several studies compare different extraction protocols and ionization modes in metabolomics analyses, an investigation of these factors in chemosystematics studies is still poorly explored. Therefore, selecting a plant family with a broad diversity of classes of compunds identified, in addition to an extensive record of DNA-based phylogenetic studies, would be ideal for evaluating these variables, enabling a deeper chemotaxonomic investigation.

The Malpighiaceae plant family is an excellent example of both the high diversity of secondary metabolites produced by plant species, and a flowering plant family with all its genera sampled in DNA-based phylogenetic studies (Davis and Anderson, 2010; Mannochio-Russo et al., 2020). Malpighiaceae is one of the 36 families of flowering plants placed in Malpighiales by several phylogenetic studies based on chloroplast genes (Angiosperm Phylogeny Group, 1998, 2003, 2009; Angiosperm Phylogeny Group et al., 2016), being also one of the most important and diverse orders of angiosperms in tropical forests (Xi et al., 2012; Cai et al., 2019). This family currently comprises 74 genera and *ca.* 1,300 species, mostly confined to the American tropics, with Brazil being its most representative country. Few genera and species reach the tropics of Africa, Asia, and Oceania (Davis and Anderson, 2010; de Almeida et al., 2021; de Almeida and van den Berg, 2021). Some Amazonian species of Malpighiaceae are traditionally known for their psychedelic or aphrodisiac properties, with several studies focusing on the chemical characterization of these species (Samoylenko et al., 2010; Queiroz et al., 2014). On the other hand, several extra-Amazonian Malpighiaceae species are long reported as toxic to cattle (i.e., *Amorimia*, and *Niedenzuella* spp.; Riet-Correa et al., 2012; Lee et al., 2014), causing significant economic losses in the Brazilian growing cattle industry. Additionally, *Malpighia* and *Byrsonima* spp. also stand out for the nutritional value of their fruits (Belwal et al., 2018; Neri-Numa et al., 2018).

In the past two decades, traditional intrafamilial classifications of several angiosperm families (i.e., subfamily and tribe ranks), solely based on macromorphology, were proven to be non-monophyletic (i.e., artificial, and not reflecting common ancestry; Angiosperm Phylogeny Group, 1998, 2003, 2009; Angiosperm Phylogeny Group et al., 2016). During this time, Malpighiaceae has gone through unprecedented changes in its traditional classification due to several DNA-based phylogenetic studies (Cameron et al., 2001; Davis et al., 2001; Davis and Anderson, 2010; de Almeida et al., 2017, 2018). Key morphological characters of its traditional classification system (i.e., fruit types) were recovered as highly homoplastic (i.e., non-exclusive; Cameron et al., 2001; Davis et al., 2001). The inevitable recognition of unforeseen relationships within Malpighiaceae brought to light deep taxonomic problems regarding the monophyly of several genera [e.g., *Banisteriopsis* C.R.Rob., *Mascagnia* (Bertero ex DC.) Bertero, *Stigmaphyllon* A.Juss., and *Tetrapterys* Cav.], tribes (e.g., just Gaudichaudieae Horan. was recovered as monophyletic), and all its subfamilies (e.g., Byrsonimoideae W.R.Anderson and Malpighioideae A.Juss.; Cameron et al., 2001; Davis et al., 2001; Davis and Anderson, 2010; de Almeida et al., 2017). Since then, different authors have gradually proposed new genera and combinations to accommodate these newly identified lineages (Anderson, 2006, 2011; Anderson and Davis, 2006; Davis et al., 2020; de Almeida and van den Berg, 2021). Even though some morphological characters were used to reconstruct the last generic phylogeny of Malpighiaceae, no morphological characters were ever recovered, circumscribed, or discussed for its major phylogenetic clades (de Almeida and van den Berg, 2021). As a result, its traditional classification was rejected, and 10 informal clades, without any morphological circumscription, were recognized in the most recent generic phylogeny for Malpighiaceae (Davis and Anderson, 2010). More recently, phylogenomic studies were performed with six Malpighiaceae species and strongly corroborated previous phylogenetic studies within this family (Menezes et al., 2018; Ramachandran et al., 2018; Cai et al., 2019; Jo et al., 2019; Gong et al., 2020). In this context, a deeper chemical investigation of the family and mapping its chemical traits in a phylogenetic tree that reflects the evolutionary relationships among organisms can be of great value in chemotaxonomic investigations (Schmitt and Barker, 2009). It can help predict metabolically interesting groups of organisms to assist future studies of this taxon and give more support to the evolutionary hypotheses.

**Figure 1:**
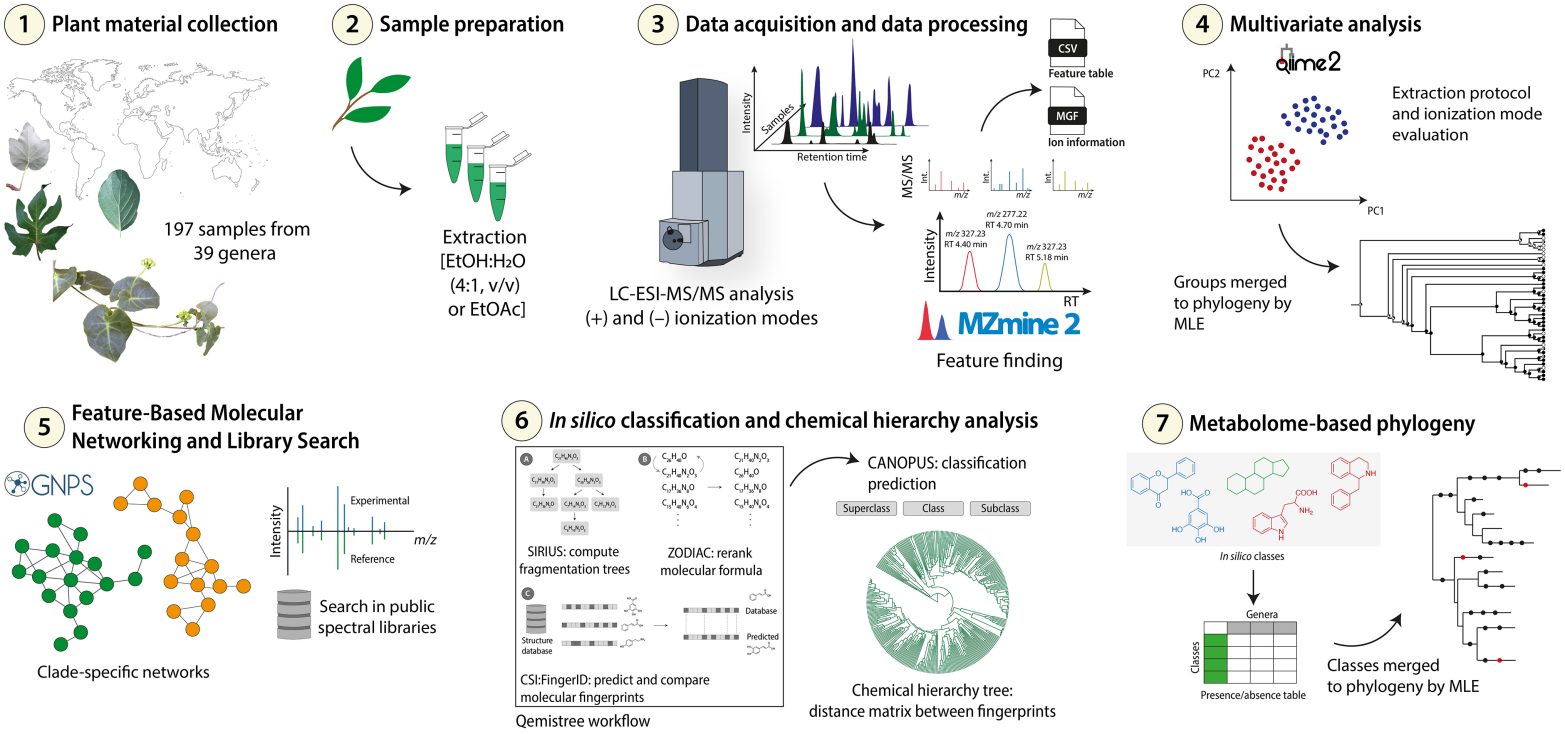
Experimental workflow followed for the metabolomics and chemosystematics analyses of Malpighiaceae samples. (1) The samples were initially collected, (2) the extracts were prepared with different solvents [EtOH:H_2_O (4:1, v/v) or EtOAc], and then (3) subjected to LC-ESI-MS/MS analysis in positive and negative ionization modes in an untargeted method. (4) The data acquired were processed for feature finding, and the exported data were used for multivariate analysis. The clustering groups observed were merged to the phylogeny using the Maximum Likelihood Estimation (MLE) for preliminary chemotaxonomic investigations. (5) The data were also used for Feature-Based Molecular Networking and library searches workflows to observe clade-specific molecular families. (6) A chemical hierarchy analysis and *in silico* classifications were obtained and finally (7) merged to the currently accepted Malpighiaceae phylogeny to determine the ubiquitous and the taxa-specific *in silico* classes.

**Figure 2:**
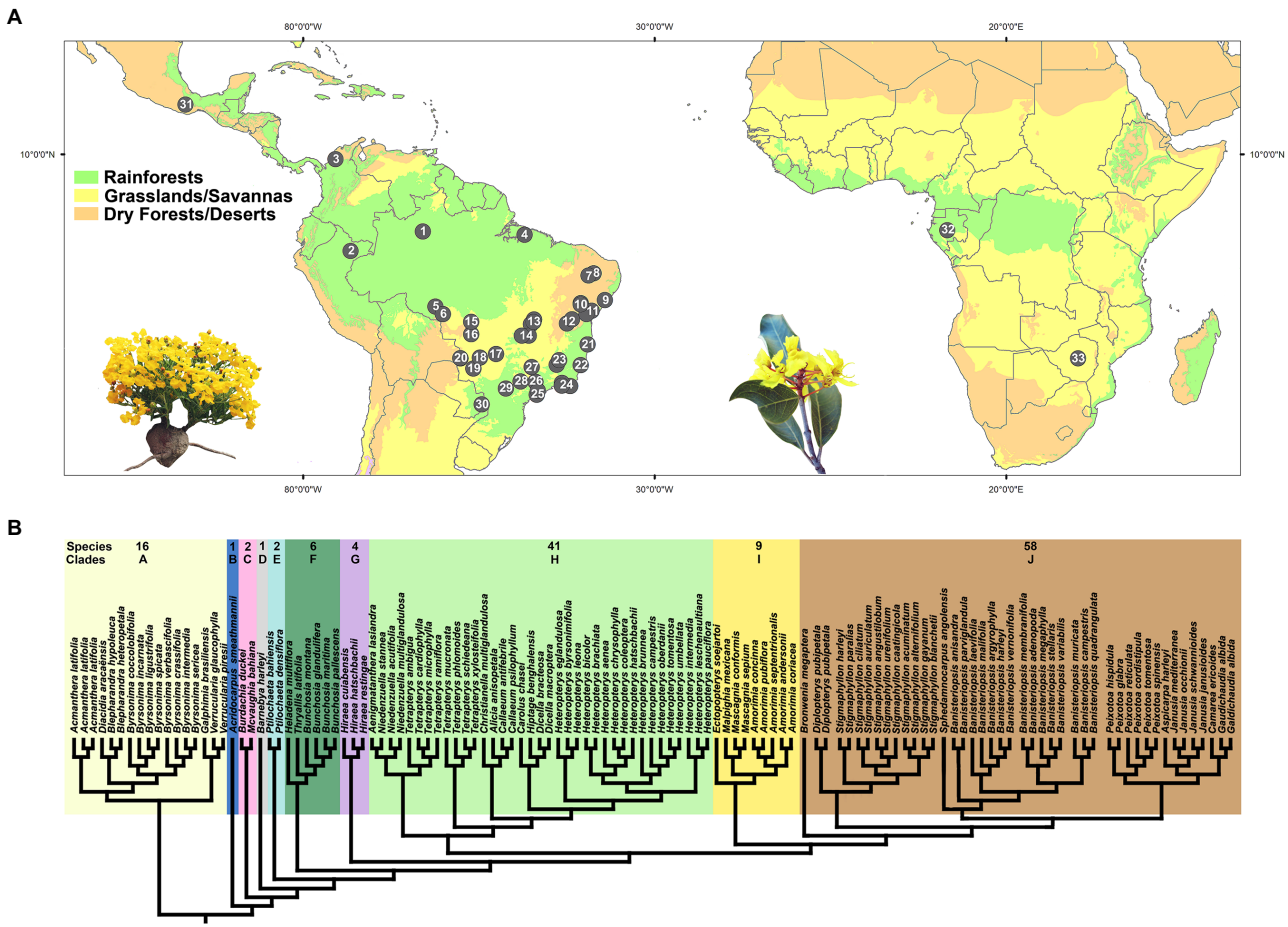
**(A)** Distribution map showing the collection sites of all samples within the American and African continents. A complete record of all collection sites (numbers on black circles) is listed in Supplementary Table 1. Photograph on the left represents a New World tropic species of Malpighiaceae (*Camarea ericoides* by R.F. Almeida). Photograph on the right represents an Old World tropic species of Malpighiaceae (*Acridocarpus excelsus* by T. Randrianarivony). **(B)** Ten major phylogenetic clades currently accepted in Malpighiaceae, based on plastid and nuclear genes, according to Davis and Anderson (2010). Major clades are shaded in different colors. Species = number of species sampled by each clade in our study. Clade A, Byrsonimoid clade; B, Acridocarpoid clade; C, Mcvaughioid clade; D, Barnebyoid clade; E, Ptilochaetoid clade; F, Bunchosioid clade; G, Hiraeoid clade; H, Tetrapteroid clade; I, Malpighioid clade; and J, Stigmaphylloid clade.

In this study, we present a new approach for chemosystematics studies, by combining natural products research with phylogenetic methods (Schmitt and Barker, 2009). We performed metabolomics analyses in combination with recently described *in silico* fragmentation predictors, chemical hierarchy analysis, and ancestral character reconstructions to map the presence/absence of the annotated metabolites in the most recent generic DNA-based phylogenetic tree of Malpighiaceae (Figure 1). For this, we evaluated a unique collection of Malpighiaceae samples, comprising 39 genera (out of 74) and 137 species from each of the major phylogenetic groups currently accepted for the family (collection distribution is shown in Figure 2). This study comprised representative samples from all the currently accepted phylogenetic clades of the family, which enabled us to obtain a comprehensive overview of the metabolites produced by this family (percentage of genera covered by each clade: 67, 50, 67, 100, 33, 60, 20, 59, 31, and 63% for clades A–J, respectively^1^). We evaluated (i) the impact of different extraction protocols and ionization modes in MS for chemotaxonomic investigations; (ii) the metabolites annotated based on spectral matches and *in silico* tools; and (iii) how the chemical diversity in Malpighiaceae evolved over the geological time in this family. With this enhanced approach, we were able to provide insights regarding the complementarity of the different ionization modes, provide the first chemical information of several Malpighiaceae genera, and draw conclusions regarding the evolution of the classes of secondary metabolites annotated in the Malpighiaceae plant family.

## Materials and Methods

### General Information

The ethyl alcohol (proof, for molecular biology) used for the extraction procedure was acquired from Sigma-Aldrich (St. Louis, United States). The ethyl acetate (HPLC grade) used for the extraction procedure was obtained by J.T. Baker (J.T. Baker-Avantor, Radnor, United States). Acetonitrile and water, both LC–MS grade, were obtained from Fisher Scientific (Fair Lawn, NJ, United States).

### Collection of Plant Material

Most plant samples were collected by R.F. Almeida on field expeditions throughout Brazil from 2013 to 2017, or were retrieved from discarded fragmented samples used for DNA extraction in molecular studies (de Almeida et al., 2017, 2018; de Almeida and van den Berg, 2020, 2021). For information regarding all sampled specimens, see Supplementary Table 1. After each collection, the plant materials were dried in a desiccator containing silica at room temperature. Samples were then frozen in liquid nitrogen and grounded in a ball mill. The samples were stored in a freezer at −20°C until the preparation of the extracts.

The authorization for conducting this study was conceded by the National System for Management of Genetic Heritage and Associated Traditional Knowledge (SISGEN), n° A6FDC2E.

### Extraction Procedure

The plant sample materials were weighed and extracted with EtOH:H_2_O (4:1, v/v) or EtOAc (100%) in a proportion of 20 mg of plant material to 1 mL of extraction solvent. The samples were homogenized in a Qiagen TissueLyzer II (Qiagen, Hilden, Germany) for 5 min at 25 MHz and extracted for additional 30 min at room temperature. The samples were centrifuged (5,000 *g*) for 15 min, and 300 μL of the supernatants were transferred to a 96-deep-well plate. The solvent was dried in a Labconco CentriVap (United States), and the plates were sealed and stored at −80°C prior to analyses.

### UHPLC-MS/MS Analysis

The extracts were initially resuspended in 200 μL MeOH:H_2_O (4:1) containing sulfachloropyridazine (2 μM) as internal standard [to monitor sample injection during the Ultra High Performance Liquid Chromatography (UHPLC)-tandem Mass Spectrometry (MS/MS) data acquisition], and sonicated for 15 min. The plates were centrifuged for 10 min at 1,300 *g*, and the supernatants were then transferred to a new 96-well plate for metabolomics analyses.

The analyses were carried out with a Thermo Scientific UltiMate 3000 UHPLC system coupled to a Maxis Impact QTOF mass spectrometer (Bruker Daltonics, Germany), controlled by the Otof Control and Hystar software packages, and equipped with ESI source. The extracts were analyzed using a Kinetex 1.7 μm C18 reversed-phase UHPLC column (50 × 2.1 mm; Phenomenex, Torrance, CA, United States), at 40°C, and an injection volume of 5 μL. The pump system consisted of water (A) and acetonitrile (B), both acidified with formic acid (0.1%, v/v), and the flow rate was set at 0.5 ml/min. The metabolites separation was achieved with 5% solvent B for 1 min, followed by a linear gradient from 5 to 100% in 5 min. The column was washed at 100% solvent B for 2 min, then returned to the initial 5% in 1 min, and the equilibration of the column was achieved for 1 min at 5% solvent B. The mass spectra were acquired in both positive and negative ionization modes, separately, in a mass range of 50–2,000 Da in data-dependent acquisition (DDA) mode. The parameters used for data acquisition were set as follows: nitrogen used as nebulizer gas with pressure at 2 bar, a capillary voltage of 4,200 V, ion source temperature of 200°C, dry gas flow at 9 L/min, and spectra rate acquisition of three spectra/s. The five most intense selected ions per spectrum were fragmented (MS/MS) using ramped collision-induced dissociation energy, ranging from 22 to 50 eV. MS/MS active exclusion was set after five spectra and released after 30 s.

The UHPLC–MS/MS data were deposited in the MassIVE Public GNPS dataset^2^ (MSV000085119) and are publicly available.

### MS/MS Data Pre-Processing

The raw data files (.d) were converted to .mzXML format using DataAnalysis software (Bruker) after lock mass correction using hexakis(1H,1H,2H-difluoroethoxy) phosphazene (Synquest Laboratories, Alachua, FL, United States), with *m/z* 622.029509. The quality of the analyses was evaluated considering the retention time and the *m/z* of a standard solution containing a mixture of six standards, which was analyzed after the completion of each row in a 96-well plate.

The .mzXML files were processed in MZmine2 (version 2.37.corr17.7_kai_merge2) for positive and negative ionization modes, separately. The parameters used for feature finding were as follows: mass detection (centroid, 1.0E3 and 1.0E1 for MS1 and MS2, respectively); chromatogram builder (minimum time span of 0.01 min, minimum height of 3.0E3, and *m/z* tolerance of 20 ppm); chromatogram deconvolution (baseline cut-off algorithm: minimum peak height: 1.0E3, peak duration range: 0.01–3 min; and baseline level: 1.0E3) with median *m/z* center calculation, *m/z* range for MS2 scan pairing of 0.02 Da and retention time (RT) range for MS2 scan pairing of 0.1 min; isotope peaks grouper (*m/z* tolerance set at 20 ppm, RT tolerance of 0.1 min, maximum charge of 3, and representative isotope set to most intense), join alignment (*m/z* tolerance of 20 ppm, weight for *m/z* and RT of 75 and 25, respectively, and RT tolerance of 0.1 min). A filter was applied in order to keep only the features with MS/MS spectra. This feature list was exported as a feature quantification table (.csv), as a MS2 spectral summary (.mgf), and with the SIRIUS export module (.mgf) for downstream analyses.

### Feature-Based Molecular Networking

To investigate the metabolic profile of the dataset, the processed LC–MS/MS data (.mgf and .csv) were used to create a Feature-Based Molecular Network (FBMN) (Nothias et al., 2020) on the GNPS platform (Wang et al., 2016) with input files containing only the features detected in the hydroethanolic extracts. The data were filtered by removing all MS/MS fragment ions within +/− 17 Da of the precursor ion in order to remove possible residual precursor ions, which can sometimes be observed in MS/MS spectra acquired in QToF equipment. Additionally, MS/MS spectra were window filtered to select only the top six fragment ions in the +/− 50 Da window throughout the spectrum. Both the precursor ion and the MS/MS fragment ion tolerance were set to 0.02 Da. A molecular network was created, in which the edges were filtered to have a cosine score above 0.7 and at least four matched peaks. Similarly, the parameters for the library search (for comparison between the experimental and library spectra) were set to have a score above 0.7 and at least four matched peaks to assist in the metabolites annotation—level three according to the metabolomics standards initiative (MSI; Sumner et al., 2007). The FBMN jobs on GNPS can be found at https://gnps.ucsd.edu/ProteoSAFe/status.jsp?task=2c5f11403ac847a298e4d7866a491143 (positive mode) and https://gnps.ucsd.edu/ProteoSAFe/status.jsp?task=501c16500476451f978311057266fbdf (negative mode).

The molecular network visualization was performed in Cytoscape (version 3.7.2, Cytoscape Consortium, San Diego, CA, United States; Shannon et al., 2003), in which the nodes correspond to ion features, while the edges between the nodes represent the MS/MS cosine scores calculated. Subnetworks in which the nodes were found in significant abundances in blanks were excluded from the Cytoscape visualization to avoid misinterpretations due to contaminants in the analyses. Sample type information was added to color the nodes as pie charts representing the relative abundance of the features across the samples (colors based on the phylogenetic clades A–J). Node size was scaled relative to the sum of the peak areas obtained in the samples in which the feature was detected. Compounds with the same MS/MS spectra, but with different retention times, were represented as separate nodes, indicating isomers.

### Chemical Hierarchy Analysis

A chemical hierarchy analysis (Qemistree; Tripathi et al., 2021) was performed with the metabolites detected in the hydroethanolic extraction protocol. For this, we used the q2-qemistree qiime2 plugin,^3^ in which the feature quantification table (.csv) and the file obtained from the SIRIUS (Dührkop et al., 2019) export module (.mgf) from MZmine were used as input. Briefly, the Qemistree workflow consists of applying SIRIUS (version 4.8.2) to the .mgf file (containing ion information), generating predicted molecular formulas for each feature. The predicted molecular formulas were reranked using ZODIAC (Ludwig et al., 2020), and the predicted molecular fingerprints were subsequently generated using fragmentation trees *via* CSI:FingerID (Dührkop et al., 2015). The chemical taxonomy of the predicted metabolite structures was obtained by CANOPUS (superclass, class, and subclass; Dührkop et al., 2021). The Euclidean pairwise distances between the molecular fingerprints were calculated, and the fingerprint vectors were hierarchically clustered to generate a tree representing the structural chemical relationships of this dataset. The tree was then pruned in order to keep only the fingerprints classified up to a superclass level. The trees were visualized interactively in EMPRESS (Cantrell et al., 2021) for data exploration, in which clade information was added as relative abundance stacked barcharts to each feature. The dendrogram obtained can be interactively visualized with the .qzv files found in the Github repository.^4^

### Statistical Analysis

The feature table exported from MZmine was used to perform unsupervised analysis using Qiime 2 (version 2020.2; Bolyen et al., 2019) bioinformatics pipeline within a Jupyter notebook. The metabolomic profiles were compared using the Bray–Curtis distance metric for comparing different extraction protocols, and using Canberra metric to investigate the four subsets individually (two extraction solvents, and two ionization modes). The Principal Coordinates Analysis (PCoA) plots showing the top three principal coordinates were visualized using EMPeror (Vázquez-Baeza et al., 2013). Permutational multivariate ANOVA (PERMANOVA; Anderson, 2001) was also performed in Qiime 2 on metabolite distance matrices to test for clustering significance (with 999 permutations), and the *F* statistic was reported as a measure of effect size.

The *in silico* classes retrieved from the Qemistree workflow (Tripathi et al., 2021) and CANOPUS (Dührkop et al., 2021) were tested for differential enrichment in the most sampled clades in study (clades A, F–J). Initially, the relative abundances were summed for the class group, the data were normalized by arc-sine square root transformation, and the effect of the clades was tested using a simple ANOVA. The adjusted values of *p* from ANOVA were obtained from the Benjamini-Hochberg method. The pairwise differences between the clades were tested through a *post-hoc* Tukey test, and the magnitude of differential enrichment was calculated through the log_2_ fold change in mean relative abundance between clades. The subclasses statistically enriched for specific clades were then selected to build a heatmap.

### Phylogenetic Analyses

In total, 39 genera (out of 74) and 139 species (out of 1,300) of Malpighiaceae were sampled, representing all of its 10 major clades recognized by recent molecular phylogenetic studies (Supplementary Table 1). Since our sampling focused on the diversity of genera and clades within the Malpighiaceae family, genera not sampled on the chemical analyses were not included in the molecular phylogenetic analysis. Sequences for the genes *matK*, *ndhF*, PHYC, and *rbcL* were retrieved from GenBank,^5^ edited using Geneious (Kearse et al., 2012), and aligned using Muscle (Edgar, 2004), with subsequent adjustments in the preliminary matrices to ensure that the nitrogenous bases were correctly aligned. The complete data matrices are available at TreeBase (https://www.treebase.org/treebase-web/search/studySearch.html, accession number S11008).

All trees were rooted in the Byrsonimoid clade (clade A), which is considered the sister-group of the other Malpighiaceae clades according to Davis and Anderson (2010). Combined analysis of plastid + nuclear regions was performed using Bayesian inference and Maximum Likelihood criteria to reconstruct our phylogenetic hypotheses. Both model-based methods were conducted with a mixed substitution model (GTR + G + I) and unlinked parameters, using MrBayes 3.1.2 (Ronquist and Huelsenbeck, 2003) and raxmlGUI2 (Edler et al., 2021). For the Bayesian inference, the Markov Chain Monte Carlo (MCMC) was run using two simultaneous independent runs with four chains each (one cold and three heated), saving one tree every 1,000 generations, for a total of 10 million generations. We excluded 20% of retained trees as “burn in”, and checked for a stationary phase of likelihood, checking for ESS values higher than 200 for all parameters on Tracer 1.6 (Rambaut et al., 2014). The posterior probabilities (PP) of clades were based on the majority rule consensus, using the stored trees, and calculated with MrBayes 3.1.2 (Ronquist and Huelsenbeck, 2003).

### Ancestral State Reconstruction

Chemical profiles were selected based on the clustering trends of the PCoA analysis for EtOH80 and EtOAc100% extracts, in both positive and negative ionization modes (four subsets). The subsets in which samples clustered into two groups (A and B) represented different chemical profiles. Character coding followed the recommendations of (Sereno, 2007) for morphological phylogenies. Primary homology hypotheses (de Pinna, 1991) were proposed for different chemical profiles, with a total of two subgroups of classes scored (i.e., presence and absence). All characters were optimized on the concatenated tree from the Bayesian inference using the maximum likelihood function with Mesquite 2.73 (Maddison and Maddison, 2006) using the mk1 algorithm.

After annotating the main classes of compounds present in the hydroethanolic extracts, we transformed the obtained quantitative matrices for the positive and negative ionization modes into qualitative matrices considering only the presence and absence of all annotated classes. Since we do not have all the analyzed species of this study sampled in the latest generic molecular phylogeny of Malpighiaceae, we summarized our results at the generic level using an arithmetic mean equation. After summarizing both matrices (retrieved from the positive and negative ionization modes), we compared all classes of secondary metabolites identified in each of them, merging their binary coding into a single column. By doing so, we obtained a total of 78 classes of secondary metabolites that were optimized into a summarized version of the phylogenetic tree presented by us in the previous step, including only the genera sampled in this study. Ancestral character reconstructions were performed using Maximum Likelihood approaches into the software Mesquite 2.73 (Maddison and Maddison, 2006) and visualized in the software Winclada (Nixon, 1999) using the fast optimization method, which favors homoplasies in the analyses.

## Results and Discussion

### Evaluation of Extraction Solvent and Ionization Mode in Malpighiaceae Chemical Diversity

The extracts (EtOH:H_2_O, 4:1 v/v; EtOAc) obtained from the 197 Malpighiaceae samples were analyzed by LC–MS/MS in both positive and negative ionization modes, and the LC–MS data were processed in MZmine2. The feature finding step resulted in a total of 24,440 and 20,150 features detected in positive and negative ionization modes, respectively. Usually, most of the dereplication tools based on LC–MS/MS consider the fragmentation of [M + H]^+^ or [M − H]^−^ adducts. Generally, molecules with pH greater than 7 (basic compounds) could be easily ionized in the positive mode making adducts with proton(s). The formation of deprotonated molecules is usually limited to compounds able to form acidic protons in the negative ionization mode. Although it is of high importance for the characterization of secondary metabolites, the signal intensity for data acquired in the negative mode is usually lower compared to the positive ionization mode. In addition, the use of positively charged ion fragmentation is more relevant due to the larger spectral library availability (Steckel and Schlosser, 2019; Wolfender et al., 2019).

**Figure 3:**
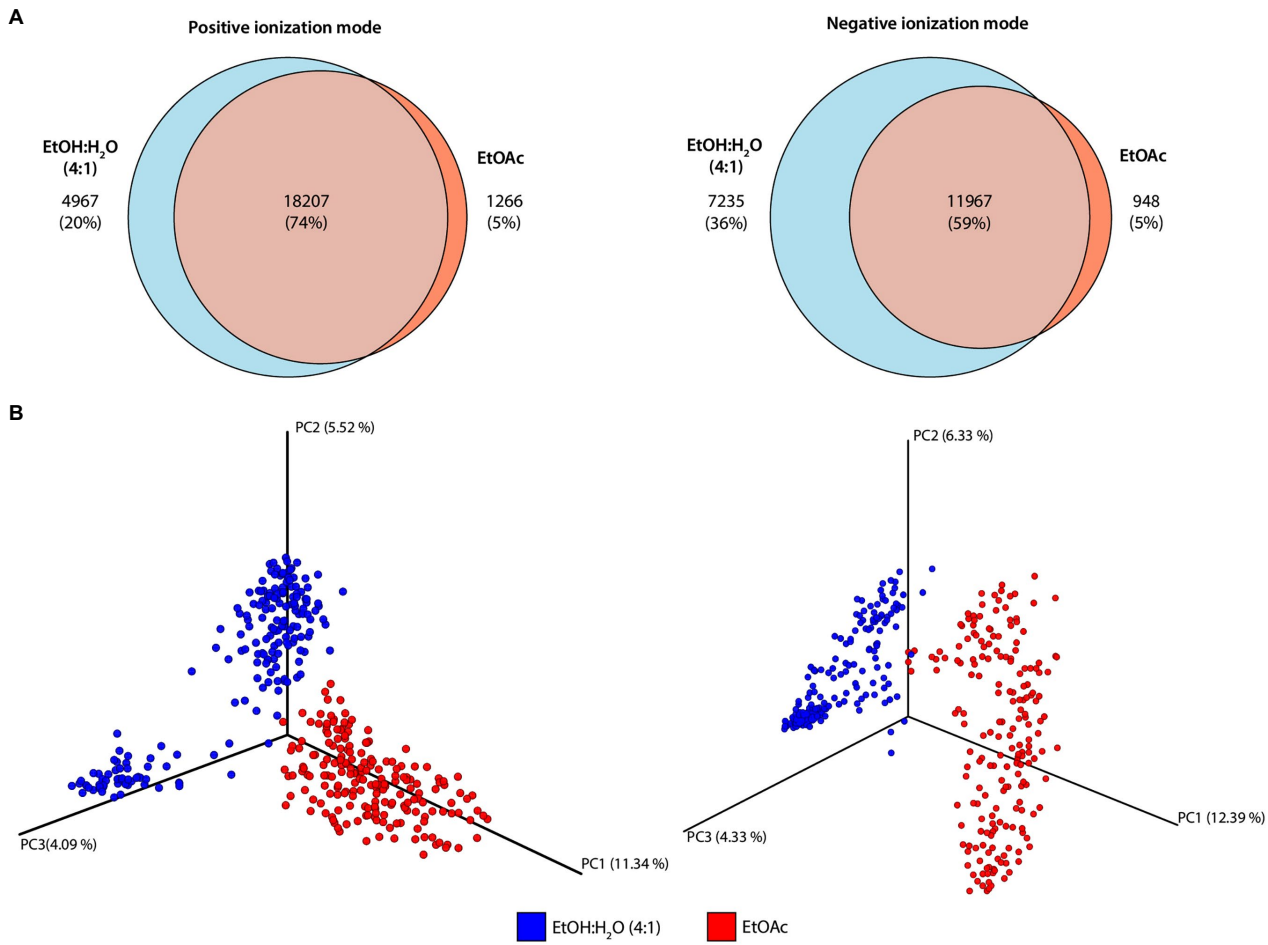
Diversity of metabolic profiles obtained in different extraction protocols and ionization modes. **(A)** Venn diagrams obtained for the different extraction protocols in positive and negative ionization modes. **(B)** Three-dimensional Principal Coordinates Analysis (PCoA) plots of the samples analyzed in different ionization modes (positive: left; negative: right) determined by Bray–Curtis distance metric. The percentage of variance explained by the principal coordinates is presented on each axis.

The Venn diagrams obtained in positive and negative ionization modes for the different extraction solvents (Figure 3A) showed that, in the positive ionization mode, 74% of the features were shared between the two extraction protocols, while in the negative ionization mode, this number dropped to 59%, with the number of metabolites exclusively detected in the hydroethanolic extraction rising from 20 to 36%. Only about 5% of the features were detected exclusively with ethyl acetate as extraction solvent, regardless of the ionization mode used. These results are in accordance with previous reports since even though there is a common core metabolome, solvent-specific metabolites are likely to be observed (Crüsemann et al., 2017).

Usually, distinct classes of metabolites are extracted with solvents of different polarities (usually hydroalcoholic mixtures, methanol, ethyl acetate, or methylene chloride), which will allow the enrichment of specific classes of metabolites (such as flavonoids, coumarins, glucosides, alkaloids, diterpenes, and saponins, among others), depending on the solvent polarity (Wolfender et al., 2019; Pilon et al., 2020). In this way, different extraction solvents can be used to obtain a broader chemical diversity. Our results show that the diversity of solvent-specific metabolites will also vary depending on the ionization mode employed.

To observe the chemical space provided by the metabolomic profiles obtained by the different extraction protocols, PCoA of the positive and negative subsets were created (Figure 3B). In summary, differently than Principal Component Analysis (PCA) which measures correlations among the samples, PCoA analysis is used to calculate distances among them, and the way these distances are calculated can result in different clustering trends in the plots. When the Euclidean distance is used in PCoA analysis, the result will be the same as if PCA was employed (Mohammadi and Prasanna, 2003; Bauermeister et al., 2022). To evaluate the impact of different extraction protocols, PCoA plots obtained by Bray–Curtis distance metric showed that the two extraction solvents resulted in very different metabolomic profiles on both ionization modes (for positive ionization mode: PERMANOVA *F* = 40.39, *p* = 0.001; for negative ionization mode: PERMANOVA *F* = 38.37, *p* = 0.001). Even though most of the features detected are shared between the two extraction protocols, the compounds’ relative abundance may significantly vary resulting in different metabolomic profiles.

In order to determine whether there would be clustering trends for each of the extracts obtained and in both ionization modes, PCoAs for these four subsets were also obtained. For the hydroethanolic extract, two main groups were formed in both ionization modes (Supplementary Figure 1 A; for positive ionization mode: PERMANOVA *F* = 28.59, *p* = 0.001; for negative ionization mode: PERMANOVA *F* = 43.92, *p* = 0.001). For the ethyl acetate extract, a separation into two groups was obtained in the positive ionization mode (PERMANOVA *F* = 18.71, *p* = 0.001; Supplementary Figure 2 A), while no group separation was observed in the negative ionization mode in any of the distance matrices tested (Supplementary Figure 1 B), reinforcing that the extraction solvent and ionization mode influence data acquisition.

The clustering trends observed for the hydroethanolic extracts were optimized using the maximum likelihood criteria into the most recent molecular phylogenetic tree of Malpighiaceae (obtained from Davis and Anderson, 2010), resulting in the cladograms shown in Supplementary Figure 1 B. These trees are graphical representations of how these different chemical profiles obtained from hydroethanolic extracts originated in specific clades (i.e., groups) through time (i.e., geological time). For the metabolites detected in the positive ionization mode, we can infer that the production of metabolites from group B originated in at least three separate geological times (firstly in part of clade A, secondly in clade C, and thirdly in the most recent common ancestor of clade E shared with all remaining clades of Malpighiaceae; i.e., clades E–J). In other words, the samples from group B are, in general, evolutionary more recent than the ones from group A. Some specific genera (such as *Amorimia*, *Alicia*, and *Banisteriopsis*), which are more recent in terms of their origins in past geological times, also cluster together with group A. A possible explanation for this tendency is that these genera are mostly found in dry environments, just like some of the early-diverging genera of Malpighiaceae (Davis et al., 2014; de Almeida et al., 2021).

On the other hand, the metabolomic profile obtained in the negative ionization mode for the hydroethanolic extracts showed a different tendency. The different chemical profiles obtained from group B in the negative ionization mode were only recovered as homoplastic to clade J (i.e., *Bronwenia*, *Diplopterys*, *Stigmaphyllon*, *Sphedamnocarpus*, and *Peixotoa*), besides also independently originated in some genera from clades A, E, F, and H.

We also evaluated the metabolomic profile obtained for the ethyl acetate extracts in both ionization modes (Supplementary Figures 2 A,C). Contrary to what we previously observed, the clustering trends observed in the positive ionization mode did not show any clear correlation with the phylogeny of the Malpighiaceae family (Supplementary Figure 1 B), while the negative ionization mode did not show any clustering trends in the metrics tested. These differences in both extraction protocols could be explained due to the different polarity ranges covered by these two solvents. From these results, we observed that different extraction protocols and ionization modes in MS can significantly impact the results from multivariate analysis and chemosystematics investigations.

### Molecular Families and Metabolite Annotation

Since the chemical profiles obtained for the hydroethanolic extracts showed a promising correlation with the evolution of Malpighiaceae, we made an in-depth investigation of the data obtained in both positive and negative ionization modes. The MS/MS library search performed in GNPS resulted in 1,070 spectral matches for the positive and 1,025 matches for the negative ionization modes, resulting in 4.6 and 5.3% of the detected chemical spaces, respectively. These matches were manually evaluated and compared with the literature, resulting in level 2 or 3 annotations according to the MSI (Sumner et al., 2007). In addition, these compounds were also searched in the *Dictionary of Natural Products*, LOTUS (Rutz et al., 2021), and Scifinder databases to inspect for previous reports in Malpighiaceae.

In addition to the library searches, the MS/MS data were visualized by molecular networking analysis. The molecular families constructed by such analysis represent the similarity of fragmentation patterns obtained by tandem mass spectrometry (MS/MS) analysis. In other words, structurally similar compounds will present similar chemical stability and functional groups, leading to similar fragmentation patterns (Yang et al., 2013). These molecular families consist of nodes (representing MS/MS spectra) and of edges connecting these nodes (representing the cosine similarity between two nodes, which measure the relatedness in MS/MS spectra; Aron et al., 2020). In this way, depending on the cosine score set in the analysis, the connections between the nodes can be more, or less strict. All the molecular families discussed in this section can be found in the Supplementary Material.

**Figure 4:**
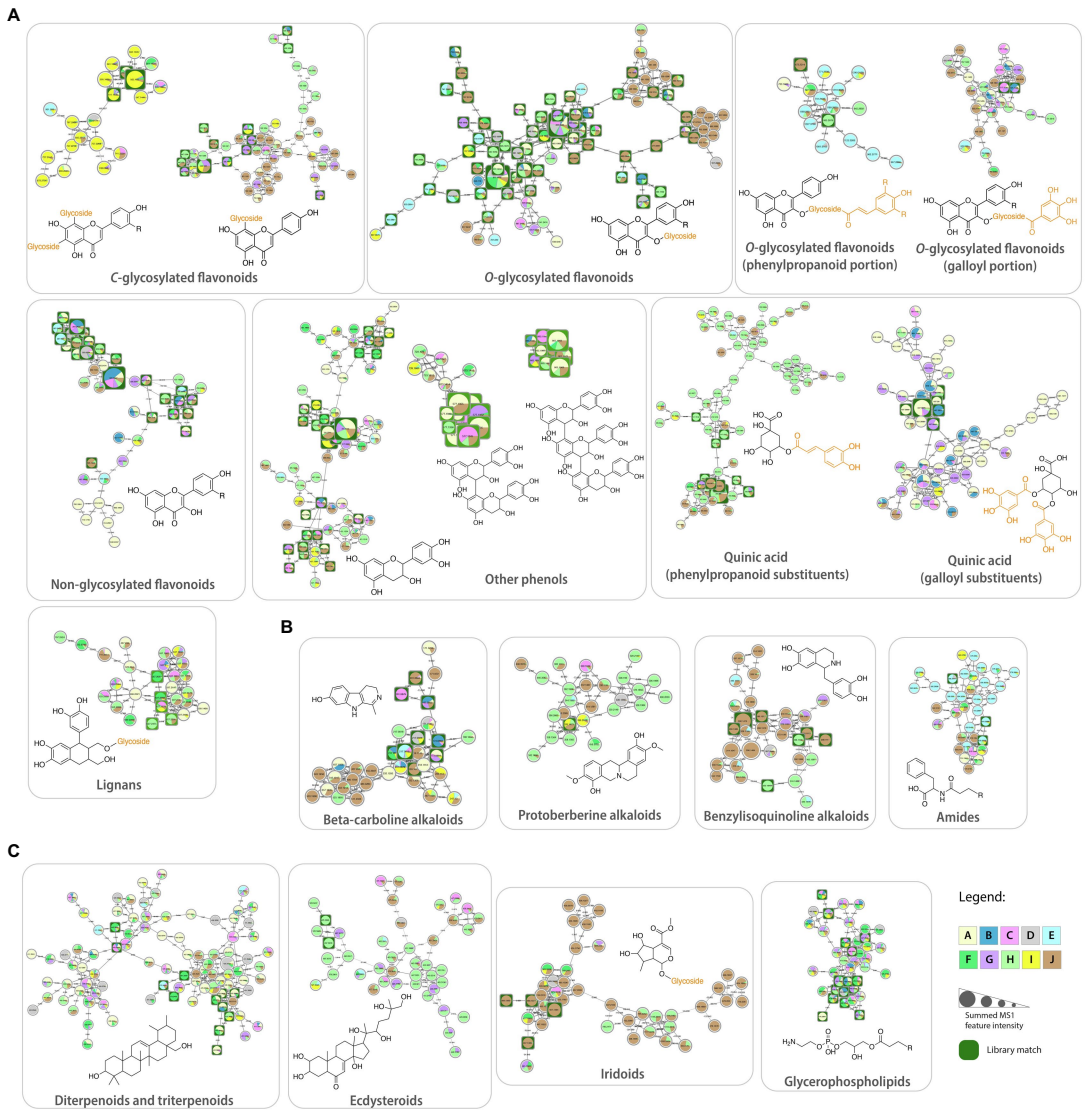
Molecular families obtained from the Feature-Based Molecular Networking workflow and annotated based on spectral matches within the GNPS platform: **(A)** phenolic compounds, **(B)** alkaloids, and **(C)** lipids and terpenoids. Each node represents a tandem mass spectrometry spectra (MS/MS), while the edges that connect them represent the MS/MS fragmentation similarity (cosine >0.7). Pie charts indicate the relative abundance of ion features in each Malpighiaceae phylogenetic clade (A–J). Node sizes are relative to the summed peak areas of the precursor ion in MS1 scans. These are level 2 or 3 annotations according to the 2007 metabolomics standards initiative (Sumner et al., 2007).

The library matches retrieved from the analyses obtained in the positive ionization mode showed the presence of a high diversity of classes of compounds, including *C*-glycosylated and *O*-glycosylated flavonoids, lipids, alkaloids, quinic acid derivatives, amides, triterpenes, iridoids, and lignans (Figure 4). Some of these chemical classes were widely detected in all phylogenetic clades, while others were more specific to particular clades or even genera.

Several molecular families related to flavonoid compounds were obtained, highlighting the difference in the fragmentation pattern in MS/MS spectra among these subclasses with different substituents. For instance, the usual MS/MS spectra obtained for *O*-glycosylated flavonoids in electrospray ionization consist of the neutral losses of the glycosidic substituents, such as hexosides (162 Da), deoxyhexosides (146 Da), and pentosides (132 Da), eventually reaching the aglycone. On the other hand, *C*-glycosylated flavonoids present a very distinct fragmentation pathway, with many more fragments observed between the precursor ion and the aglycone. The loss of the glycosidic portion is usually reached by the consecutive losses of water molecules and 120 Da, characteristic of *C*-glycosylated flavonoids (Mannochio-Russo et al., 2020). Therefore, the distinction of these two molecular families in the molecular networking analysis is expected. In addition, compounds containing two *C*-glycosidic bounds, even more fragments are expected, and we can observe two molecular families for *C*-glycosylated flavonoids.

Flavonoids with two *C*-glycosylated portions were mainly detected in samples from clade I, especially from the genera *Amorimia* and *Mascagnia* (Supplementary Figure 3). Library matches from this molecular family included apigenin-di-*C*-hexoside-pentoside and luteolin-di-*C*-hexoside, being in accordance with the previous reports for the *Amorimia* genus (Mannochio-Russo et al., 2020). On the other hand, spectral matches to flavonoids containing only one *C*-glycosylated portion (Supplementary Figure 4) were less clade-specific, being detected in samples from clades A, G, H, I, and J.

In addition to the *C*-glycosylated flavonoids, three main molecular families were observed for *O*-glycosylated ones: (1) flavonoids containing only glycosides, (2) glycosides in addition to phenylpropanoids, and (3) glycosides in addition to galloyl portions. These are also explained by the difference in general MS/MS spectra of each of these groups, in which the flavonoids containing the galloyl portion present a characteristic fragment at *m/z* 153, and the ones containing phenylpropanoids will show the fragment ions regarding this substituent (*m/z* 147 for coumaric acid, for instance). Finally, *O*-glycosylated flavonoids containing only glycosides will only present neutral losses regarding each glycosidic portion.

Several matches to *O*-glycosylated flavonoids were observed in all Malpighiaceae clades (Supplementary Figure 5), which is expected since this class of compounds is considered ubiquitous to plant species (Buer et al., 2010). This network is primarily composed of quercetin and kaempferol derivatives bound to glycosidic portions. A small cluster in this molecular family corresponds to compounds mostly present in clade J, specifically from *Stigmaphyllon* species. These compounds were annotated as flavonoids with glucuronide, acetylated, and malonylated hexoside substituents, which have not been reported in the literature for the genus *Stigmaphyllon* to date.

*O*-glycosylated flavonoids containing sugars and phenylpropanoid portions as substituents were also observed (Supplementary Figure 6), mainly present in the *Ptilochaeta* genus (clade E), which is in accordance with previous reports (Mannochio-Russo et al., 2020). In addition, sugars bound to galloyl portions (Supplementary Figure 7) were also detected as flavonoid substituents and widely found in all Malpighiaceae clades.

A network containing mainly non-glycosylated flavonoids (Supplementary Figure 8), widely distributed in all clades, was also observed, in which only the fragments relative to aglycones were detected in the MS/MS spectra. These compounds can represent, indeed, aglycones, or even in-source fragments of the *O*-glycosylated flavonoids. Additionally, a molecular family with spectral matches to catechin and afzelechin and their derivatives (Supplementary Figure 9) was also formed, containing mainly methoxylated portions and galloyl and sugar substituents.

Quinic acid derivatives were detected in higher amounts in clades H and J for phenylpropanoid substituents, and in clades A, B, and G for gallic acid substituents (Supplementary Figures 10, 11). Usually, the MS/MS patterns will contain an ion relative to the quinic acid moiety (usually with additional neutral loss of water), in addition to the fragment at *m/z* 153, relative to the galloyl portion, while other characteristic fragments will be observed for the phenylpropanoid derivatives (Mannochio-Russo et al., 2020). Previous reports described the presence of galloylquinic acids in *Byrsonima* (clade A) species (Fraige et al., 2018; Mannochio-Russo et al., 2020), while quinic acids containing phenylpropanoids substituents have been described for the genus *Heteropterys* (clade H; Huerta-Reyes et al., 2013; Paula-Freire et al., 2013).

Many lipid-like molecules presented library matches, mainly corresponding to the glycerophospholipids (Supplementary Figure 12), fatty acids, and fatty esters (Supplementary Figure 13) classes, besides jasmonic acid derivatives (Supplementary Figure 14), which were largely distributed in all Malpighiaceae clades. Glycerophospholipids also present key fragments in MS/MS analysis, such as the cleavage of the choline group, which can generate a fragment of the choline group itself (if positively charged) and the fragment of the long-chain portion (Ivanova et al., 2007). Lipids represent an important class of compounds, widely found in plants, with key roles in multiple signaling processes (Mamode Cassim et al., 2019). Along with sugars, such compounds are among the main constituents of the oil glands present in Malpighiaceae leaves and flowers (Possobom et al., 2010). Lipids are mainly produced by oil glands that play an important ecological role in Malpighiaceae, a botanical family which is mainly pollinated by oil collecting bees, and the oldest family characterized by oil-bee pollination (Anderson, 1990; Renner and Schaefer, 2010; Davis et al., 2014).

β-carboline alkaloids and other tryptophan derivatives were detected in samples from many clades (Supplementary Figure 15), including *Tetrapterys* and *Banisteriopsis* species, corroborating previous reports (Samoylenko et al., 2010; Queiroz et al., 2014). In general, β-carboline alkaloids present MS/MS spectra with fragments relative to the loss of a hydroxyl group (if present) and to the formation of a four-membered ring (*m/z* 184 and *m/z* 160, respectively, harmalol as an example). A small cluster into this molecular family showed the presence of compounds without library matches with higher *m/z* in samples from clade A and J, mainly in *Janusia*, *Banisteriopsis*, and *Byrsonima* species, which can indicate the presence of glycosylated portions due to the mass differences observed between the nodes.

Networks containing library matches corresponding to isoquinoline, protoberberine, and benzylisoquinoline alkaloids were also observed (Supplementary Figures 16–18, respectively). Isoquinoline alkaloids were mainly observed in samples from clades G, H, and J. Berberine alkaloids usually show a retro Diels-Alder (RDA) reaction and a B-ring cleavage, forming fragments *m/z* 151 and *m/z* 178, respectively, in the case of scoulerine (Qing et al., 2020). On the other hand, benzylisoquinoline alkaloids will usually present some key fragments, such as the initial loss of the nitrogen atom as ammonia or as methylamine (in the case of methylated nitrogens), in addition to an “even electron”-type McLafferty rearrangement with a reversed charge distribution, and a fragment relative to the benzyl moiety. Therefore, these key fragmentation pathways will generate ions at *m/z* 299, *m/z* 192, and *m/z* 137, taking reticuline as an example (Schmidt et al., 2005). The molecular family representing protoberberine alkaloids showed nodes corresponding to compounds mostly detected in clades H and J, particularly in *Stigmaphyllon* and *Alicia* species. Similarly, benzylisoquinoline alkaloids were also mainly detected in samples from clades H and J, especially in *Stigmaphyllon* species. These classes of compounds have not been described to date for Malpighiaceae species and represent important traits for chemosystematics studies in Malpighiaceae due to their specificity to the above-mentioned clades, especially for protoberberine and benzylisoquinoline alkaloids.

Spectral matches corresponding to amides and polyamines were also observed in molecular families (as shown in Supplementary Figures 19, 20, respectively). These compounds can generate ions relative to the *N*-cleavage of the amide bond (Barrère et al., 2014). Amides were mainly detected in the *Ptilochaeta* genus (clade E) as fatty amides and small dipeptides, while the polyamines were primarily observed in samples from clades E, H, I, and J, being this the first report of these classes of compounds for Malpighiaceae.

Several molecular families related to terpenoids were also observed, which are characterized for presenting several fragments in MS/MS spectra depending on the general skeleton of the molecule, such as RDA, McLafferty rearrangement, water losses, among others (Demarque et al., 2016). One of the largest networks obtained (Supplementary Figure 21) showed library matches to triterpenoids and their precursors. These compounds were also largely distributed among all Malpighiaceae clades, in accordance with previous reports. In fact, triterpenoids are the most described class of compounds from Malpighiaceae species to date, with numerous reports in the literature for *Acridocarpus*, *Byrsonima*, and *Galphimia* genera (Cao et al., 2004; Cardoso Taketa et al., 2004; Aguiar et al., 2005).

It is important to mention at this point that the molecular networking approach highlights the chemical similarity of compounds based on their fragmentation patterns, which may not necessarily reflect their biosynthetic origin. For instance, some diterpenoids are grouped in the same molecular family as the triterpenoids, even though their biosynthesis differs from the plastidial MEP and the cytosolic mevalonate pathway. The connection between them can be observed since these two classes of compounds present similar MS/MS fragmentation patterns. A possible way to separate these compounds into two distinct molecular families would be to significantly increase the threshold for the cosine score similarity (set to 0.7 in this work). However, in the molecular networking analysis, the cosine value is set for the entire dataset, and thus, we used an intermediary value that was adequate for most of the families discussed in this work. Since different chemical classes present different key MS/MS fragments, it is very likely that while separating triterpenoids from diterpenoids, other molecular families can be fragmented into smaller networks, even though they can present significant correlations. In addition, it is important to emphasize that even though we can increase this cosine threshold, it is very likely that the diterpenoids and triterpenoids will not be perfectly separated since these two classes share several key MS/MS fragments.

A separate molecular family relative to ecdysteroids, another class of terpenoids, was also observed (Supplementary Figure 22), and mainly detected in samples from clade H, particularly in *Niedenzuella* species. These compounds are mainly characterized by the presence of consecutive neutral losses of water molecules, in addition to the hydrocarbon side chain cleavage (Lavrynenko et al., 2013; Mannochio-Russo et al., 2020). The presence of ecdysteroids in *Niedenzuella multiglandulosa* was recently described in the literature (Mannochio Russo et al., 2020; Mannochio-Russo et al., 2020), being the only reports of this class of compounds in the Malpighiaceae family. Our results showed a wide diversity of ecdysteroids in all six *Niedenzuella* species sampled, being possible chemical markers for the *Niedenzuella* genus. This represents about 40% of all the *Niedenzuella* species reported to date, and future studies with other *Niedenzuella* species should be performed to corroborate this hypothesis. Moreover, these steroids were also detected in *Hiraea*, *Tetrapterys* (including in the recently segregated *Glicophyllum* species, evolutionary close to *Niedenzuella*), and *Peixotoa* genera (clades G, H, and J, respectively).

Iridoids, another relevant class of terpenoids, were also annotated based on library matches, which are shown in Supplementary Figure 23. These compounds present key fragments relative to the neutral losses of the glycosidic portions, in addition to water losses and other possible substituents (Wu et al., 2010). The correspondent networks revealed that these iridoids are mainly present in samples from clade J, especially in *Stigmaphyllon* species, being in accordance with previous reports (Sainty et al., 1981; Davioud et al., 1985). To date, iridoids have only been reported for *Stigmaphyllon* genus in Malpighiaceae, and in this study, we observe that these compounds are also present in samples from other clades, in particular, *Heteropterys oberdanii* species (clade H). Furthermore, a molecular family with a spectral match to a secoiridoid (Supplementary Figure 24) was also observed, with part of the compounds widely distributed among all the clades, and part of them mainly detected in *Stigmaphyllon* species. Finally, molecular families relative to neolignans and furofuranoid lignans (Supplementary Figure 25) were observed and widely distributed in Malpighiaceae clades, which have not been previously reported in this family to date.

The library matches obtained for the negative ionization mode and the molecular networks formed were also inspected, and these analyses showed mainly the same classes of compounds described for the positive ionization mode. Two molecular families stood out for presenting library matches to classes of compounds that were not observed in the positive ionization mode: proanthocyanidins and lignans (Supplementary Figures 26, 27). Proanthocyanidins dimers and trimers were observed in all Malpighiaceae clades, with characteristic fragmentation patterns (such as RDA), corroborating previous reports (Fraige et al., 2018; Mannochio-Russo et al., 2020). Lignans have already been reported for *Tetrapterys mucronata* (Queiroz et al., 2014), and here we observe that, in addition to *T. mucronata* species (clade H), these compounds are also present in other clades.

It is important to emphasize that the compounds discussed above represent only a part of the compounds detected in this study. In fact, about 87% of the molecular families (composed of two nodes or more) obtained in both ionization modes did not show any spectral match, including networks composed mainly or exclusively by nodes representing specific clades/genera. These numbers point to the possibility of undescribed natural products. On the other hand, even considering only the spectral matches, it was possible to obtain important information regarding the classes of compounds produced by these plant species. Another crucial point to be considered is that the sampling in this study comprises a larger number of samples from clades A, H, I, and J, and that these clades presented most of the spectral matches observed. It is possible that if a greater number of samples from other clades are included, more spectral matches can be retrieved to give more insights on other clades as well.

### *In silico* Metabolite Annotation and Chemical Hierarchy Analysis

**Figure 5:**
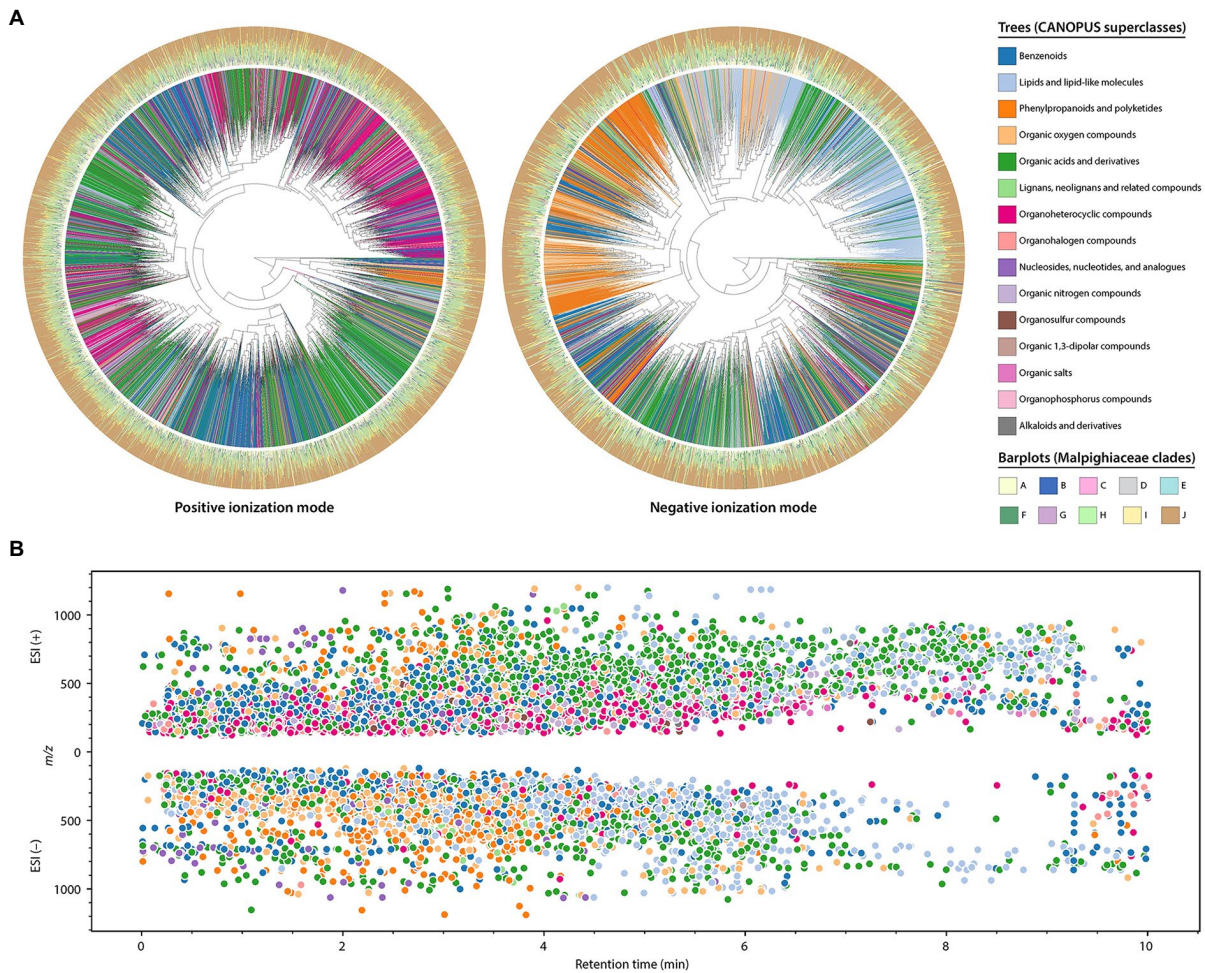
*In silico* annotations obtained for the Malpighiaceae dataset from the Qemistree workflow combined with the CANOPUS classification tool. These are level 3 annotations according to the 2007 metabolomics standards initiative (Sumner et al., 2007). **(A)** Chemical hierarchies of the predicted molecular fingerprints from the Malpighiaceae plant samples analyzed in positive (left) and negative (right) ionization modes. The trees are pruned to keep fingerprints which were classified up to a superclass level in CANOPUS. The branch colors indicate the superclasses, while the barplots of the outer ring indicate the relative abundance of a molecular fingerprint in each Malpighiaceae clade. **(B)** The ion features classified *in silico* are mapped based on the CANOPUS superclass (same colormap described in **A**). The *x* and *y* axes indicate the retention time and *m/z* value, respectively.

In order to amplify the chemical space from the Malpighiaceae dataset and have additional information about the classes of compounds detected, we used the Qemistree workflow combined with the CANOPUS classification tool for systematic compound class annotation (Dührkop et al., 2021; Tripathi et al., 2021). These *in silico* classifications consist of level 3 annotations according to the MSI (Sumner et al., 2007). In this way, it was possible to construct a chemical tree based on molecular fingerprints from MS/MS spectra and *in silico* classification tools. A total of 7,489 and 3,773 fingerprints were generated and classified at a superclass level for the positive and negative ionization modes, respectively. In this way, two chemical hierarchy trees were obtained, as shown in Figure 5A.

From the results obtained, it is evident that the ionization mode employed greatly influences the classifications obtained. At a superclass level, the ones most retrieved in the positive ionization modes were the “organic acids and derivatives,” followed by “benzenoids” and “organoheterocyclic compounds.” In contrast, most of the superclasses retrieved in the negative ionization mode consisted of “lipids and lipid-like molecules,” “organic acids and derivatives,” and “benzenoids” (Supplementary Figure 28 A). At a CANOPUS class level, the “carboxylic acids and derivatives” was the main recovered class in both ionization modes. Ion features classified as “benzene and substituted derivatives,” and “azoles” were also observed several times for the positive ionization mode, while for the negative ionization mode, “organooxygen compounds” and “fatty acyls” were the second and third most abundant classifications.

These results confirm some of the conclusions drawn from the molecular networks, such as the presence of lipids and lipid-like molecules in several Malpighiaceae clades. The molecular networks and library searches in spectral libraries, combined with the *in silico* approaches based on structural databases allowed us to expand the Malpighiaceae chemical space. Several hypotheses raised from the molecular networks were corroborated with the *in silico* classifications, giving higher confidence in these results.

The classifications obtained in the different ionization modes are also shown distributed in the chromatographic run (Figure 5B). In addition to being possible to observe differences in classifications between ionization modes, the retention time ranges also vary for specific superclasses. For instance, in the negative ionization mode, organic oxygen compounds, and phenylpropanoids and polyketides elute from near the dead volume to approximately 4 min, while the ion features classified as lipids and lipid-like molecules present higher retention times.

The putative chemical classes retrieved as highly correlated with the most sampled clades (clades A, F, G, H, I, and J; ANOVA *p* < 0.05) were selected to build a heatmap (Supplementary Figure 29). The normalized distribution pattern of the different classes within the sampled genera showed that specific classes are significantly enriched in determined genera/clades, which corroborates our observations retrieved from the molecular networks. In addition, once again, the results obtained for the positive and negative ionization modes differ.

These results show that both ionization modes result in complementary chemical classifications, which is crucial for comprehensive chemotaxonomic investigations. In fact, the *in silico* tools used in this study relies on public spectral and structural databases, which are known to be more populated with data on the positive ionization mode (Tripathi et al., 2021). In this way, the results obtained for the negative ionization mode are less extensive compared to the positive ionization mode.

### Ancestral Character Reconstructions for the Classes of Secondary Metabolites Annotated in Malpighiaceae

Ancestral character reconstruction analyzes have been increasingly encouraged in natural products studies since the early 2010s (Schmitt and Barker, 2009). It has been used in the chemistry of natural products to investigate chemical evolutionary relationships comprising different organisms, such as plants, fungi, and animals (Lumbsch et al., 2006; Bondoc et al., 2013; Allevato et al., 2019; Coley et al., 2019; Chen et al., 2020; Beaulieu et al., 2021). Phylogenetic methods have proven to be a promising approach to explore the evolution of chemical compounds in a specific genus or family of plants and other living organisms. This analysis consists of optimizing binary character states (presence/absence) into a DNA-based molecular phylogenetic tree by statistically testing using Maximum Likelihood Estimation, and recovering which character states (such as chemical classes) characterizes a given clade or taxonomic group within the molecular phylogenetic tree (i.e., genera or major clades recovered by DNA-based phylogenetic studies in Malpighiaceae). In this way, we can determine the statistical probability of the ancestor of a particular genus or clade to exclusively (i.e., synapomorphy) or non-exclusively (i.e., homoplasy) show the presence or absence of a specific chemical class. These exclusive or non-exclusive chemical classes can be used in future studies to circumscribe the analyzed taxa into a new classification, which additionally considers chemical information.

**Figure 6:**
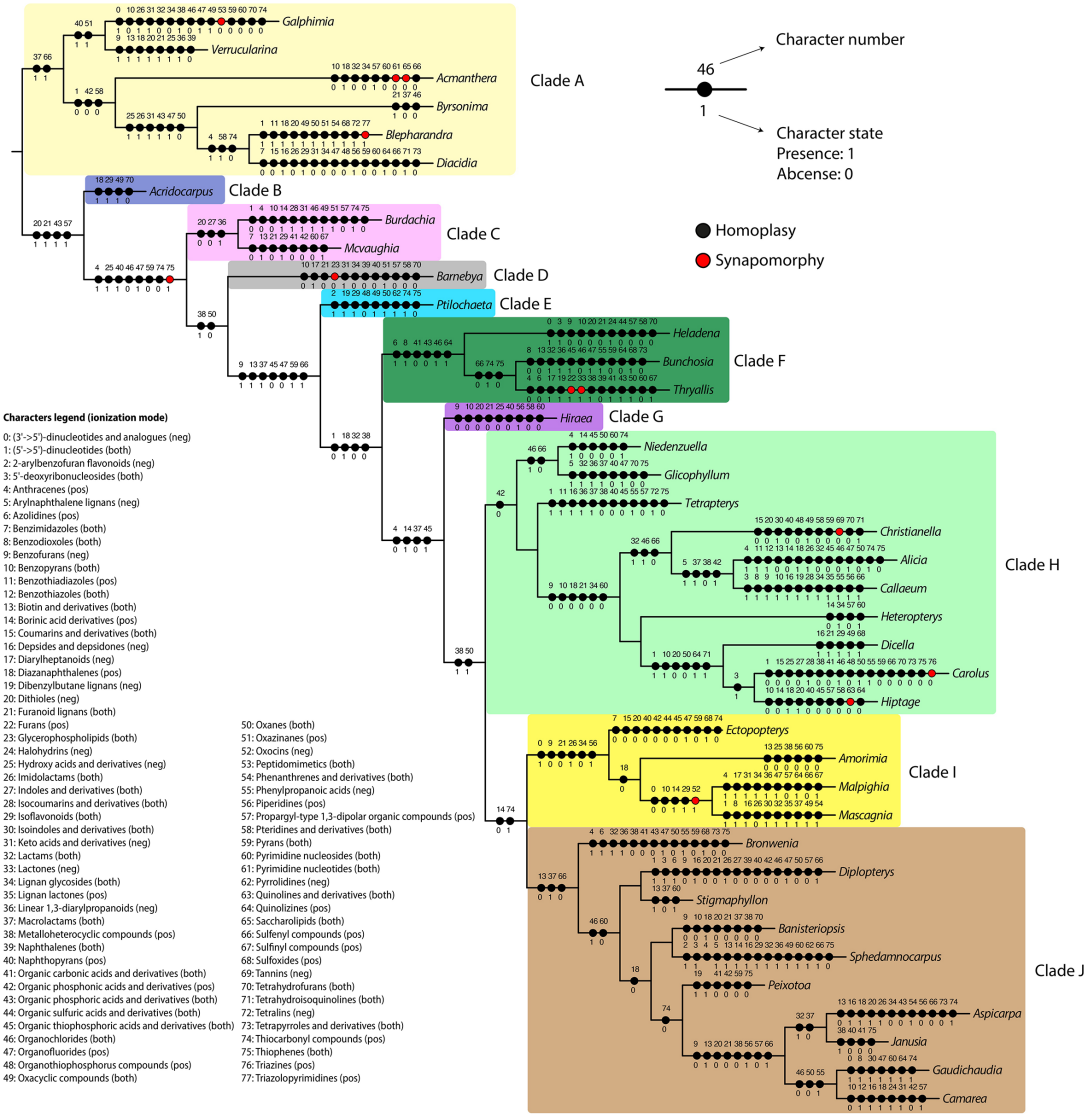
Summary of the maximum likelihood ancestral state reconstruction for the *in silico* classifications obtained at a class level. Each chemical class was treated as a character (0–77), and character states were binary-coded for each genus (1: present; 0: absent). Black and red circles represent homoplasies and synapomorphies, respectively. Clades highlighted represent the Malpighiaceae major clades recognized by recent molecular phylogenetic studies according to Davis and Anderson (2010).

In the present study, the *in silico* classifications obtained were used to map the evolutionary history of the classes retrieved in our analyses using the maximum likelihood criteria in the Malpighiaceae DNA-based molecular phylogeny (Figure 6). The classifications obtained in the positive and negative ionization modes were combined to provide a general overview of the total number of chemical classes obtained for the family. Classes retrieved as homoplasies or synapomorphies for the major Malpighiaceae clades (A–J) are described in Table 1, while the ones retrieved for all Malpighiaceae clades and genera are extensively presented in Supplementary Table 2. From the 113 *in silico* classes retrieved, 35 were present in all genera, such as fatty acyls, flavonoids, glycerolipids, phenols, and purine nucleosides, compounds widely distributed in plants, with a variety of ecological roles in these organisms. Nonetheless, future additional studies sampling the remaining botanical families included in the order Malpighiales are needed to properly evaluate the relevance of these 35 classes of metabolites for the Malpighiaceae family as a whole.

**Table 1:** Characters retrieved from the ancestral characters reconstruction (clades) based on the classifications obtained *in silico* for Malpighiaceae samples.

	Classes present^†^	Classes absent
Clade A (Byrsonimoid clade)	Macrolactams (both); Sulfenyl compounds (pos)	–
Clade B (Acridocarpoid clade)	Diazanaphthalenes (pos); Isoflavonoids (both); Oxacyclic compounds (both)	Tetrahydrofurans (both)
Clade C (Mcvaughioid clade)	Linear 1,3-diarylpropanoids (neg)	Dithioles (neg); Indoles and derivatives (both)
Clade D (Barnebyoid clade)	Diarylheptanoids (neg); Keto acids and derivatives (neg); Oxazinanes (pos)	Benzopyrans (both); Furanoid lignans (both); Glycerophospholipids (both)^§^, Lignan glycosides (both); Naphthalenes (both); Naphthopyrans (pos); Propargyl-type 1,3-dipolar organic compounds (pos); Pteridines and derivatives (both); Tetrahydrofurans (both)
Clade E (Ptilochaetoid clade)	2-arylbenzofuran flavonoids (neg); Dibenzylbutane lignans (neg); Isoflavonoids (both); Oxacyclic compounds (both); Oxanes (both); Pyrrolidines (neg); Thiocarbonyl compounds (pos)	Organothiophosphorus compounds (pos); Thiophenes (both)
Clade F (Bunchosioid clade)	Azolidines (pos); Benzodioxoles (both); Organochlorides (both); Quinolizines (pos)	Organic carbonic acids and derivatives (both); Organic phosphoric acids and derivatives (both)
Clade G (Hiraeoid clade)	Piperidines (pos)	Benzofurans (neg); Benzopyrans (both); Dithioles (neg); Furanoid lignans (both); Hydroxy acids and derivatives (neg); Naphthopyrans (pos); Pteridines and derivatives (both); Pyrimidine nucleosides (both)
Clade H (Tetrapteroid clade)	–	Organic phosphonic acids and derivatives (pos)
Clade I (Malpighioid clade)	(3′➔5′)-dinucleotides and analogues (neg); Imidolactams (both); Piperidines (pos)	Benzofurans (neg); Furanoid lignans (both); Lignan glycosides (both)
Clade J (Stigmaphylloid clade)	Macrolactams (both)	Biotin and derivatives (both); Sulfenyl compounds (pos)

The ionization mode in which each classification was obtained is described (pos, positive ionization mode; neg, negative ionization mode; and both, both ionization modes).

† Classes retrieved as present in all Malpighiaceae clades: Alkyl halides (both); Allyl-type 1,3-dipolar organic compounds (both); Aryl halides (both); Azacyclic compounds (both); Azoles (both); Benzene and substituted derivatives (both); Boronic acid derivatives (both); Carboxylic acids and derivatives (both); Cinnamic acids and derivatives (both); Diazinanes (pos); Diazines (both); Fatty Acyls (both); Flavonoids (both); Glycerolipids (both); Heteroaromatic compounds (both); Imidazopyrimidines (both); Macrolides and analogues (pos); Organic metal salts (pos); Organic sulfonic acids and derivatives (both); Organonitrogen compounds (both); Organooxygen compounds (both); Phenol ethers (both); Phenols (both); Prenol lipids (both); Purine nucleosides (both); Purine nucleotides (both); Pyridines and derivatives (both); Sphingolipids (both); Steroids and steroid derivatives (both); Sulfonyls (pos); and Thioethers (both).

§ Synapomorphy.

Regarding the 10 major clades of Malpighiaceae, all of them were recovered with at least one homoplastic or synapomorphic class of metabolites supporting them (Table 1; Supplementary Table 2). The 10 major clades in Malpighiaceae were characterized by the presence and absence of 22 and 23 classes of metabolites (i.e., homoplasies), respectively, with the absence of glycerophospholipids being recovered as a synapomorphy of Clade D. It is worth mentioning that even though most classes of secondary metabolites circumscribing all the 10 major clades of Malpighiaceae were recovered as homoplasies, their presence or absence was recorded exclusively for each clade, with reversions (i.e., parallelisms) only being recorded within a few distantly related subclades or genera. The results retrieved from this analysis corroborate some of the conclusions obtained from the molecular networks and from the *in silico* classifications. For instance, glycerophospholipids, fatty acids, fatty esters, furofuranoid lignans, and prenol lipids classes were widely distributed in our molecular networking analyses for all phylogenetic clades, and these classes were also extensively recovered in the ancestral character reconstructions (i.e., appeared in different geological times). In addition, it also clearly shows which classes were important to circumscribe specific clades/genera.

Our chemotaxonomic approach based on MS/MS analyses of Malpighiaceae plant samples allowed us to obtain a comprehensive overview of the classes of secondary metabolites produced by this taxon. Plant secondary metabolites are known to show patterns of occurrence in certain taxa (Wink, 2003). However, it is important to emphasize that the chemical diversity of a sample is highly influenced by many factors, such as genetic variation and environmental influences (soil nutrients, humidity, herbivory, and ecological interactions, among others; Isah, 2019). Studies have shown that even the same species collected in different biomes (Bueno et al., 2021) or different seasons (Zanatta et al., 2021) can produce different relative amounts of specific metabolites. Therefore, even for a single species, a range of factors can be explored to understand how the metabolites are affected to infer their possible ecological roles. However, it is important to emphasize that the ancestral character reconstructions obtained only focus on qualitative characters (presence and absence) rather than quantitative ones.

Our study provides a starting point for follow-up and systematic evaluation of such factors, and in-depth studies must be conducted to confirm and expand these chemotaxonomic conclusions for both Malpighiaceae and Malpighiales. In fact, there is a big jump from a large (but limited) sampling, as the one we present here, toward a chemotaxonomic investigation of the entire plant family. Nonetheless, as shown by several recent studies mentioned above, using phylogenetic methods with chemistry of natural products data is a promising and revolutionary new line of research that aims to elucidate the evolution of specialized metabolites in living organisms. Therefore, future investigations must be conducted to confirm the hypotheses raised in our study, especially for species that do not present any previous phytochemical study. In addition, our efforts were directed to obtain chemical information at a major clades and generic levels. There might be discrepancies if the ancestral character state reconstructions are obtained at different taxonomic levels, such as intrageneric and species levels.

Finally, the relevance of our evolutionary approach to the study of secondary metabolites can be evidenced if we consider as examples three relevant classes of plant secondary metabolites (Table 1: furanoid lignans, isoflavonoids, and piperidines). The absence of the phenylpropanoids furanoid lignans in clades D, G, and I is regarded as an informative homoplasy in our analyzes since this information can be used to point which clades in the family one must focus on in future studies to search for this class of metabolites. This information can also be used in chemosystematic studies of Malpighiaceae to chemically characterize these clades and help establish a new classification system based on chemical compounds and morphology, following de Almeida et al. (2017). On the other hand, the presence of the phenolic isoflavonoids in clades B and E, and the alkaloid class of piperidines in clades G and I are also informative homoplasies that can be interpreted in the same light as the furanoid lignans. In addition, it is important to emphasize that the *in silico* classifications obtained rely on structural databases populated with compounds from diverse sources, including plants and microorganisms metabolites. Therefore, some *in silico* classifications of metabolites more usually found in microorganisms may occur in plant datasets (for instance, the features classified as lactams). A deeper investigation in each taxonomic group should be performed in the future to confirm their presence. In fact, the population of such databases with more compounds derived from plants is necessary to have more accurate information regarding plant species in the future.

The chemical characterization of all Malpighiaceae clades is the first step toward enabling a new research line on the evolution of secondary metabolites in this plant family since this family already has a dated and calibrated molecular phylogeny available in the literature (Wink, 2003; Davis et al., 2014). Merging both analyses would allow us to infer the geological time in which all classes of secondary metabolites have arisen in Malpighiaceae and correlate these dates with past biogeographic events, such as the colonization of different biomes by the most recent common ancestor of all analyzed clades (de Almeida et al., 2018). Additionally, it would also be possible to evaluate which Malpighiaceae lineages experienced a higher diversification throughout the geological time and identify, and which classes of secondary metabolites are correlated with these diversification events (Xi et al., 2012).

## Conclusion

Metabolomics analyses based on tandem mass spectrometry and bioinformatics tools have enabled a more comprehensive investigation of the metabolites produced by organisms, and have been increasingly used for this purpose. In fact, due to the low amount of material necessary, it is possible to investigate entire families for chemoevolutionary studies based on the remaining samples from molecular phylogenies used for total DNA extraction. Even though there was a significant advance in this field, many caveats must be considered for proper use. The ionization mode and extraction protocols must be carefully evaluated since these factors influence the results, especially when aiming at chemotaxonomic investigations. Our results showed that positive and negative ionization modes lead to complementary results both in library searches and *in silico* classification tools. However, as the public libraries are more populated with data acquired in the positive ionization mode, less extensive information can be retrieved from analyses performed in the negative ionization mode. More complete and precise results will certainly be obtained for chemotaxonomic studies as these databases get more populated and new bioinformatic tools are developed. Similarly, conclusions obtained from *in silico* approaches must be confirmed with complementary techniques, and the classical methodologies are of great value for deeper investigations. In addition, the population of structural databases with more plant-derived compounds will be of great value to have more accurate results for these organisms.

Our study explored several Malpighiaceae plant species, genera, and clades for the first time, which greatly improved the chemical knowledge of this family. There are several challenges in performing chemotaxonomic investigations at a plant family level, and the evolutionary conclusions retrieved must be carefully inspected; however, they can be of great value to underpin interesting features in the chemodiversity of a certain taxon. We hope that our findings guide future studies in Malpighiaceae as we reported evidence of specific classes of compounds that most likely occur in specific clades or genera. Therefore, if a particular chemical class is of interest for presenting specific biological activities, one can focus their search on specific groups pointed out in this study. All the software and libraries used in this study are publicly available, making this workflow accessible to be reproduced in other taxa. In addition, we expect that the workflow followed in this study will be used in future studies in several fields, such as chemotaxonomy, metabolomics, chemical ecology, and for the discovery of new natural products.

## Data Availability Statement

The mass spectrometry data can be accessed on the Mass spectrometry Interactive Virtual Environment (MassIVE) as the dataset MSV000085119, which is publicly available. The Feature-Based Molecular Networking jobs on GNPS can be accessed online at: https://gnps.ucsd.edu/ProteoSAFe/status.jsp?task=2c5f11403ac847a298e4d7866a491143 and https://gnps.ucsd.edu/ProteoSAFe/status.jsp?task=501c16500476451f978311057266fbdf for positive and negative ionization modes, respectively.

The Cytoscape visualization files, the feature tables (.csv) and ion information (.mgf) files exported from MZmine2, and the files generated in the Qemistree workflow in Qiime2 are also available in MSV000085119. The chemical hierarchy tree for interactive visualization and scripts used in this project are available in https://github.com/helenamrusso/Malpighiaceae_supplementary.

## Author Contributions

HM-R designed the study, performed the extraction and LC–MS/MS analyses, processed and analyzed the MS/MS data, performed the ancestral character reconstructions, inspected the results, wrote the manuscript, and revised the manuscript. RA designed the study, collected and/or identified the species, performed the ancestral character reconstructions, inspected the results, wrote the manuscript, and revised the manuscript. WDGN assisted in the MS/MS data analysis, inspected the results, and revised the manuscript. PCPB inspected the results and revised the manuscript. AC-R performed the extraction and LC–MS/MS analyses, inspected the results, and revised the manuscript. AB inspected the results and revised the manuscript. PD designed the study, acquired funding, and revised the manuscript. VB designed the study, acquired funding, and revised the manuscript. All authors contributed to the article and approved the submitted version.

## Funding

This research was financially supported by São Paulo Research Foundation (FAPESP-CEPID, #2013/07600-3 and FAPESP-INCT, #2014/50926-0) and the Brazilian Council for Scientific and Technological Development (CNPq-INCT, #2014/465637-0). This research was supported by resources supplied by the Center for Scientific Computing (NCC/GridUNESP) of the São Paulo State University (UNESP). HM-R acknowledges CNPq (#142014/2018-4) and the Brazilian Fulbright Commission for the scholarships provided. PCPB and AB acknowledge FAPESP (grants #2017/19702-6, #2019/08477-7, and #2018/24865-4) for the research grants and scholarships provided. AC-R and PD were supported by the Gordon and Betty Moore Foundation through grant GBMF7622, the U.S. National Institutes of Health for the Center (P41 GM103484 and R01 GM107550), and Federal Award DE-SC0021340 subaward 1070261-436503.

## Conflict of Interest

The authors declare that the research was conducted in the absence of any commercial or financial relationships that could be construed as a potential conflict of interest.

## Publisher’s Note

All claims expressed in this article are solely those of the authors and do not necessarily represent those of their affiliated organizations, or those of the publisher, the editors and the reviewers. Any product that may be evaluated in this article, or claim that may be made by its manufacturer, is not guaranteed or endorsed by the publisher.

## Footnotes

^1^ http://powo.science.kew.org/

^2^ http://massive.ucsd.edu

^3^ https://github.com/biocore/q2-qemistree

^4^ https://github.com/helenamrusso/Malpighiaceae_supplementary

^5^ https://www.ncbi.nlm.nih.gov/genbank/
