# Supplementary material for: Untargeted Metabolomics Sheds Light on the Diversity of Major Classes of Secondary Metabolites in the Malpighiaceae Botanical Family
Source: Front Plant Sci. 2022 Apr 14;13:854842. doi: 10.3389/fpls.2022.854842 (PMC9047359; doi:10.3389/fpls.2022.854842)
Supplement: Supplementary file 1 [file Data_Sheet_1.DOCX]

**Supplementary Material**

**Untargeted metabolomics sheds light on the diversity of major classes of secondary metabolites in the Malpighiaceae botanical family**

Helena Mannochio-Russo^1,2,*^, Rafael F. de Almeida^3,4^, Wilhan D. G. Nunes^5^, Paula C. P. Bueno^6,7^, Andrés M. Caraballo-Rodríguez^2^, Anelize Bauermeister^2^, Pieter C. Dorrestein^2^, Vanderlan S. Bolzani^1,*^

*^1^NuBBE, Department of Biochemistry and Organic Chemistry, Institute of Chemistry, São Paulo State University (UNESP), 14800-901 Araraquara, SP, Brazil.*

*^2^Collaborative Mass Spectrometry Innovation Center, Skaggs School of Pharmacy and Pharmaceutical Sciences, University of California, San Diego, La Jolla, California 92093, United States.*

*^3^Royal Botanical Gardens Kew, Science, Ecosystem Stewardship, Diversity and Livelihoods, Richmond, Surrey, TW9 3AE, United Kingdom.*

*^4^Department of Biological Sciences, Lamol Lab, Feira de Santana State University (UEFS), Feira de Santana, BA 44036-900, Brazil.*

*^5^Federal Institute of Education, Science and Technology of Rondônia (IFRO), Ji-Paraná campus, Ji-Paraná, RO, Brazil.*

*^6^Max Planck Institute of Molecular Plant Physiology, 14476, Potsdam-Golm, Germany.*

*^7^Institute of Chemistry, Federal University of Alfenas (UNIFAL), 37130-001, Alfenas, MG, Brazil.*

***Correspondence:**

*Vanderlan da Silva Bolzani*

*vanderlan.bolzani@unesp.br*

*Helena Mannochio-Russo*

*helena.russo@unesp.br*

The following Supplementary Materials are available for this article:

**Supplementary Figure 1.** Clustering trends observed in different ionization modes for the hydroethanolic extracts and correlation with phylogeny. **(A)** Three-dimensional PCoA plots of the hydroethanolic extracts analyzed in different ionization modes determined by Canberra distance. The percentage of variance explained by the principal coordinates is presented on each axis. **(B)** Groups observed in the PCoA plots optimized using maximum likelihood criteria in the most recent molecular phylogeny of Malpighiaceae. Pie charts located on the tip of branches represent presence/absence of metabolomic profiles from groups A and B. Pie charts located on branch nodes of the tree represent the statistical results (presented in %) from the optimization analyzes. Colors represent all ten major phylogenetic clades (i.e., natural groups) currently recognized in Malpighiaceae: light yellow- Byrsonimoid clade (A); dark blue- Acridocarpoid clade (B); pink- Mcvaughioid clade (C); grey- Barnebyoid clade (D); light blue- Ptilochaetoid clade (E); dark green- Bunchosioid clade (F); purple- Hiraeoid clade (G); light green- Tetrapteroid clade (H); yellow- Malpighioid clade (I); and red- Stigmaphylloid clade (J). 5

**Supplementary Figure 2.** Clustering trends observed in different ionization modes for the ethyl acetate extracts and correlation with phylogeny. **(A)** Three-dimensional PCoA plots of the ethyl acetate extracts analyzed in positive ionization mode determined by Canberra distance. The percentage of variance explained by the principal coordinates is presented on each axis. **(B)** Three-dimensional PCoA plots of the ethyl acetate extracts analyzed in negative ionization mode determined by Canberra, Bray–Curtis and Jaccard distances. The percentages of variance explained by the principal coordinates are presented on each axis. **(C)** Groups observed in the PCoA plots optimized using maximum likelihood criteria in the most recent molecular phylogeny of Malpighiaceae. 6

**Supplementary Figure 3.** Molecular family composed of flavonoids containing two *C*-glycosylated portions (positive ionization mode). 7

**Supplementary Figure 4.** Molecular family composed of flavonoids containing one *C*-glycosylated portion (positive ionization mode). 7

**Supplementary Figure 5.** Molecular family composed of *O*-glycosylated flavonoids (positive ionization mode). 8

**Supplementary Figure 6.** Molecular family composed of O-glycosylated flavonoids containing phenylpropanoid potions (positive ionization mode). 8

**Supplementary Figure 7.** Molecular family composed of *O*-glycosylated flavonoids containing galloyl potions (positive ionization mode). 9

**Supplementary Figure 8.** Molecular family mainly composed of non-glycosylated flavonoids (positive ionization mode). 10

**Supplementary Figure 9.** Molecular family mainly composed of catechin, afzelechin and their derivatives (positive ionization mode). 10

**Supplementary Figure 10.** Molecular family composed of quinic acid bound to phenylpropanoids substituents (positive ionization mode). 11

**Supplementary Figure 11.** Molecular family composed of quinic acid bound to galloyl substituents (positive ionization mode). 12

**Supplementary Figure 12.** Molecular families composed of glycerophospholipids (positive ionization mode). 13

**Supplementary Figure 13.** Molecular family composed of fatty acids and fatty esters (positive ionization mode). 14

**Supplementary Figure 14.** Molecular family composed of jasmonic acid derivatives (positive ionization mode). 14

**Supplementary Figure 15.** Molecular families composed of beta-carboline alkaloids and other tryptophan derivatives (positive ionization mode). 15

**Supplementary Figure 16.** Molecular family composed of isoquinoline alkaloids (positive ionization mode). 16

**Supplementary Figure 17.** Molecular family composed of protoberberine alkaloids (positive ionization mode). 16

**Supplementary Figure 18.** Molecular family composed of benzylisoquinoline alkaloids (positive ionization mode). 17

**Supplementary Figure 19.** Molecular families composed of amides (positive ionization mode). 18

**Supplementary Figure 20.** Molecular family composed of polyamines (positive ionization mode). 19

**Supplementary Figure 21.** Molecular family composed of triterpenoids and precursors (positive ionization mode). 19

**Supplementary Figure 22.** Molecular family composed of ecdysteroids (positive ionization mode). 20

**Supplementary Figure 23.** Molecular families composed of iridoids (positive ionization mode). 21

**Supplementary Figure 24.** Molecular family composed of secoiridoids (positive ionization mode). 21

**Supplementary Figure 25.** Molecular families composed of neolignans and furofuranoid lignans (positive ionization mode). 22

**Supplementary Figure 26.** Molecular families composed of condensed tannins (negative ionization mode). 23

**Supplementary Figure 27.** Molecular families composed of lignans (negative ionization mode). 23

**Supplementary Figure 28.** Compound classification distribution within Malpighiaceae species in positive and negative ionization modes at a **(A)** CANOPUS superclass level and **(B)** CANOPUS class level. 24

**Supplementary Figure 29.** Heatmap of the normalized ion features putatively annotated at a class level based on *in silico* classification using CANOPUS for the **(A)** positive and **(B)** negative ionization modes. The classes were filtered to keep only the classes with a high correlation with the most sampled clades. The cladogram on the left reflects the latest molecular phylogeny of the Malpighiaceae family. 25

**Supplementary Table 1.** Detailed information of the Malpighiaceae sampling: species list, collection site, location and date, biome, and phylogenetic group classification. Samples consisted of plant leaves, unless specified otherwise...............................................................26

**Supplementary Table 2.** Characters retrieved from the ancestral characters reconstruction (all clades and genera) based on the classifications obtained *in silico* for Malpighiaceae samples. The ionization mode in which each classification was obtained is described (pos = positive ionization mode; neg = negative ionization mode; both = both ionization modes). 47


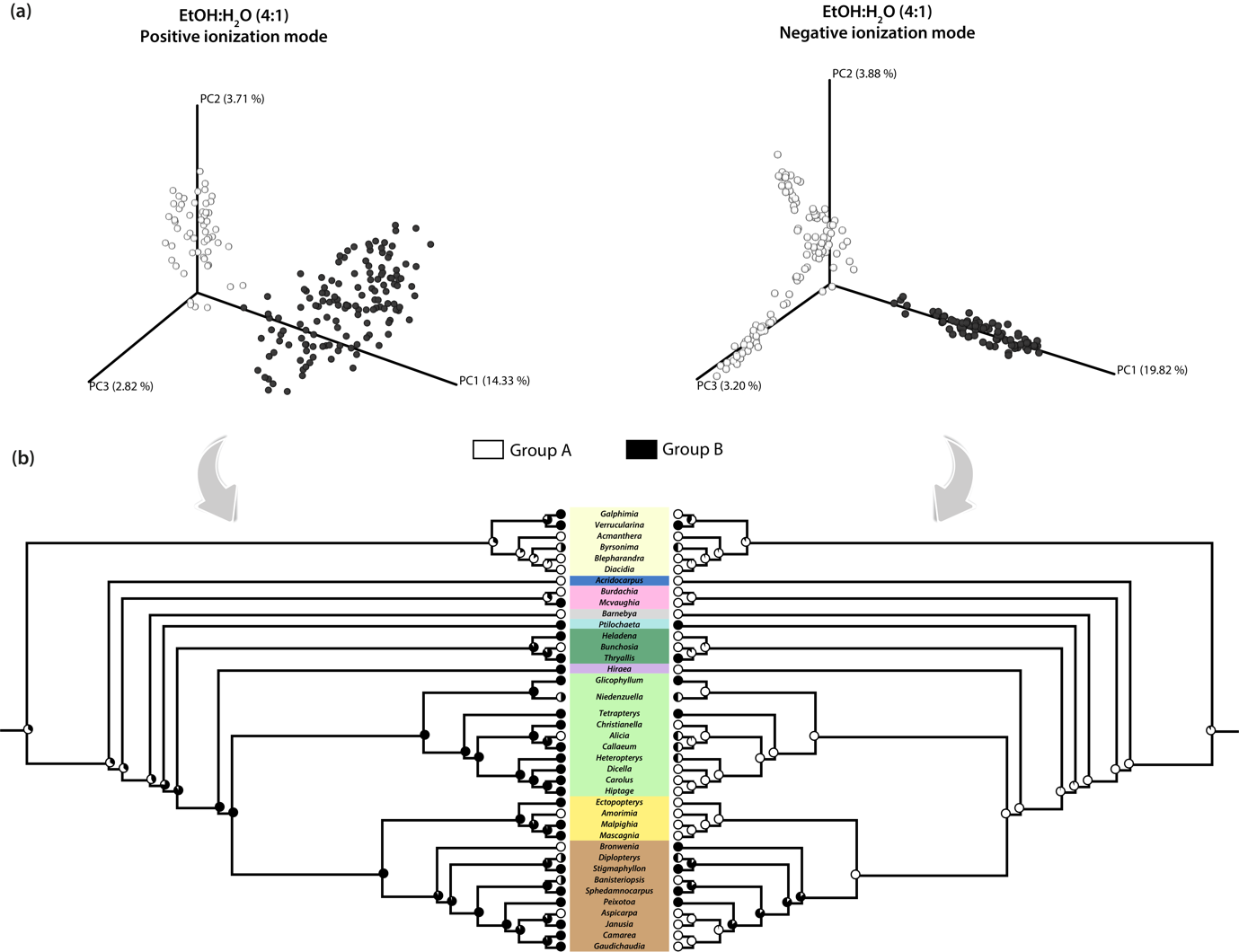


**Supplementary Figure 1.** Clustering trends observed in different ionization modes for the hydroethanolic extracts and correlation with phylogeny. **(A)** Three-dimensional PCoA plots of the hydroethanolic extracts analyzed in different ionization modes determined by Canberra distance. The percentage of variance explained by the principal coordinates is presented on each axis. **(B)** Groups observed in the PCoA plots optimized using maximum likelihood criteria in the most recent molecular phylogeny of Malpighiaceae. Pie charts located on the tip of branches represent presence/absence of metabolomic profiles from groups A and B. Pie charts located on branch nodes of the tree represent the statistical results (presented in %) from the optimization analyzes. Colors represent all ten major phylogenetic clades (i.e., natural groups) currently recognized in Malpighiaceae: light yellow- Byrsonimoid clade (A); dark blue- Acridocarpoid clade (B); pink- Mcvaughioid clade (C); grey- Barnebyoid clade (D); light blue- Ptilochaetoid clade (E); dark green- Bunchosioid clade (F); purple- Hiraeoid clade (G); light green- Tetrapteroid clade (H); yellow- Malpighioid clade (I); and red- Stigmaphylloid clade (J).


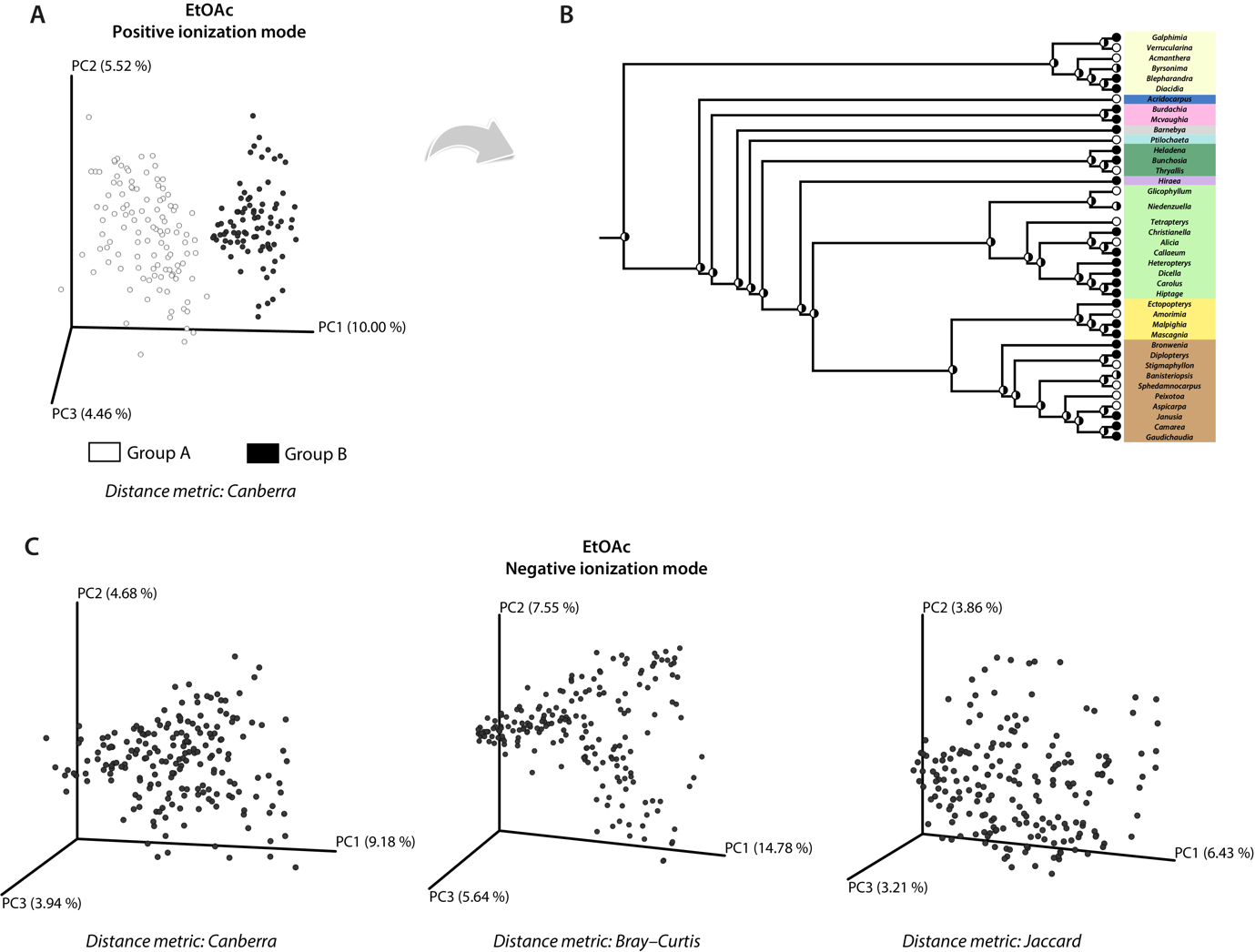


**Supplementary Figure 2.** Clustering trends observed in different ionization modes for the ethyl acetate extracts and correlation with phylogeny. **(A)** Three-dimensional PCoA plots of the ethyl acetate extracts analyzed in positive ionization mode determined by Canberra distance. The percentage of variance explained by the principal coordinates is presented on each axis. **(B)** Three-dimensional PCoA plots of the ethyl acetate extracts analyzed in negative ionization mode determined by Canberra, Bray–Curtis and Jaccard distances. The percentages of variance explained by the principal coordinates are presented on each axis. **(C)** Groups observed in the PCoA plots optimized using maximum likelihood criteria in the most recent molecular phylogeny of Malpighiaceae.


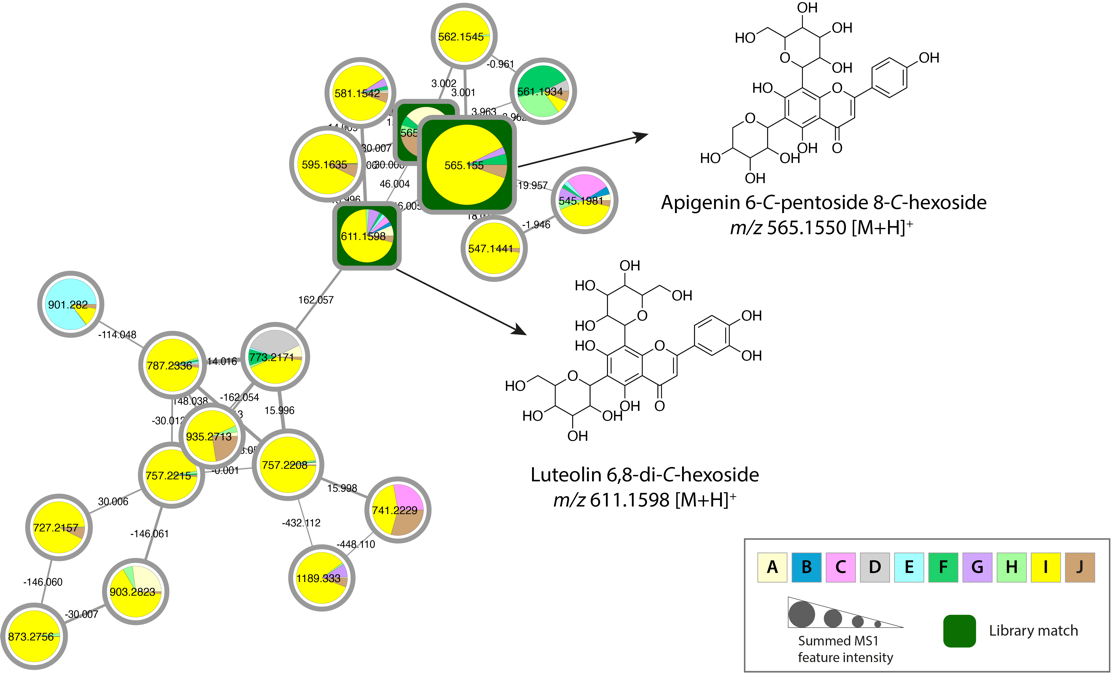


**Supplementary Figure 3.** Molecular family composed of flavonoids containing two *C*-glycosylated portions (positive ionization mode).


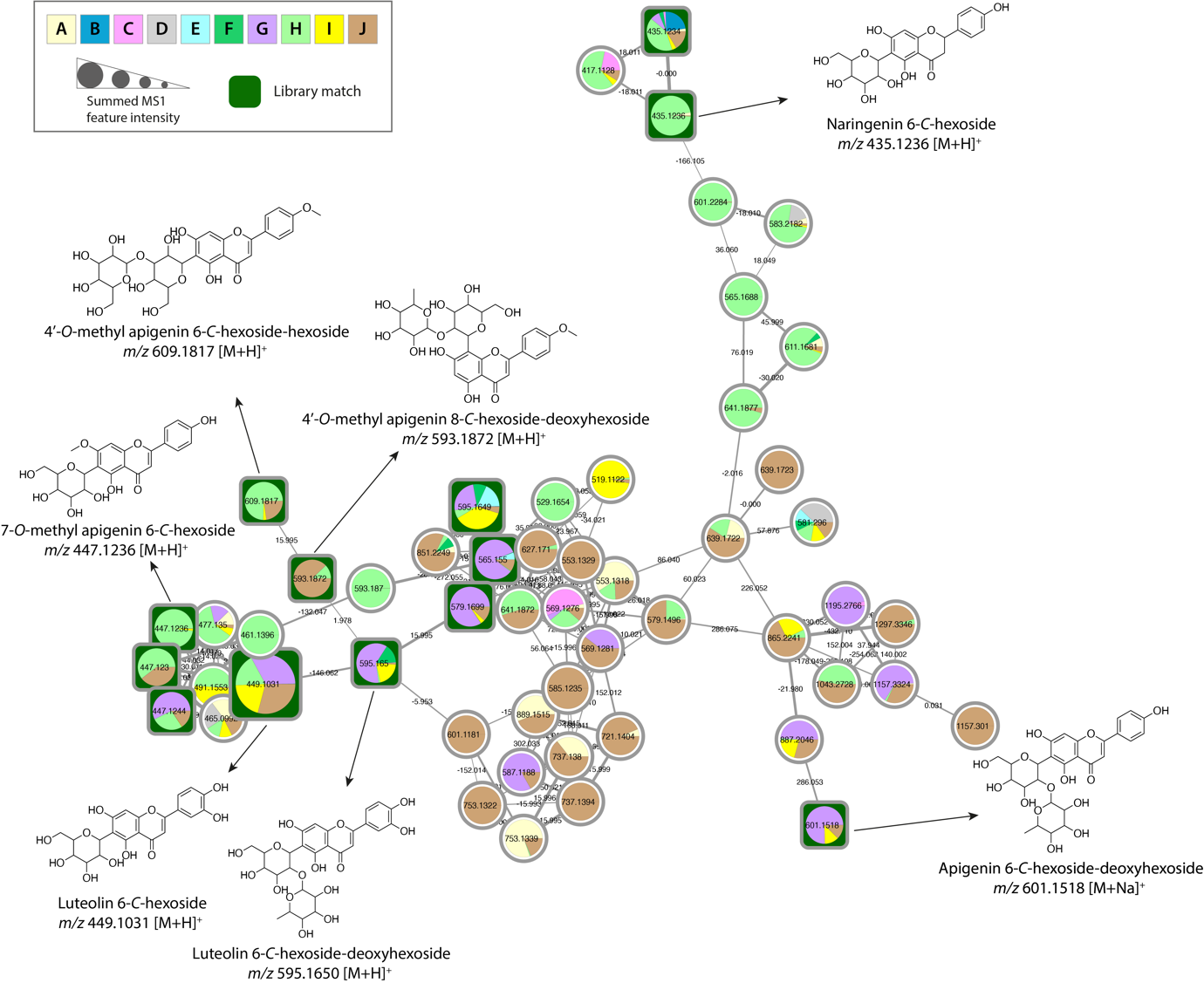


**Supplementary Figure 4.** Molecular family composed of flavonoids containing one *C*-glycosylated portion (positive ionization mode).
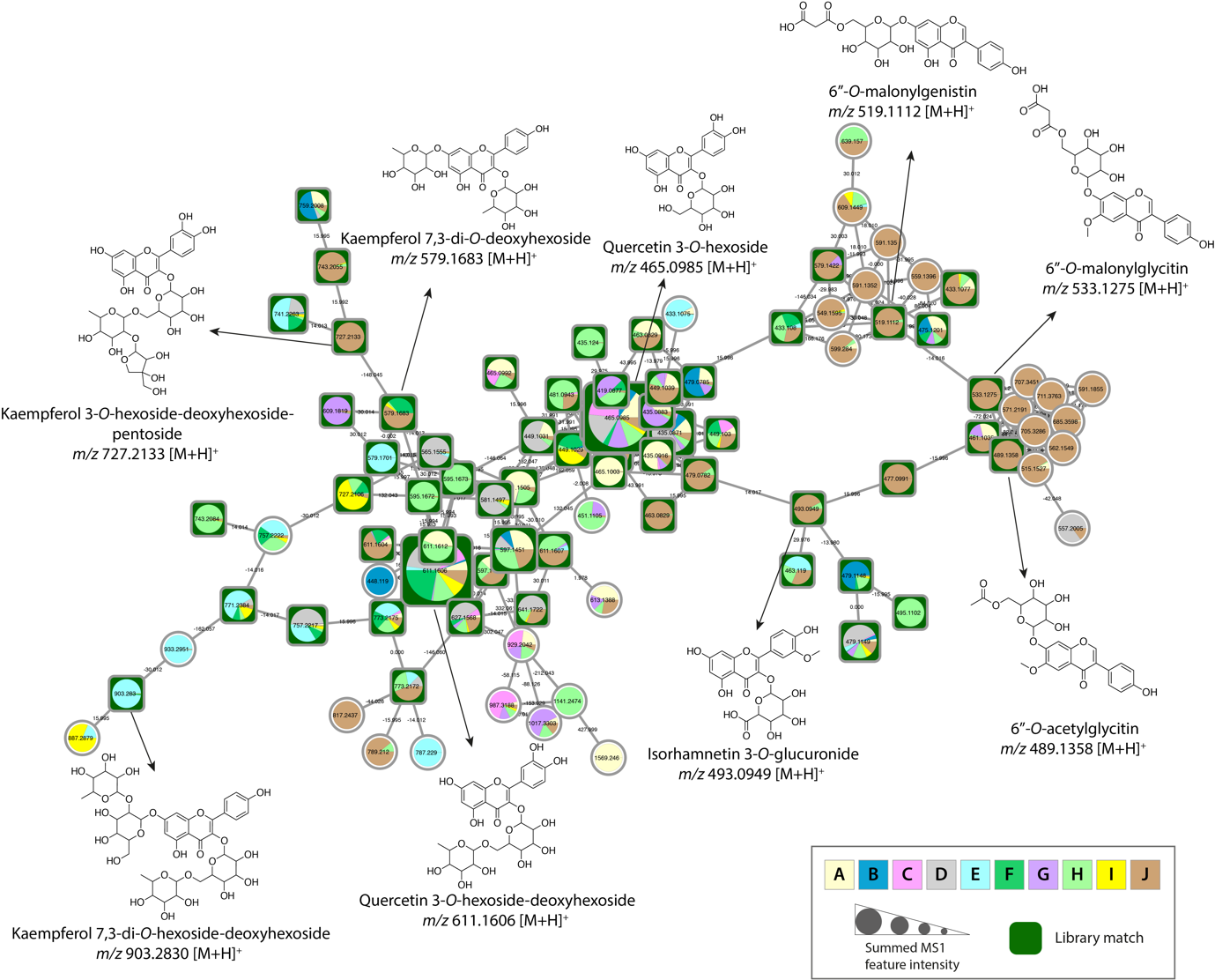


**Supplementary Figure 5.** Molecular family composed of *O*-glycosylated flavonoids (positive ionization mode).


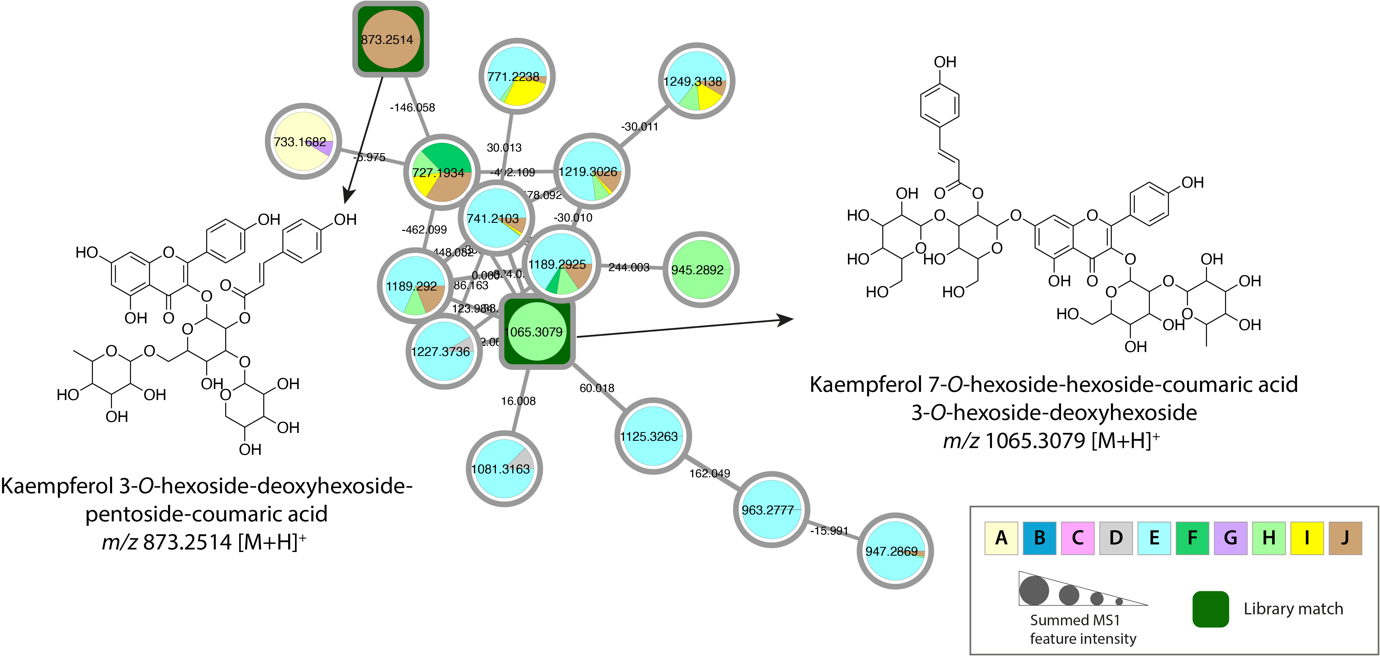


**Supplementary Figure 6.** Molecular family composed of O-glycosylated flavonoids containing phenylpropanoid potions (positive ionization mode).


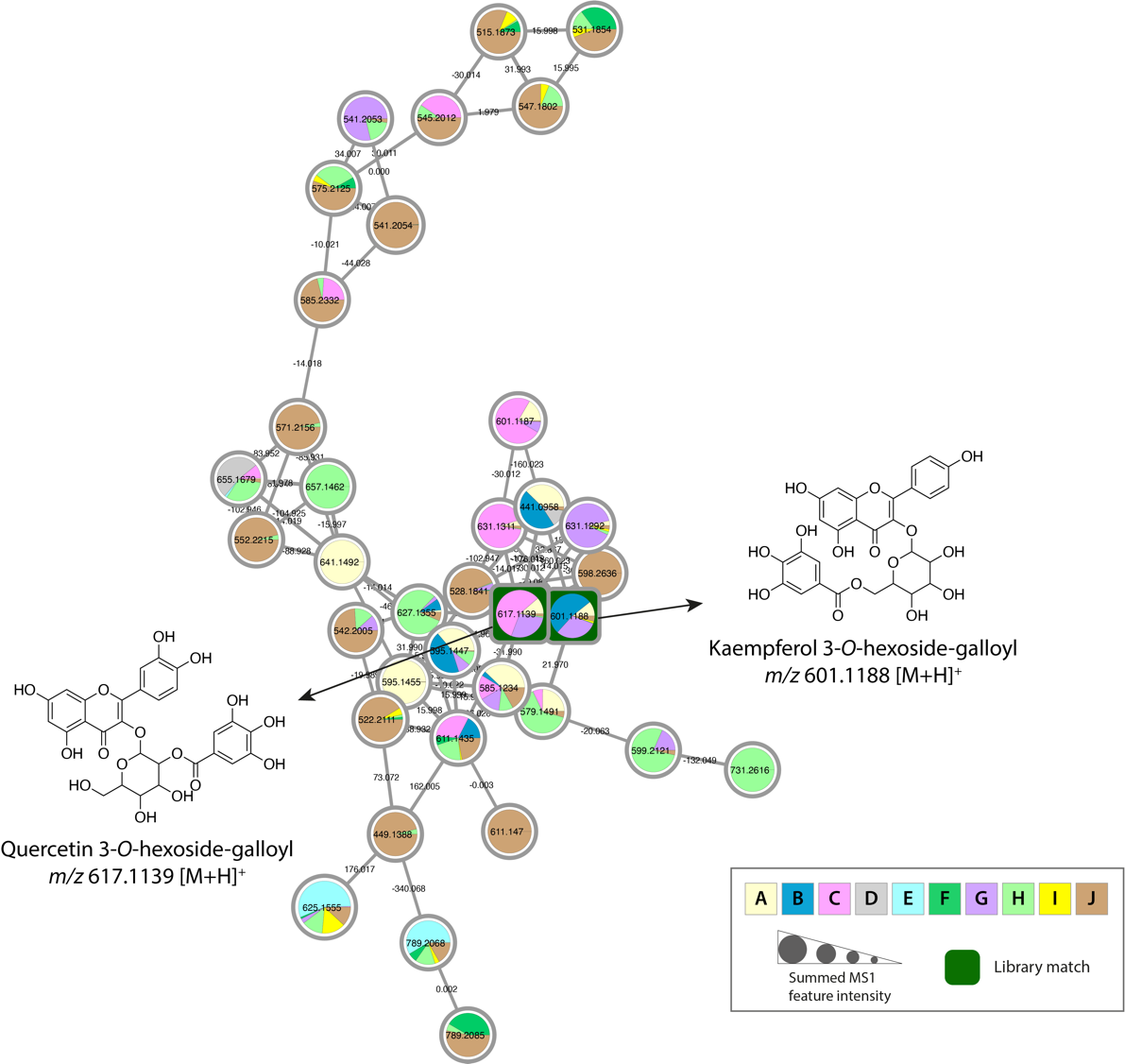


**Supplementary Figure 7.** Molecular family composed of *O*-glycosylated flavonoids containing galloyl potions (positive ionization mode).


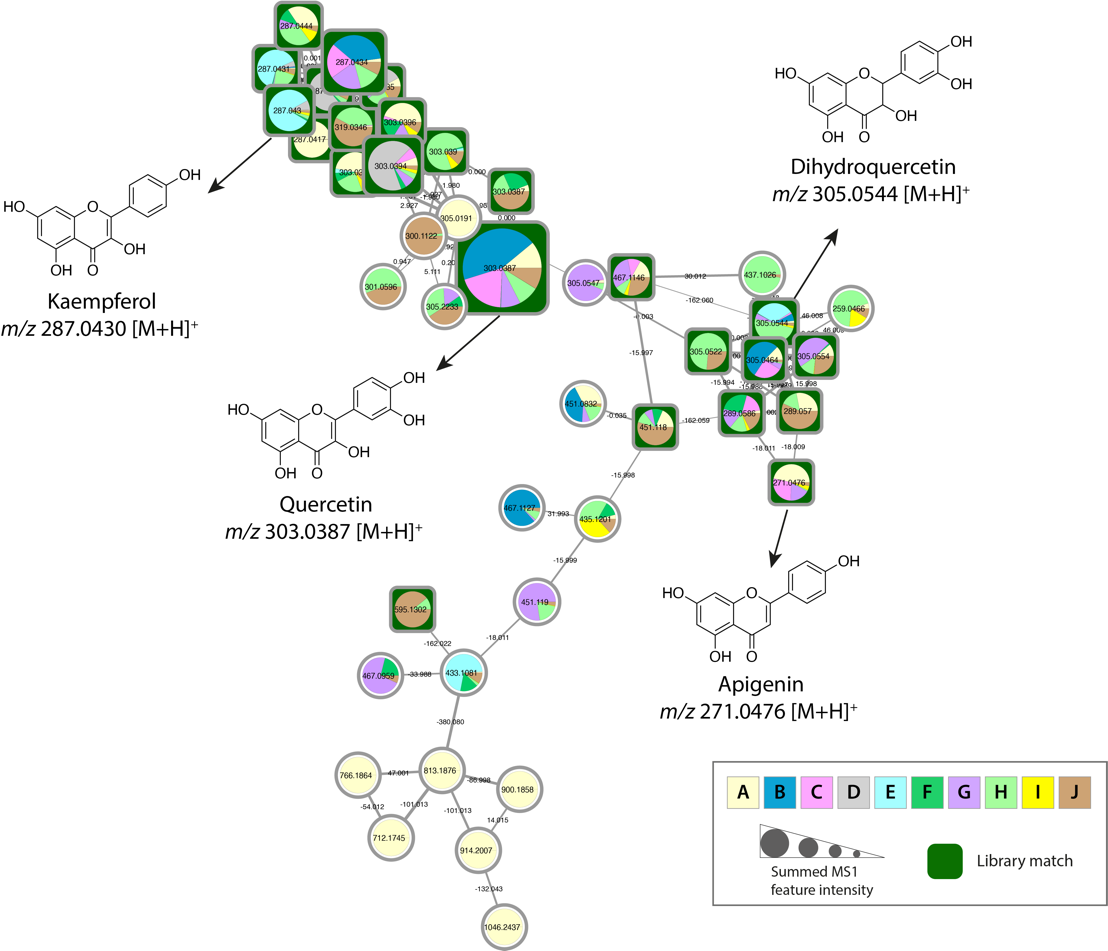


**Supplementary Figure 8.** Molecular family mainly composed of non-glycosylated flavonoids (positive ionization mode).


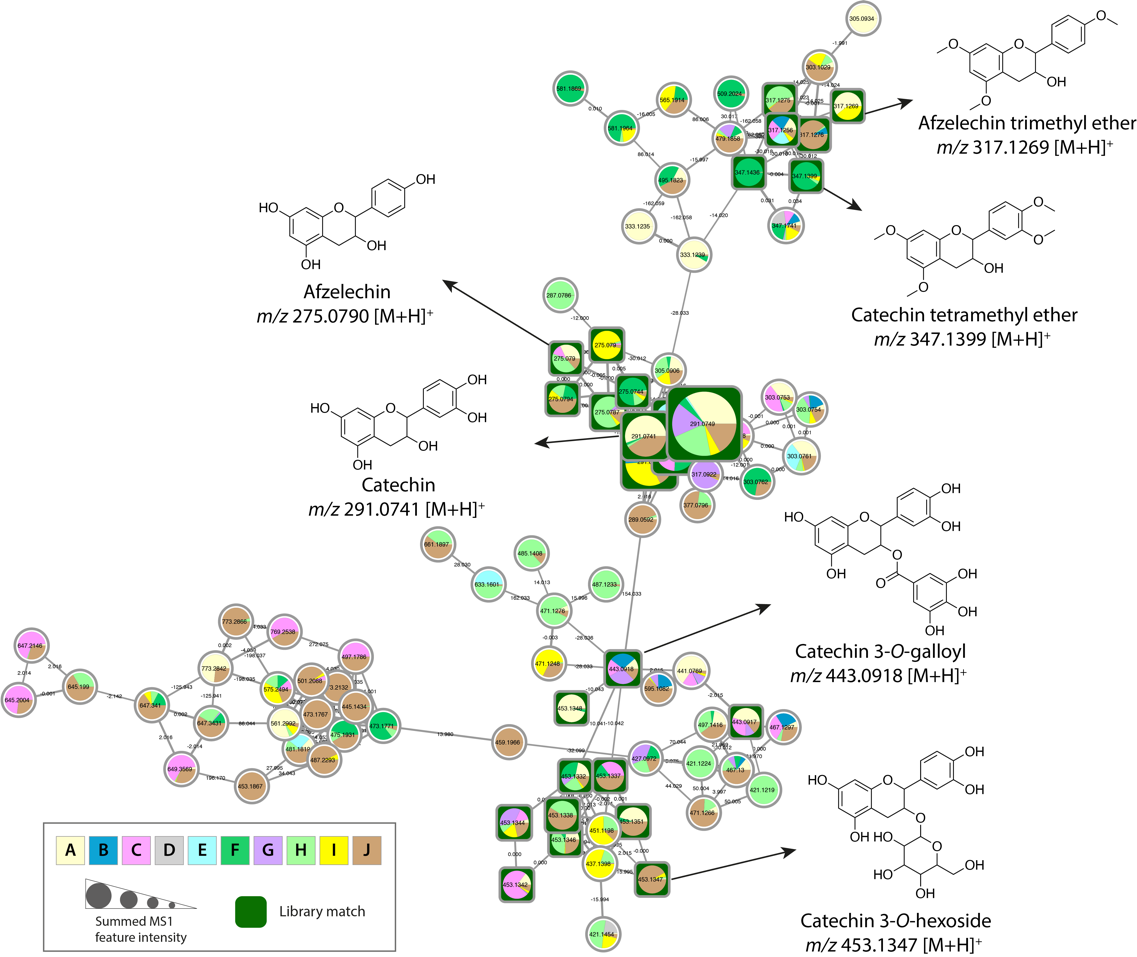


**Supplementary Figure 9.** Molecular family mainly composed of catechin, afzelechin and their derivatives (positive ionization mode).


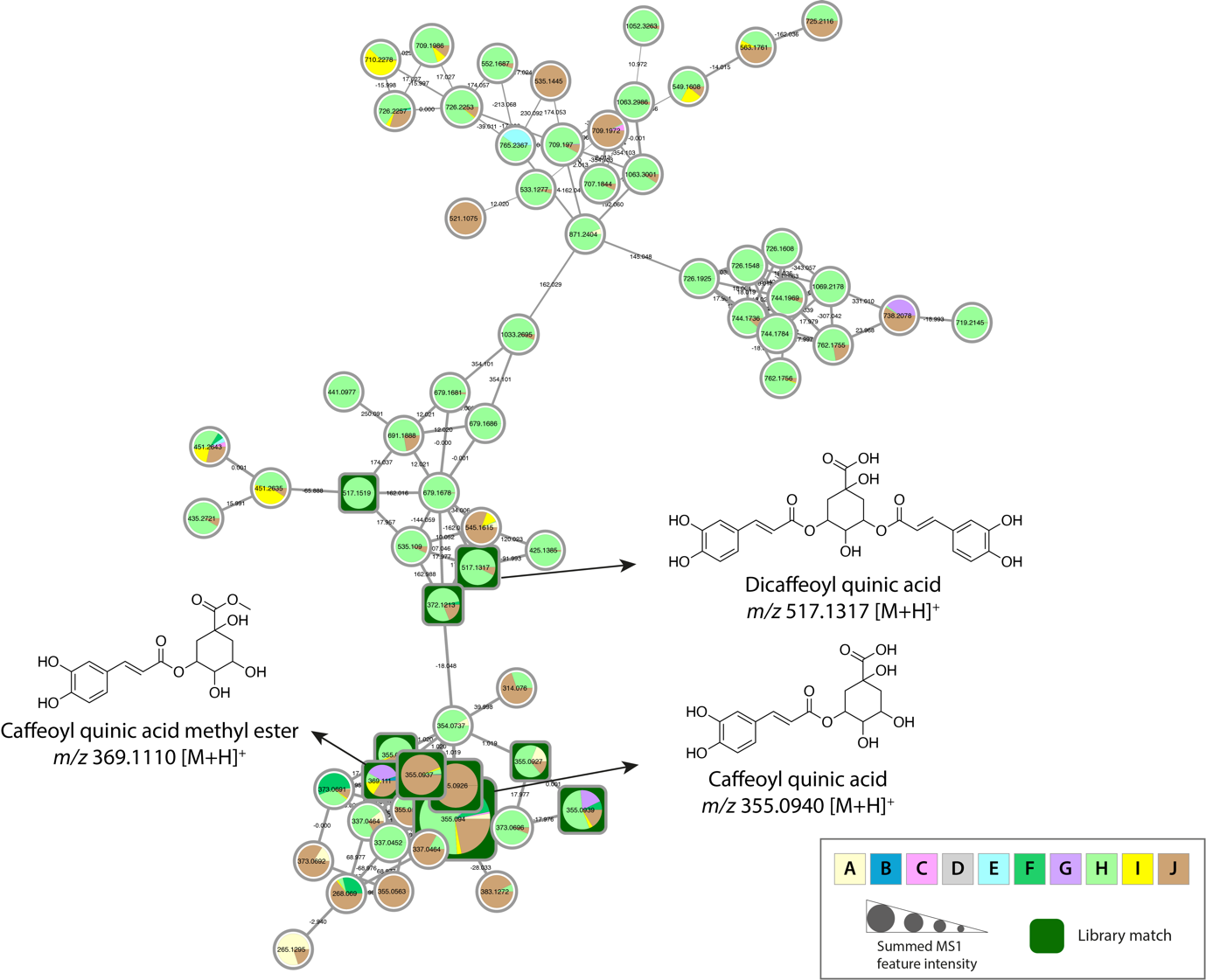


**Supplementary Figure 10.** Molecular family composed of quinic acid bound to phenylpropanoids substituents (positive ionization mode).


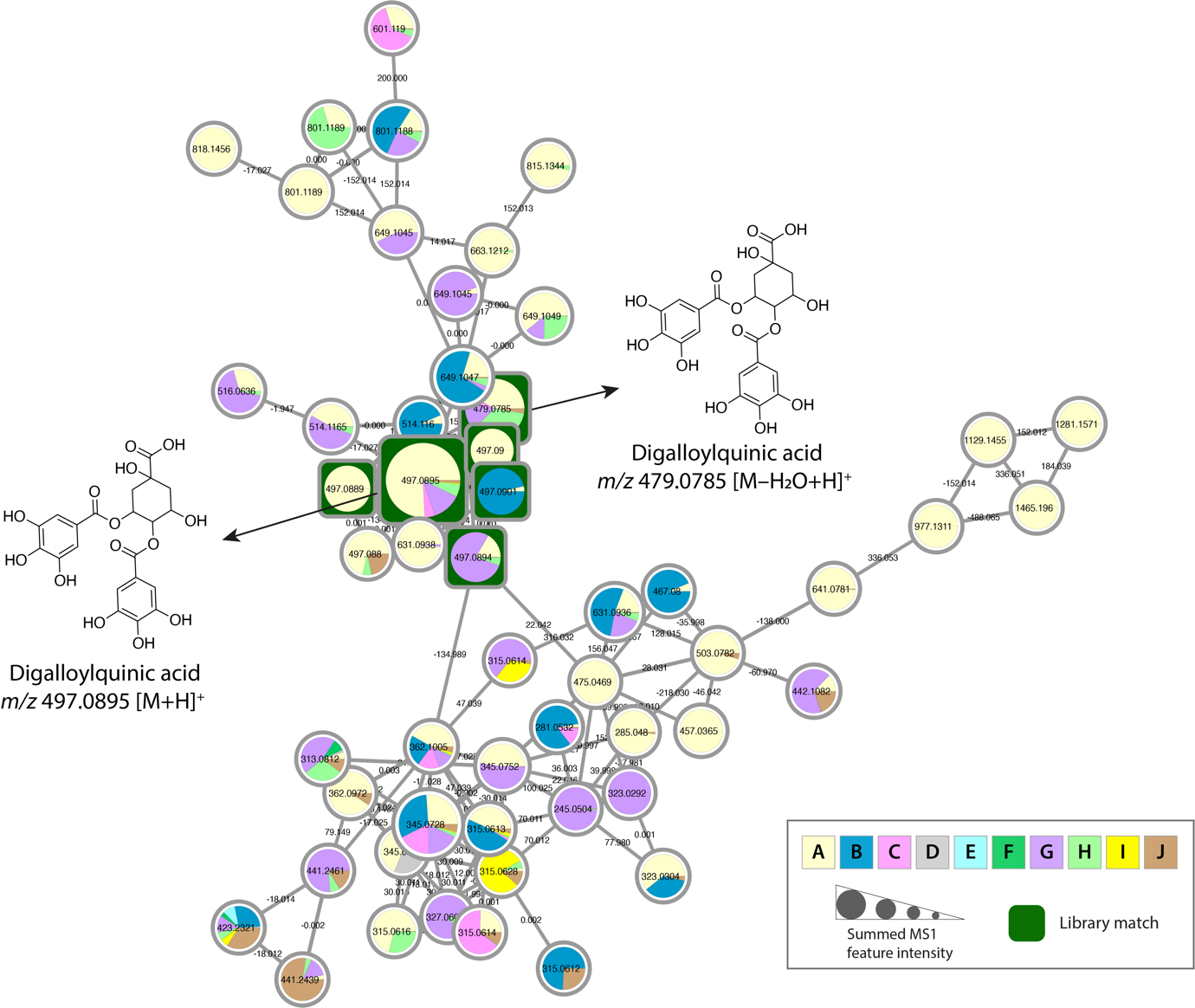


**Supplementary Figure 11.** Molecular family composed of quinic acid bound to galloyl substituents (positive ionization mode).


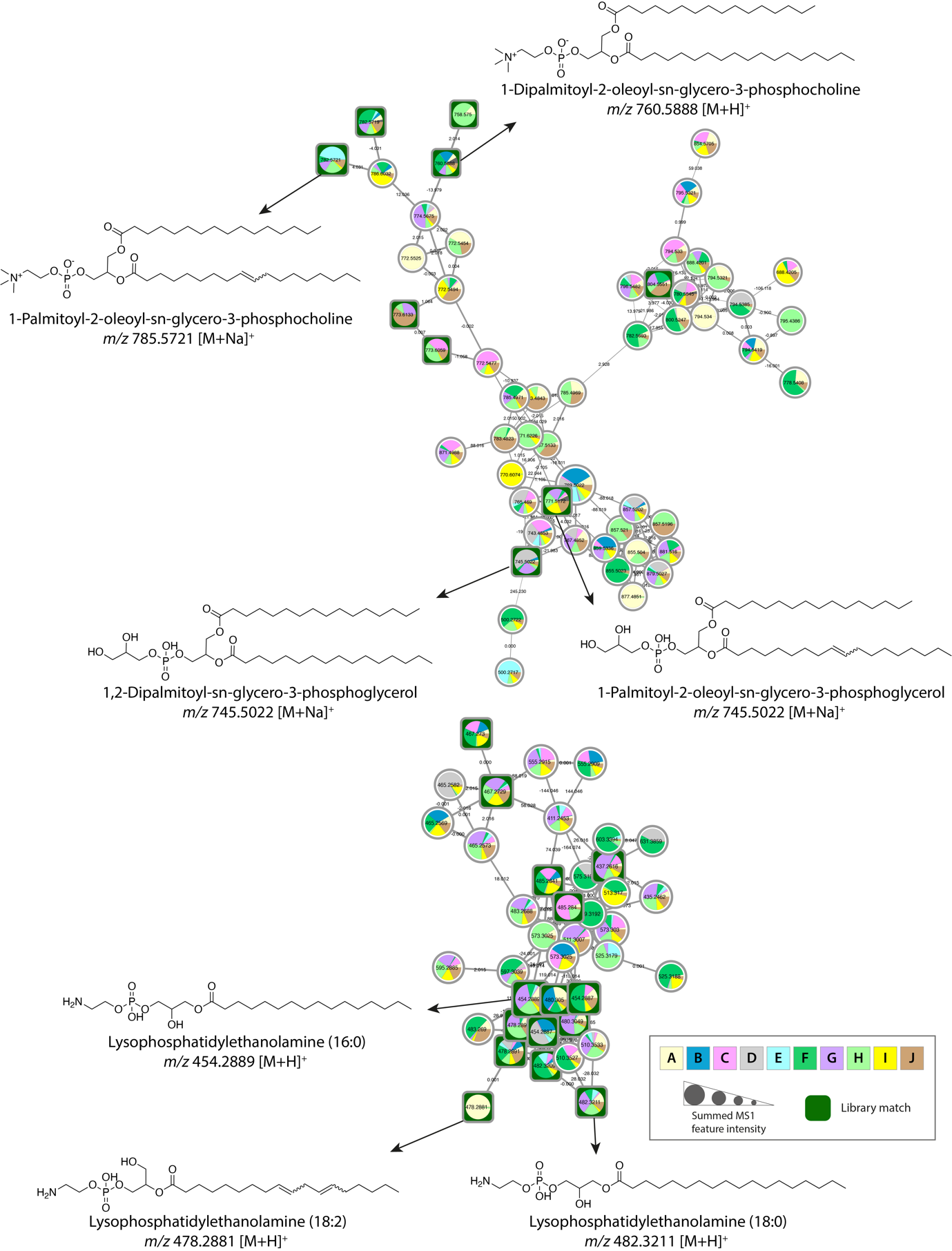


**Supplementary Figure 12.** Molecular families composed of glycerophospholipids (positive ionization mode).


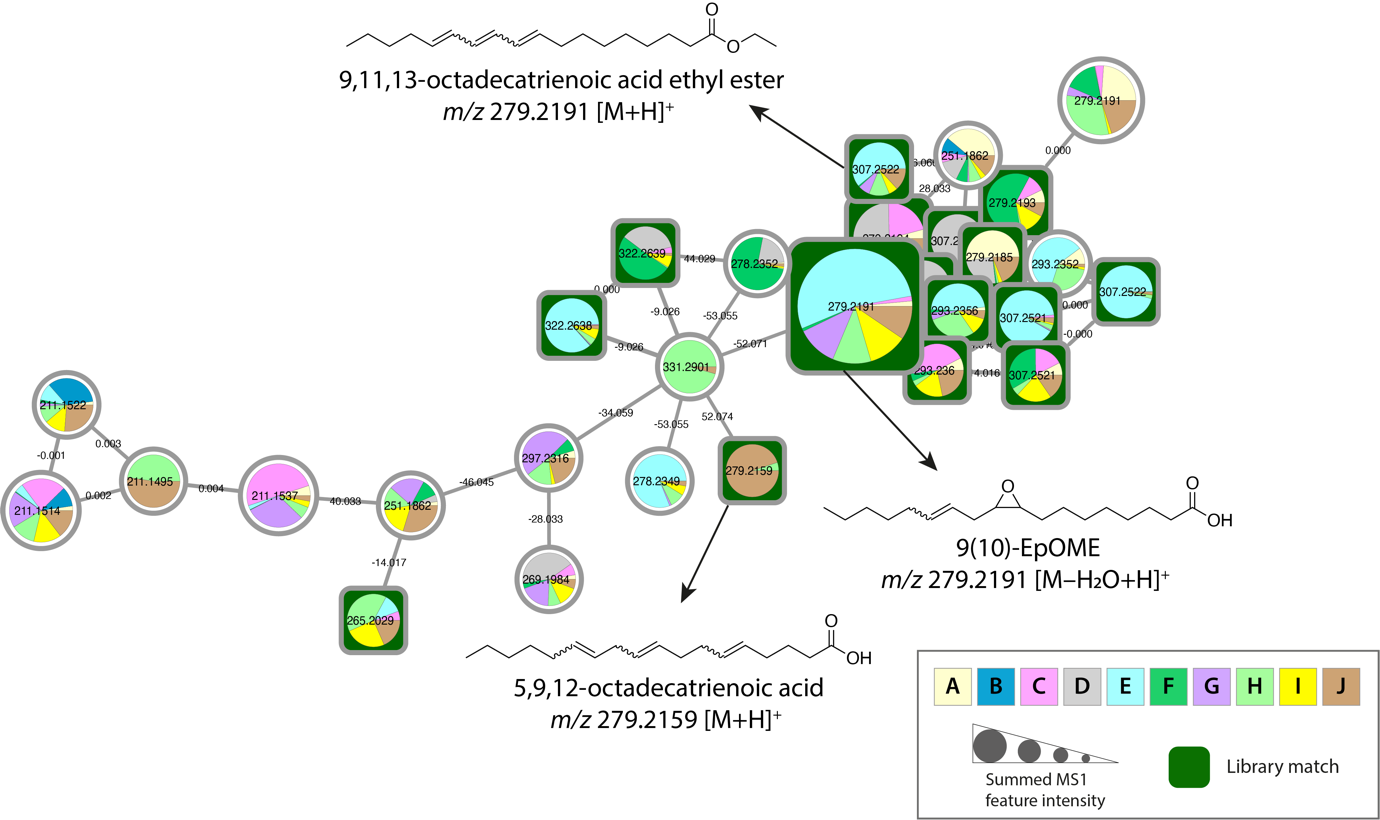


**Supplementary Figure 13.** Molecular family composed of fatty acids and fatty esters (positive ionization mode).


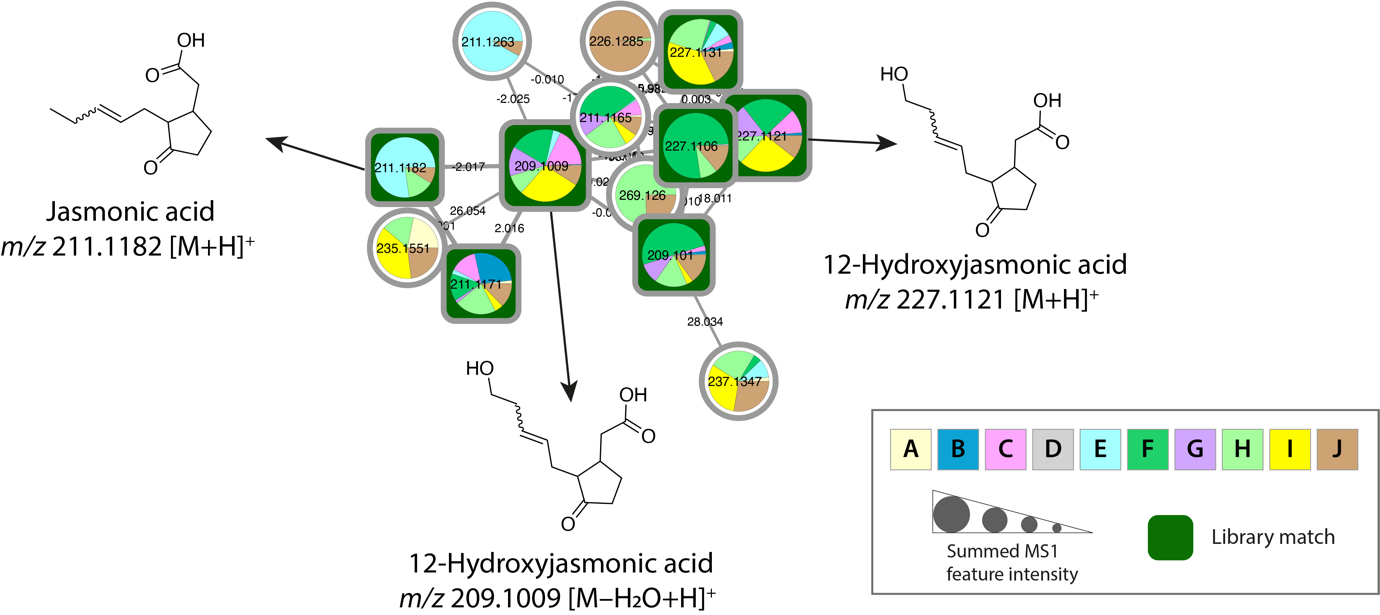


**Supplementary Figure 14.** Molecular family composed of jasmonic acid derivatives (positive ionization mode).


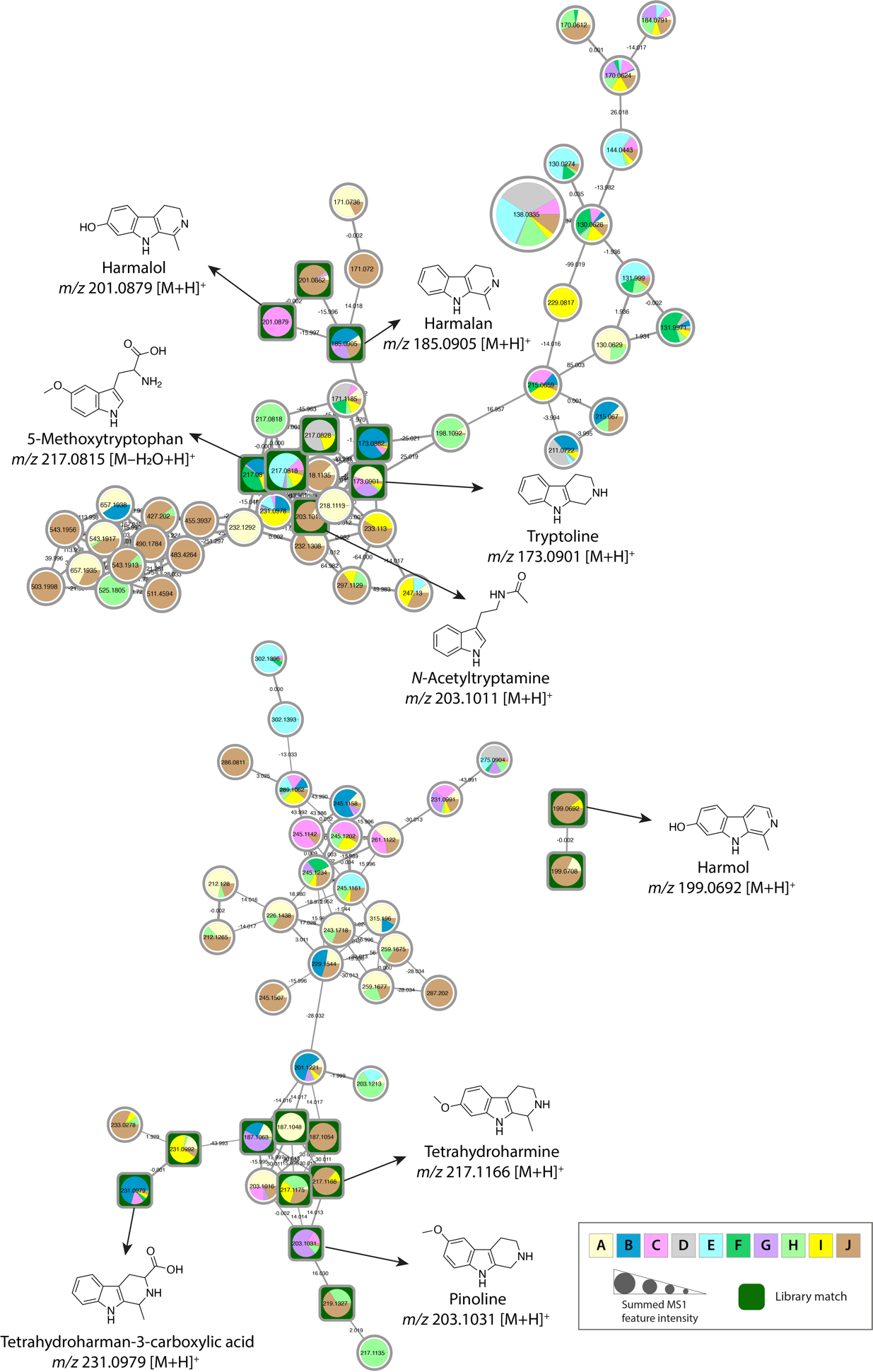


**Supplementary Figure 15.** Molecular families composed of beta-carboline alkaloids and other tryptophan derivatives (positive ionization mode).


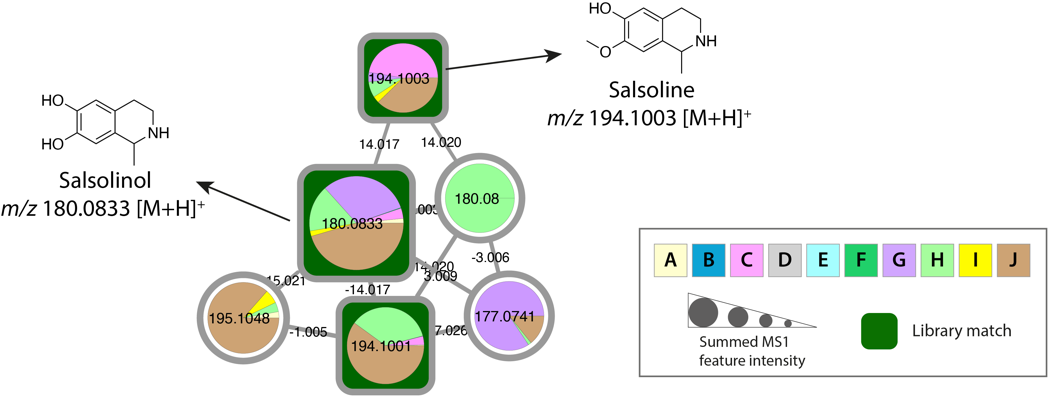


**Supplementary Figure 16.** Molecular family composed of isoquinoline alkaloids (positive ionization mode).


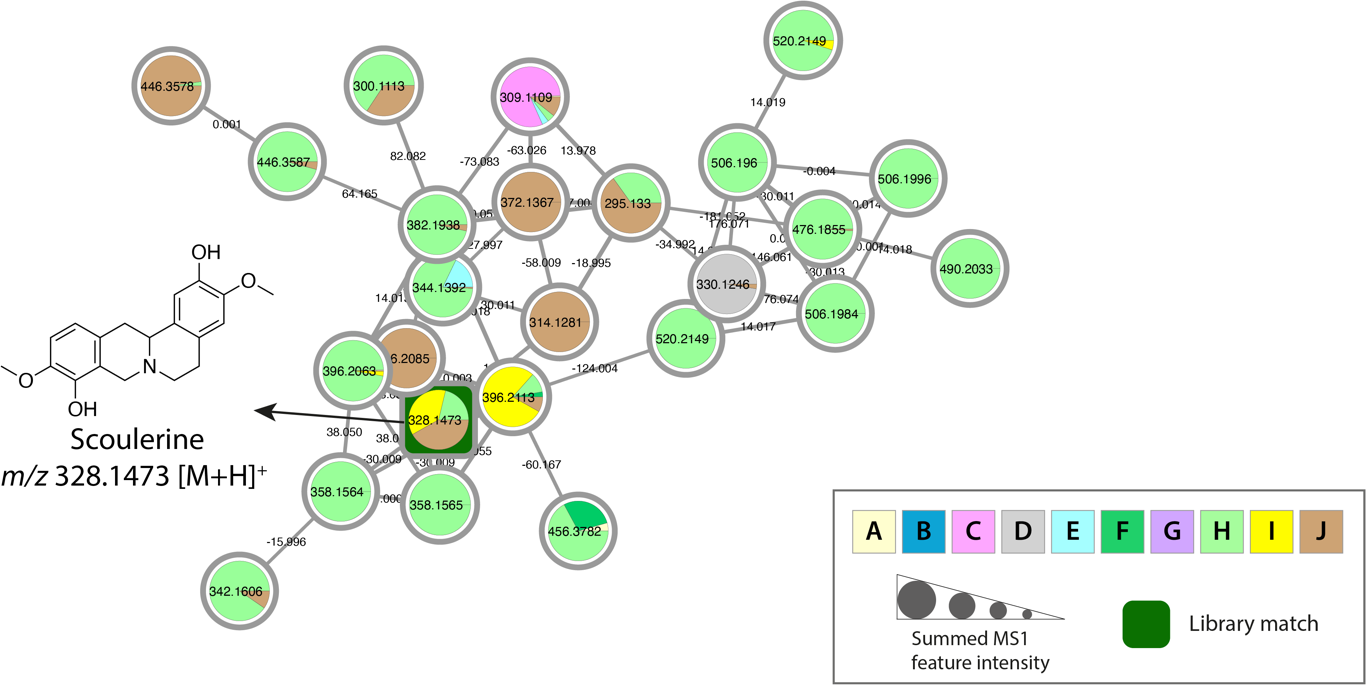


**Supplementary Figure 17.** Molecular family composed of protoberberine alkaloids (positive ionization mode).


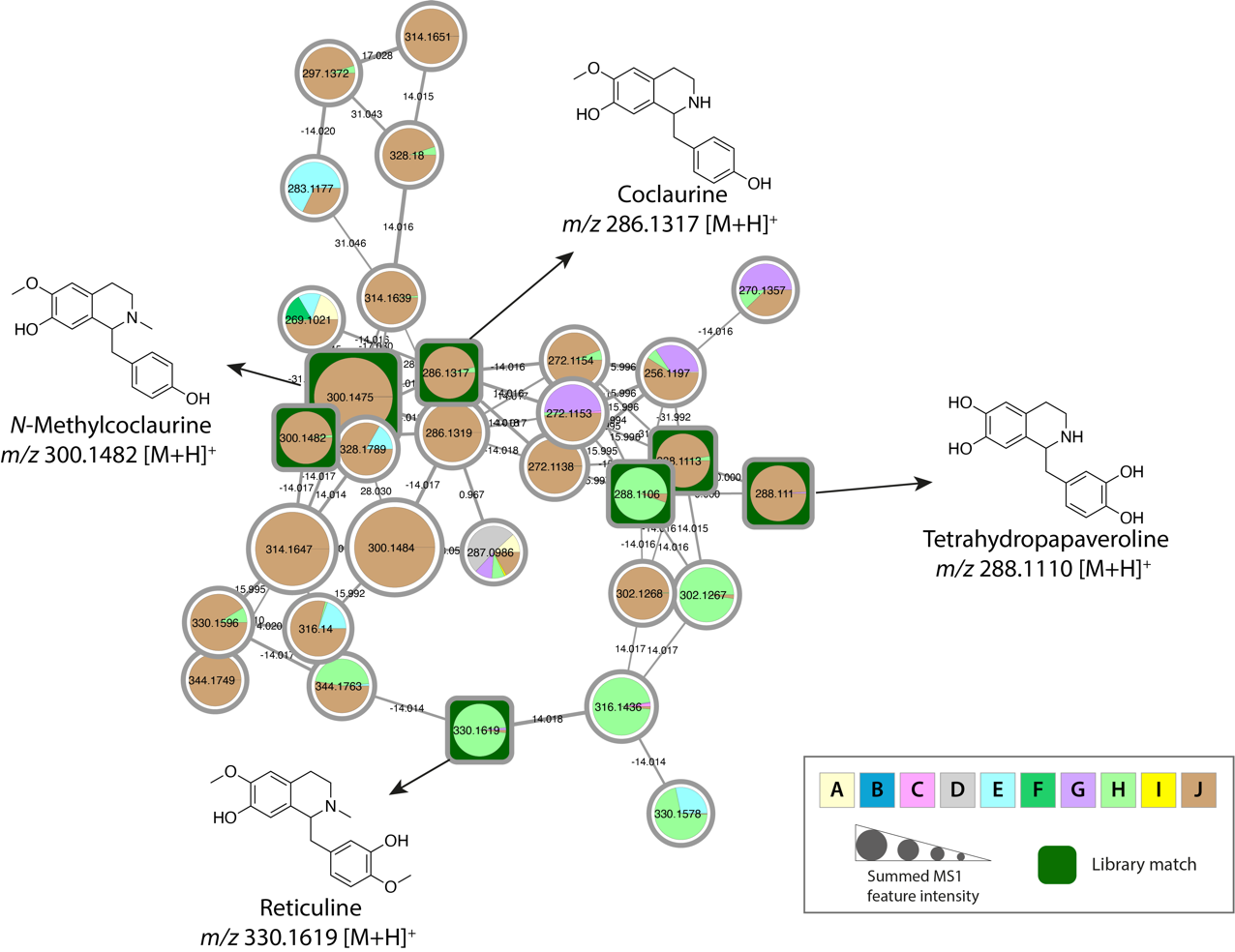


**Supplementary Figure 18.** Molecular family composed of benzylisoquinoline alkaloids (positive ionization mode).


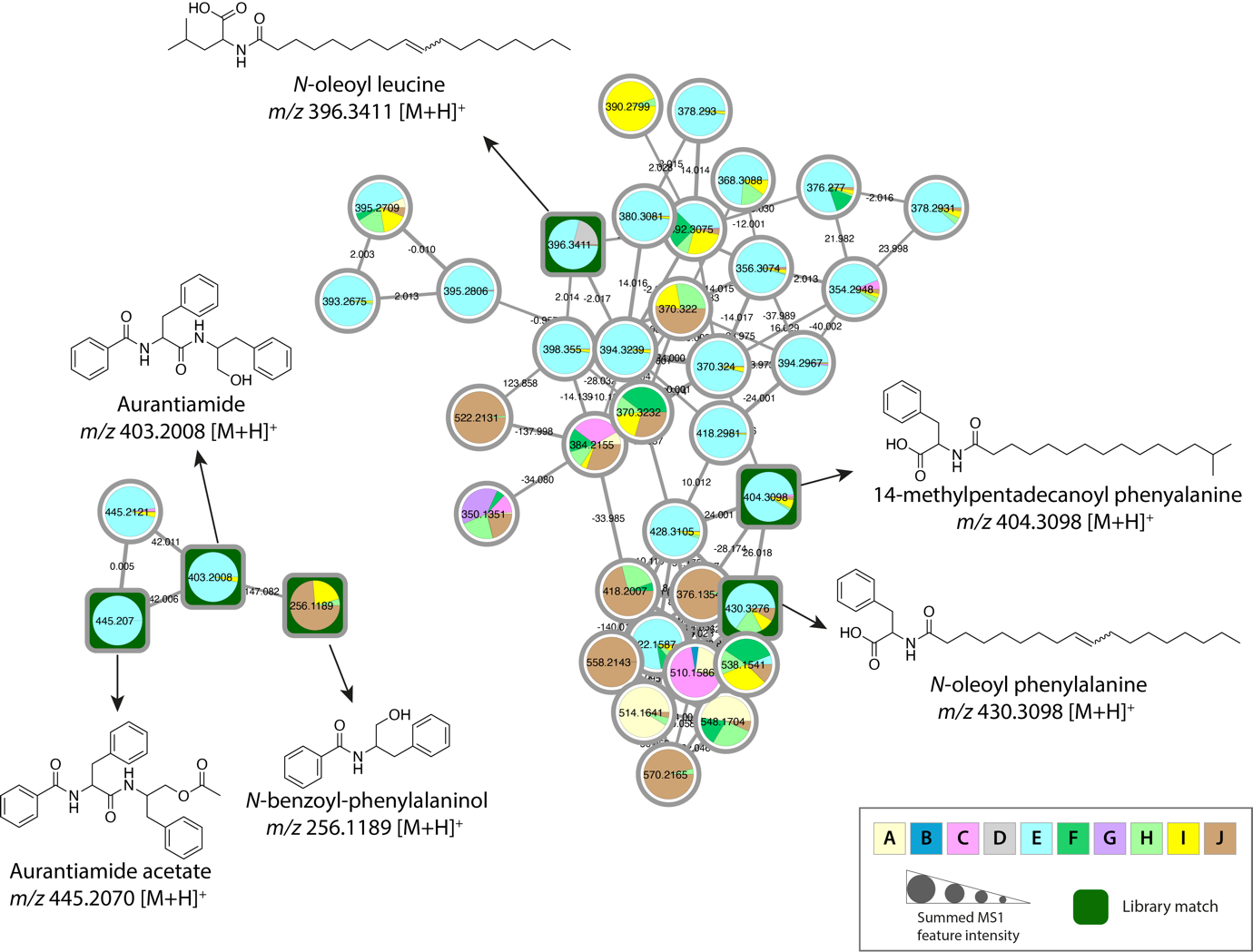


**Supplementary Figure 19.** Molecular families composed of amides (positive ionization mode).


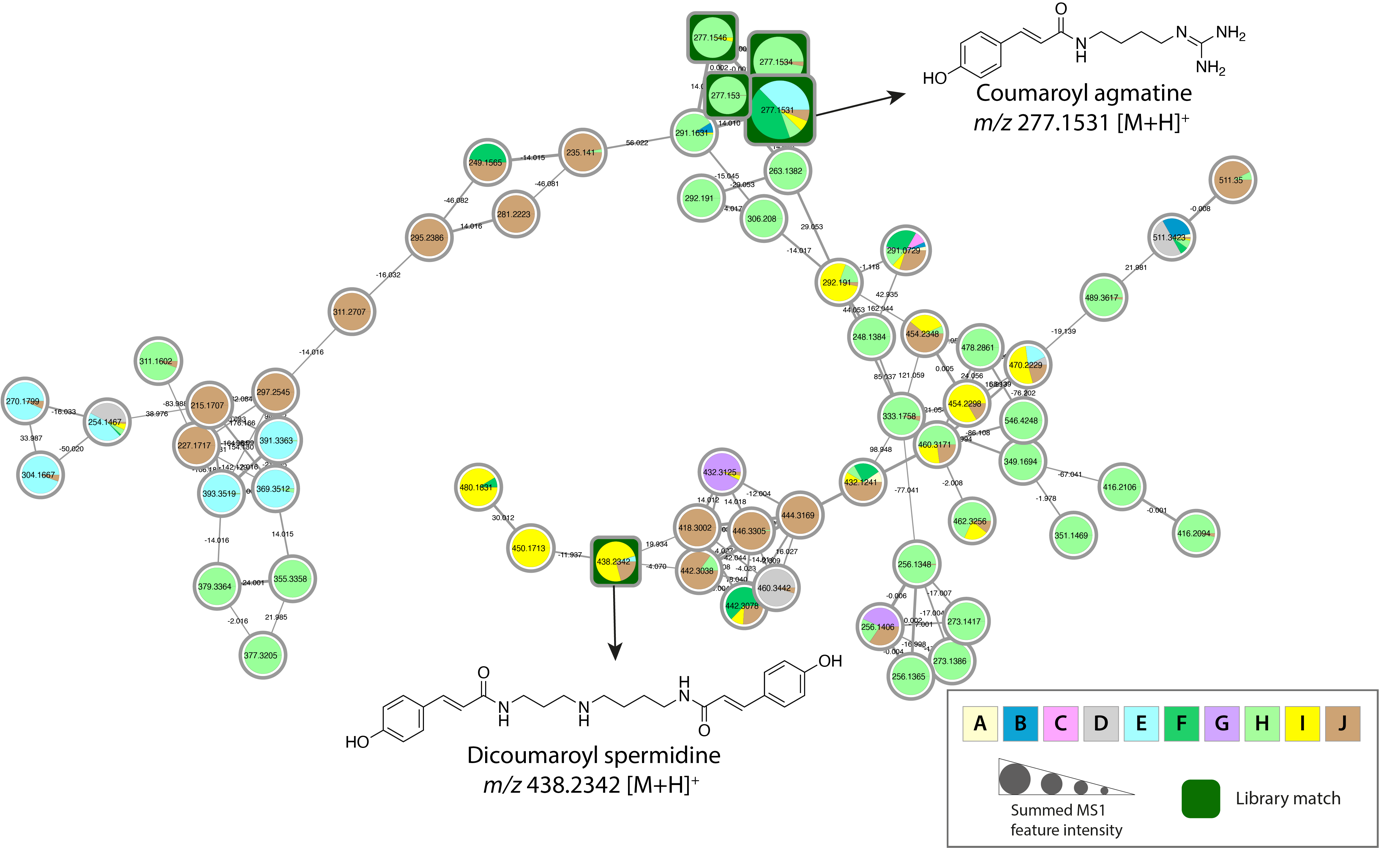


**Supplementary Figure 20.** Molecular family composed of polyamines (positive ionization mode).


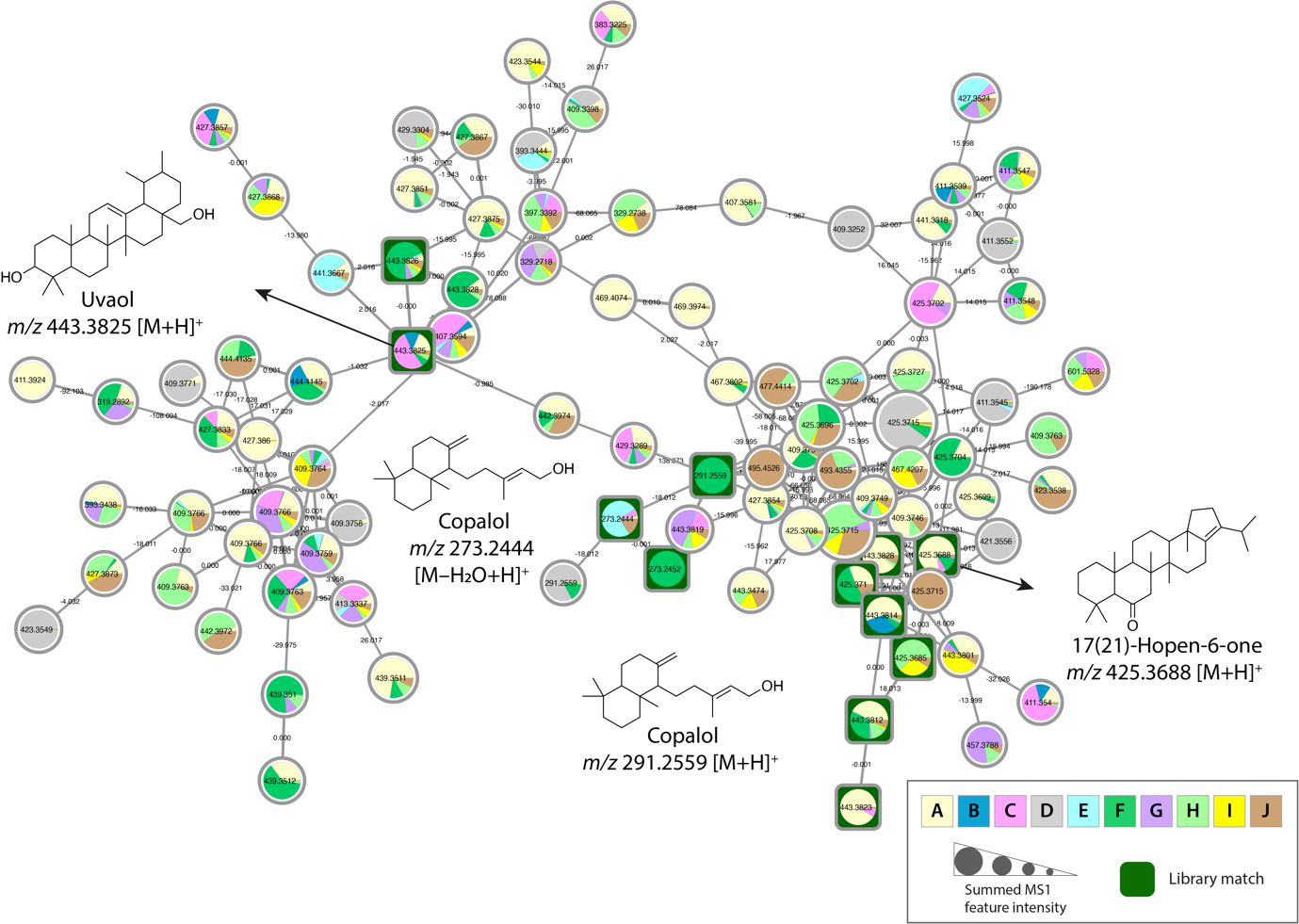


**Supplementary Figure 21.** Molecular family composed of triterpenoids and precursors (positive ionization mode).


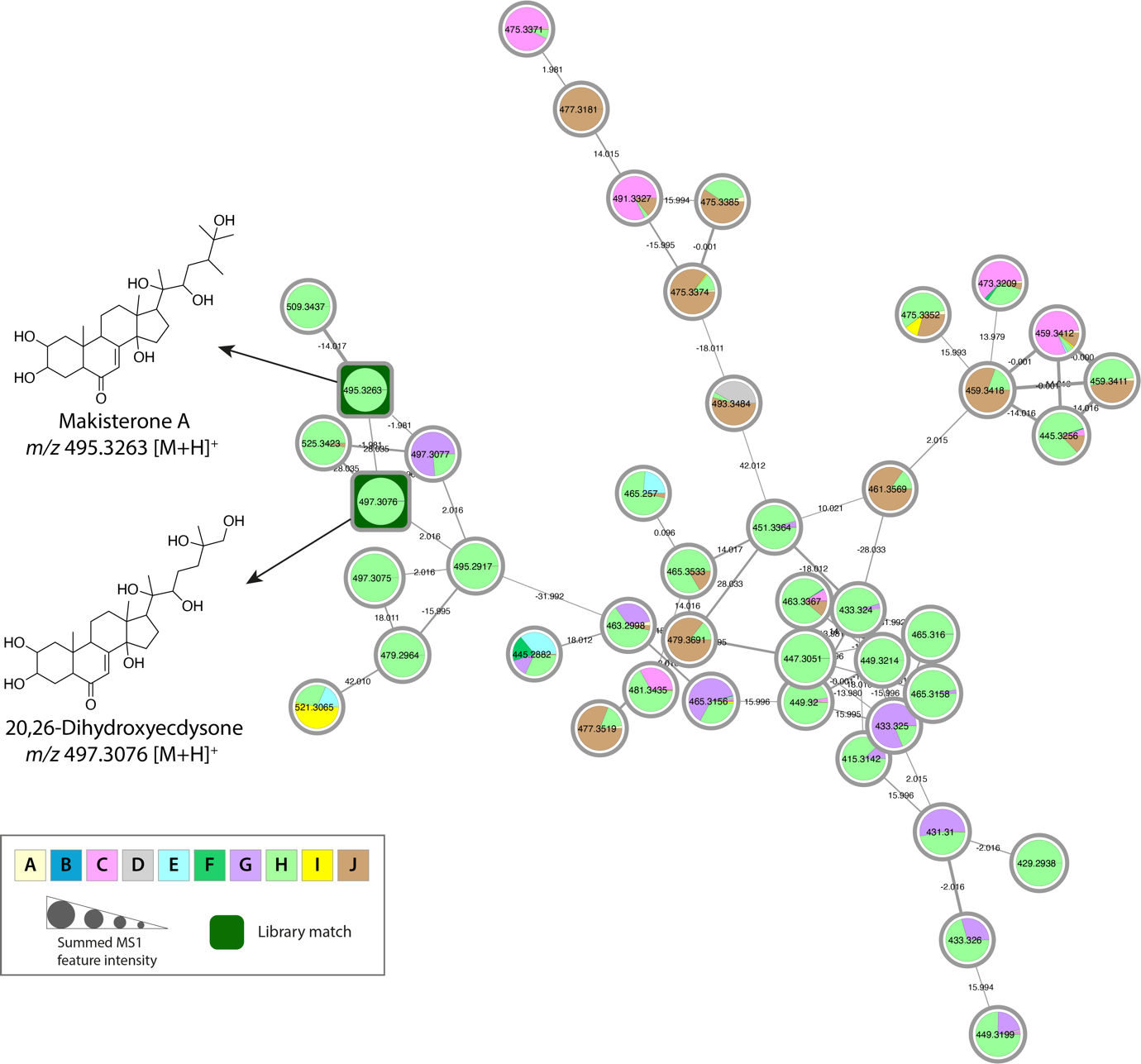


**Supplementary Figure 22.** Molecular family composed of ecdysteroids (positive ionization mode).


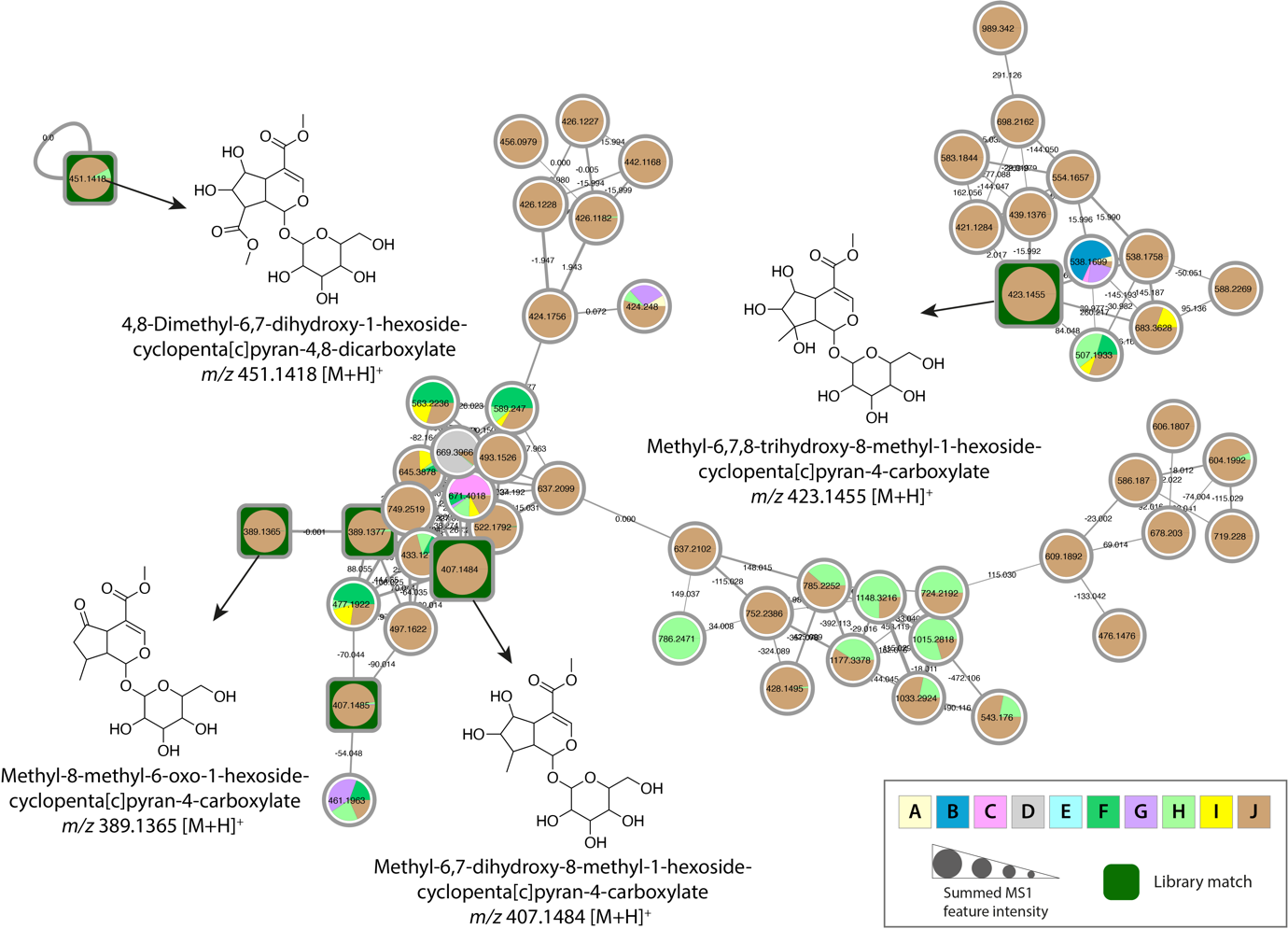


**Supplementary Figure 23.** Molecular families composed of iridoids (positive ionization mode).


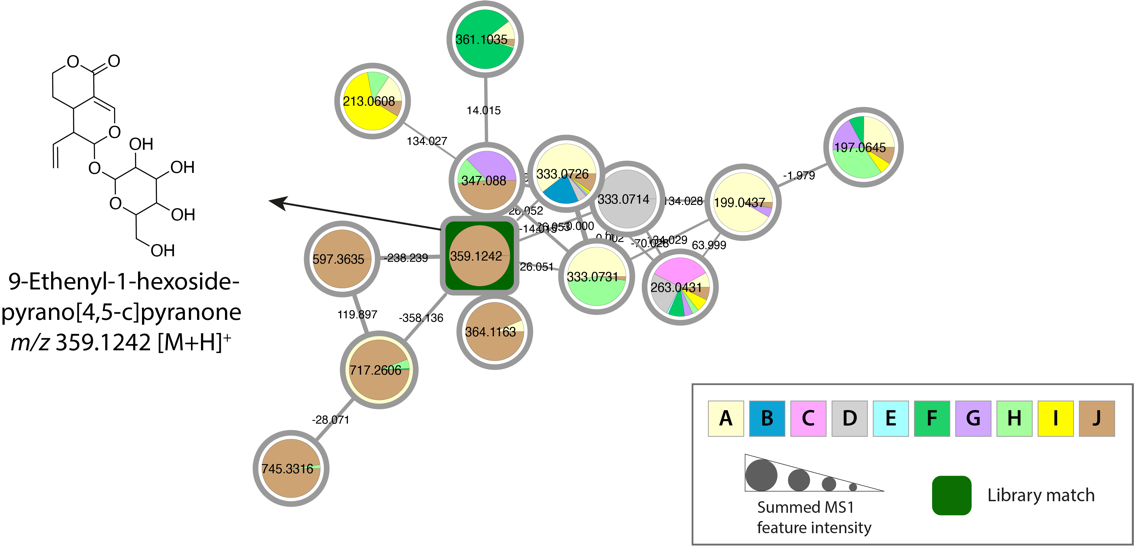


**Supplementary Figure 24.** Molecular family composed of secoiridoids (positive ionization mode).


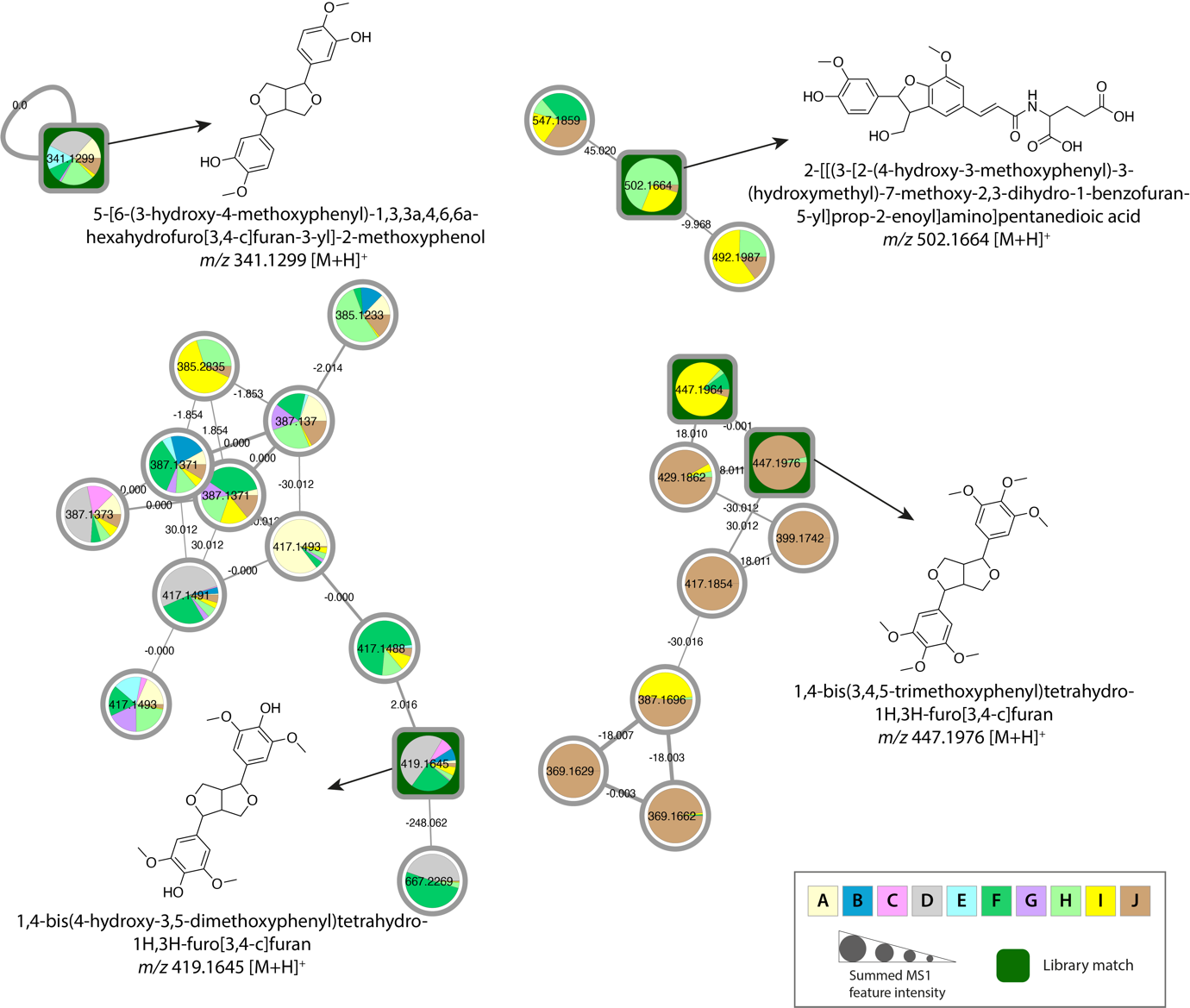


**Supplementary Figure 25.** Molecular families composed of neolignans and furofuranoid lignans (positive ionization mode).


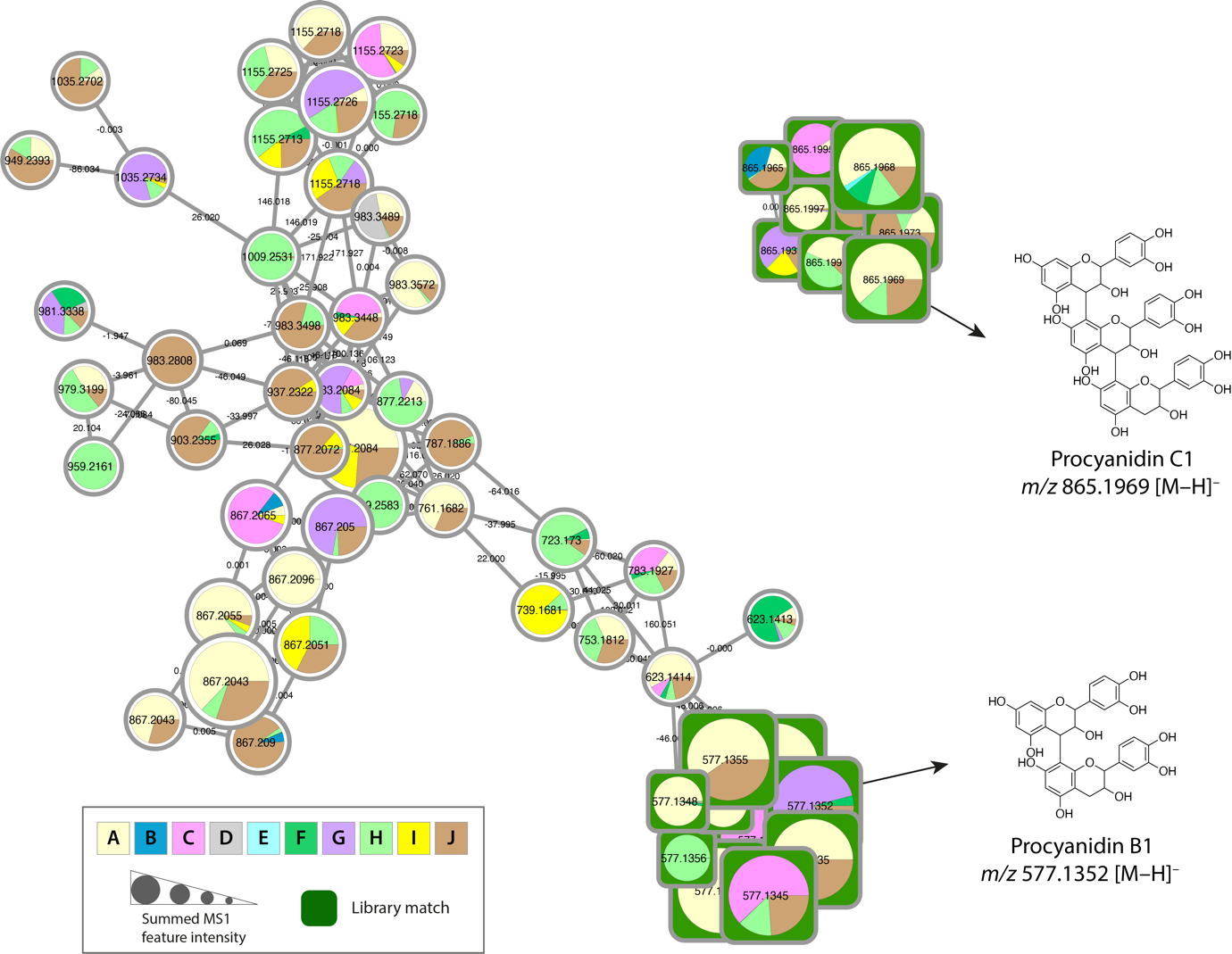


**Supplementary Figure 26.** Molecular families composed of condensed tannins (negative ionization mode).


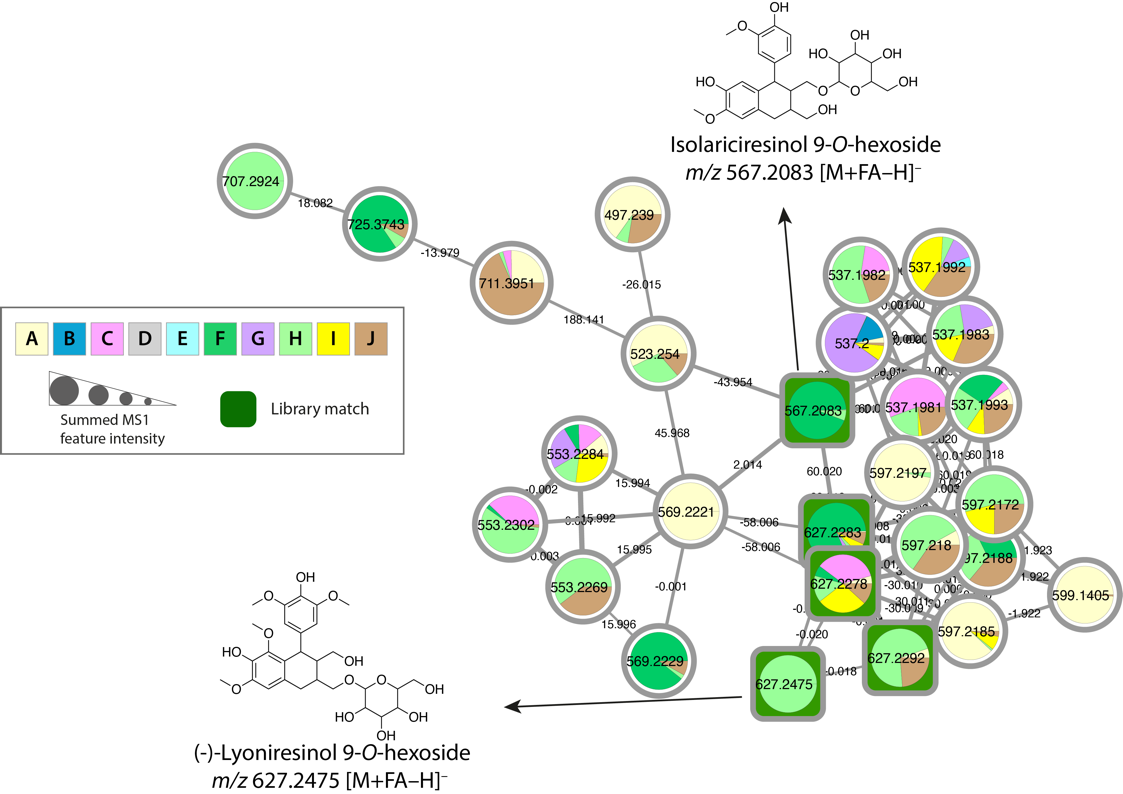


**Supplementary Figure 27.** Molecular families composed of lignans (negative ionization mode).


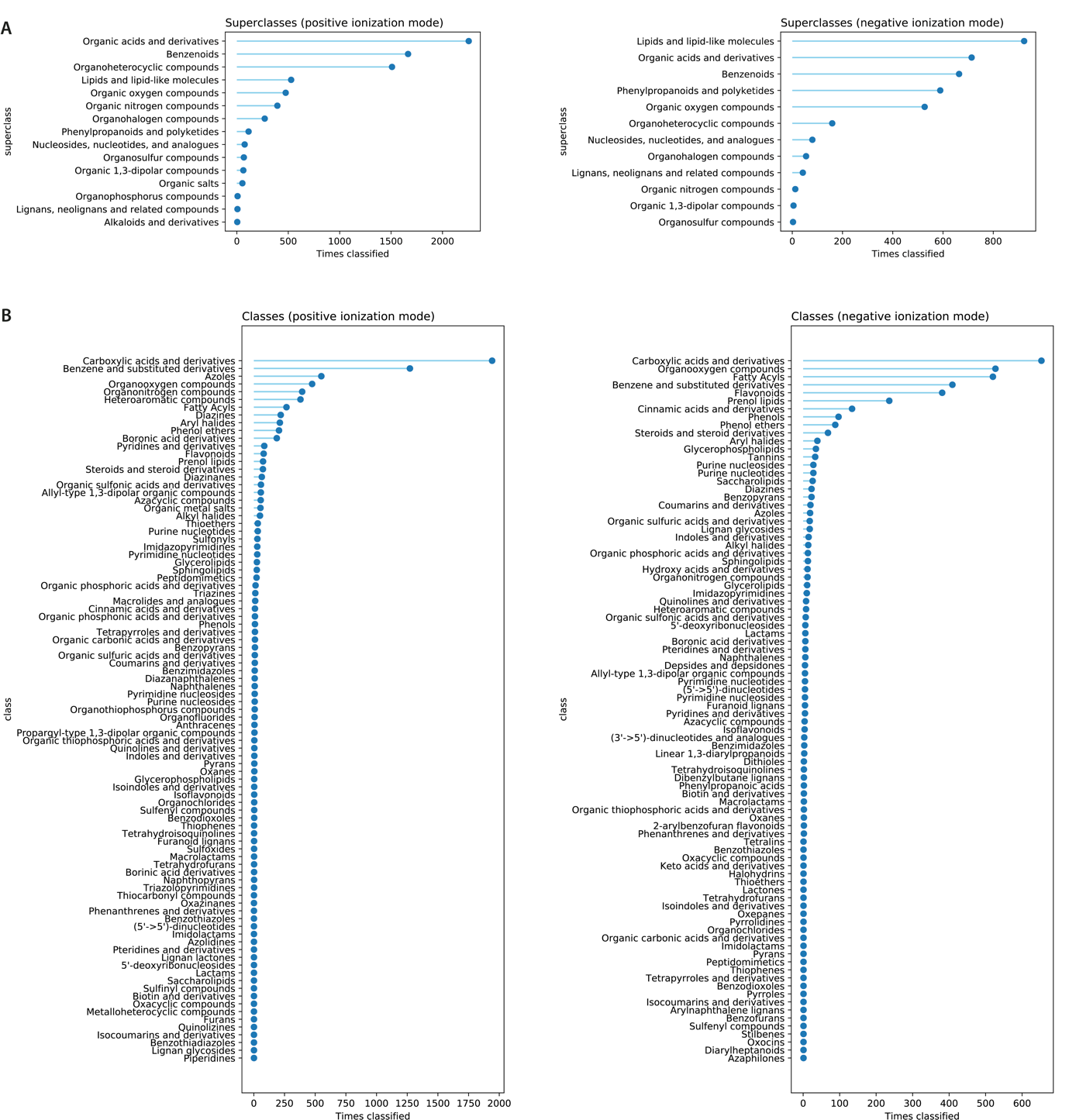


**Supplementary Figure 28.** Compound classification distribution within Malpighiaceae species in positive and negative ionization modes at a **(A)** CANOPUS superclass level and **(B)** CANOPUS class level.


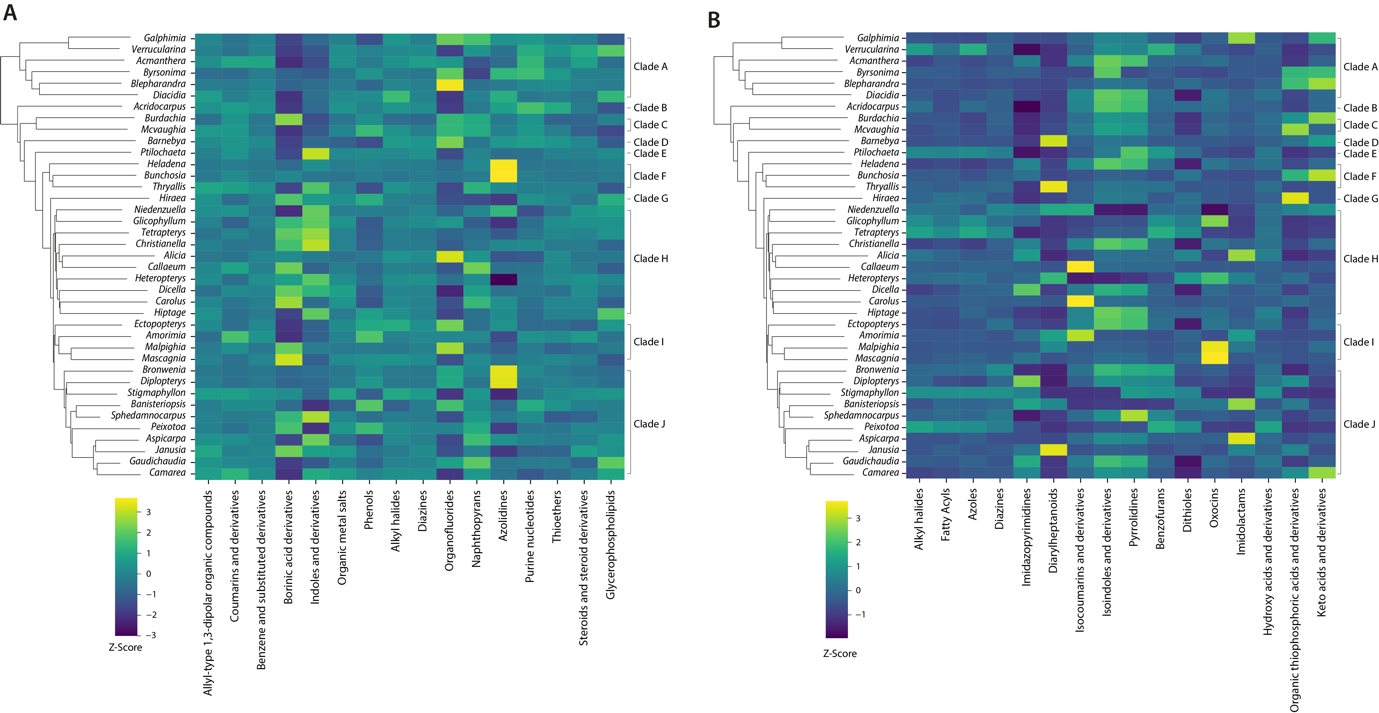


**Supplementary Figure 29.** Heatmap of the normalized ion features putatively annotated at a class level based on *in silico* classification using CANOPUS for the **(A)** positive and **(B)** negative ionization modes. The classes were filtered to keep only the classes with a high correlation with the most sampled clades. The cladogram on the left reflects the latest molecular phylogeny of the Malpighiaceae family.

**Supplementary Table 1.** Detailed information of the Malpighiaceae sampling: species list, collection site, location and date, biome, and phylogenetic group classification. Samples consisted of plant leaves, unless specified otherwise.

| **Sample** | **Genus^†^** | **Species** | **Collection code**  **(Herbarium)^‡^** | **Location and date (dd/mm/yyyy)** | **Collection site** | **Biome** | **Phylogenetic group** |
| --- | --- | --- | --- | --- | --- | --- | --- |
| **1** | *Acmanthera* | *latifolia* | Amorim 8575 (CEPEC) | Barcelos/AM; 15/04/2014 | 1 | Amazon rainforest | Byrsonimoid (A) |
| **2** | *Acmanthera* | *latifolia* | Amorim 8580 (CEPEC) | Barcelos/AM; 15/04/2014 | 1 | Amazon rainforest | Byrsonimoid (A) |
| **3** | *Acmanthera* | *latifolia* | Amorim 8649 (CEPEC) | Barcelos/AM; 25/04/2014 | 1 | Amazon rainforest | Byrsonimoid (A) |
| **4** | *Acridocarpus* | *smeathmannii* | Letouzey 11760 (US) | Djoum/Camarões; 01/01/1973 | 32 | African rainforest | Acridocarpoid (B) |
| **5** | *Alicia* | *anisopetala* | Rossetto 430 (HUEFS) | Londrina/PR; 15/07/2014 | 29 | Atlantic forest | Tetrapteroid (H) |
| **6** | *Alicia* | *anisopetala* | Pace 200 (SPF) | Corumbá/MS; 26/06/2012 | 20 | Pantanal | Tetrapteroid (H) |
| **7** | *Alicia* | *anisopetala* | Almeida 890 (RB) | Bauru/SP; 15/07/2018 | 28 | Atlantic forest/Cerrado | Tetrapteroid (H) |
| **8** | *Amorimia* | *andersonii* | Marinho 654 (CEPEC) | Itamaraju/BA; 16/02/2014 | 21 | Atlantic forest | Malpighioid (I) |
| **9** | *Amorimia* | *concinna* | Romero Castaneda 9252 (US) | Sincelejo/Colombia; 20/04/1963 | 3 | Amazon rainforest | Malpighioid (I) |
| **10** | *Amorimia* | *coriacea* | Almeida 615 (HUEFS) | Cabo Frio/RJ; 14/01/2016 | 24 | Atlantic forest | Malpighioid (I) |
| **11** | *Amorimia* | *pubiflora* | Moleta S/N (HUEFS) | Cuiabá/MT; 01/06/2011 | 16 | Cerrado | Malpighioid (I) |
| **12** | *Amorimia* | *septentrionalis* | Costa Lima 1163 (HUEFS) | Cachoeira dos índios/PB; 12/05/2014 | 8 | Caatinga | Malpighioid (I) |
| **13** | *Amorimia* | *septentrionalis* | Almeida 800 (HUEFS) | Maruim/SE; 11/11/2015 | 9 | Atlantic forest | Malpighioid (I) |
| **14** | *Aspicarpa* | *harleyi* | Almeida 874 (RB) | Caetité/BA; 02/11/2017 | 12 | Caatinga/Cerrado | Stigmaphylloid (J) |
| **15** | *Banisteriopsis* | *adenopoda* | Almeida 813 (HUEFS) | São Paulo/SP; 10/04/2012 | 25 | Atlantic forest | Stigmaphylloid (J) |
| **16** | *Banisteriopsis* | *adenopoda* | Almeida 813 (HUEFS) | São Paulo/SP; 10/04/2012 | 25 | Atlantic forest | Stigmaphylloid (J) |
| **17** | *Banisteriopsis* | *argyrophylla* | Almeida 808 (HUEFS) | Mogi Guaçu/SP; 12/08/2012 | 26 | Atlantic forest/Cerrado | Stigmaphylloid (J) |
| **18** | *Banisteriopsis* | *argyrophylla* | Almeida 811 (HUEFS) | Mogi Guaçu/SP; 12/08/2012 | 26 | Atlantic forestCerrado | Stigmaphylloid (J) |
| **19** | *Banisteriopsis* | *anisandra* | Francener 1138 (SP) | Pirenópolis/GO; 08/08/2012 | 14 | Cerrado | Stigmaphylloid (J) |
| **20** | *Banisteriopsis* | *anisandra* | Almeida 611 (HUEFS) | Rio de Contas/BA; 30/07/13 | 12 | Cerrado/Caatinga | Stigmaphylloid (J) |
| **21** | *Banisteriopsis* | *campestris* | Machado 20 (BHCB) | Serra do cipó/MG; 09/05/2018 | 23 | Cerrado | Stigmaphylloid (J) |
| **22** | *Banisteriopsis* | *harleyi* | Almeida 635 (HUEFS) | Piatã/BA; 10/12/2013 | 10 | Cerrado/Caatinga | Stigmaphylloid (J) |
| **23** | *Banisteriopsis* | *laevifolia* | Francener 1275 (SP) | Pedregulho/SP; 19/02/2013 | 27 | Cerrado | Stigmaphylloid (J) |
| **24** | *Banisteriopsis* | *laevifolia* | Francener 1359 (SP) | Vila Bela de Santíssima Trindade/MT; 21/03/2013 | 6 | Atlantic forest/Cerrado/Pantanal | Stigmaphylloid (J) |
| **25** | *Banisteriopsis* | *laevifolia* | RBv 3814 (RB) | Rio de Janeiro Botanical Garden; 21/09/2018 | 24 | Atlantic forest | Stigmaphylloid (J) |
| **26** | *Banisteriopsis* | *laevifolia* | Almeida 763 (HUEFS) | Aquidauana/MS; 26/01/2014 | 20 | Cerrado | Stigmaphylloid (J) |
| **27** | *Banisteriopsis* | *malifolia* | R1934 (IAC 54704) | Chapada do Araripe/CE; Jul/2013 | 7 | Caatinga | Stigmaphylloid (J) |
| **28** | *Banisteriopsis* | *malifolia* | Francener 1344 (SP) | Vilhena/RO; 13/03/2013 | 5 | Cerrado/Amazon rainforest | Stigmaphylloid (J) |
| **29** | *Banisteriopsis* | *malifolia* | Francener 1344 (SP) | Vilhena/RO; 13/03/2013 | 5 | Cerrado/Amazon rainforest | Stigmaphylloid (J) |
| **30** | *Banisteriopsis* | *malifolia* | Oliveira 25 (BHCB) | Serra do cipó/MG; 09/05/2018 | 23 | Cerrado | Stigmaphylloid (J) |
| **31** | *Banisteriopsis* | *megaphylla* | Francener 1131 (SP) | Brasília/DF; 07/08/2012 | 14 | Cerrado | Stigmaphylloid (J) |
| **32** | *Banisteriopsis* | *membranifolia* | Pellegrini 494 (RB) | Petrópolis/RJ; 04/04/2016 | 24 | Atlantic forest | Stigmaphylloid (J) |
| **33** | *Banisteriopsis* | *muricata* | Francener 1349 (SP) | Pimenta Bueno/RO; 20/03/2013 | 5 | Amazon rainforest | Stigmaphylloid (J) |
| **34** | *Banisteriopsis* | *muricata* | Almeida 545 (HUEFS) | Foz do Iguaçu/PR; 08/06/2013 | 30 | Atlantic forest | Stigmaphylloid (J) |
| **35** | *Banisteriopsis* | *parviglandula* | Costa-Lima 2180 (HUEFS) | Santa Teresa/ES; May/2015 | 22 | Atlantic forest | Stigmaphylloid (J) |
| **36** | *Banisteriopsis* | *quadrangulata* | Almeida 581 (HUEFS) | Jacobina/BA; 13/08/2013 | 10 | Caatinga | Stigmaphylloid (J) |
| **37** | *Banisteriopsis* | *stellaris* | R1935 (IAC 54705) | Chapada do Araripe/CE; Jul/2013 | 7 | Caatinga | Stigmaphylloid (J) |
| **38** | *Banisteriopsis* | *stellaris* | Francener 1123 (SP) | Nova Mutum/ MT; 02/05/2012 | 15 | Cerrado | Stigmaphylloid (J) |
| **39** | *Banisteriopsis* | *stellaris* | RBv 3821 (RB) | Rio de Janeiro Botanical Garden; 21/09/2018 | 24 | Atlantic forest | Stigmaphylloid (J) |
| **40** | *Banisteriopsis* | *stellaris* | Almeida 814 (HUEFS) | Mogi Guaçu/SP; 12/08/2012 | 26 | Atlantic forest/Cerrado | Stigmaphylloid (J) |
| **41** | *Banisteriopsis* | *variabilis* | Almeida 664 (HUEFS) | Chapadão do Céu/GO; 30/01/2014 | 17 | Cerrado | Stigmaphylloid (J) |
| **42** | *Banisteriopsis* | *variabilis* | Almeida 815 (HUEFS) | Mogi Guaçu/SP; 12/08/2012 | 26 | Atlantic forest/Cerrado | Stigmaphylloid (J) |
| **43** | *Banisteriopsis* | *vernonifolia* | Almeida 660 (HUEFS) | Alto Paraíso/GO; 26/01/2014 | 13 | Cerrado | Stigmaphylloid (J) |
| **44** | *Barnebya* | *harleyi* | Melo 1518 (HUEFS) | Itatim/BA; 30/10/2014 | 11 | Caatinga | Barnebyoid (D) |
| **45** | *Blepharandra* | *heteropetala* | Amorim 8608 (CEPEC) | Barcelos/AM; 16/04/2014 | 1 | Amazon rainforest | Byrsonimoid (A) |
| **46** | *Blepharandra* | *hypoleuca* | Amorim 8618 (CEPEC) | Barcelos/AM; 20/04/2014 | 1 | Amazon rainforest | Byrsonimoid (A) |
| **47** | *Bronwenia* | *megaptera* | Almeida 782 (HUEFS) | Itatim/BA; 20/10/2014 | 11 | Caatinga | Stigmaphylloid (J) |
| **48** | *Bunchosia* | *glandulirifera* | RBv 3789 (RB) | Rio de Janeiro Botanical Garden; 21/09/2018 | 24 | Atlantic forest | Bunchosioid (F) |
| **49** | *Bunchosia* | *montana* | Pace 418 (SPF) | Oaxaca/Mexico; 01/01/2013 | 32 | Seasonally dry forest | Bunchosioid (F) |
| **50** | *Bunchosia* | *pallescens* | RBv 3808 (RB) | Rio de Janeiro Botanical Garden; 21/09/2018 | 24 | Atlantic forest | Bunchosioid (F) |
| **51** | *Bunchosia* | *pallescens* | RBv 3808 (RB) | Rio de Janeiro Botanical Garden; 21/09/2018 | 24 | Atlantic forest | Bunchosioid (F) |
| **52** | *Burdachia* | *duckei* | Amorim 8647 (CEPEC) | Barcelos/AM; 25/04/2014 | 1 | Amazon rainforest | Mcvaughioid (C) |
| **53** | *Byrsonima* | *coccolobifolia* | M2 R1943 (IAC 55280) | Mogi Guaçu/SP; Jan/2014 | 26 | Atlantic forest/Cerrado | Byrsonimoid (A) |
| **54** | *Byrsonima* | *coccolobifolia* | M3 R1943 (IAC 55280) | Mogi Guaçu/SP; Jan/2014 | 26 | Atlantic forest/Cerrado | Byrsonimoid (A) |
| **55** | *Byrsonima* | *coccolobifolia* | M6 R1944 (IAC 55278) | Mogi Guaçu/SP; Jan/2014 | 26 | Atlantic forest/Cerrado | Byrsonimoid (A) |
| **56** | *Byrsonima* | *crassifolia* | Rapini 1553 (HUEFS) | Serra do cipó/MG; 09/05/2018 | 23 | Cerrado | Byrsonimoid (A) |
| **57** | *Byrsonima* | *incarnata* | Almeida 901 (RB) | Ananindeua/PA; 18/04/2015 | 4 | Amazon rainforest | Byrsonimoid (A) |
| **58** | *Byrsonima* | *intermedia* | M1 R1942 (IAC 55281) | Mogi Guaçu/SP; Jan/2014 | 26 | Atlantic forest/Cerrado | Byrsonimoid (A) |
| **59** | *Byrsonima* | *intermedia* | M4 R1942 (IAC 55281) | Mogi Guaçu/SP; Jan/2014 | 26 | Atlantic forest/Cerrado | Byrsonimoid (A) |
| **60** | *Byrsonima* | *ligustrifolia* | Almeida 805 (HUEFS) | Itanhaém/SP; 07/12/2015 | 25 | Atlantic forest | Byrsonimoid (A) |
| **61** | *Byrsonima* | *sericea* | R1933 (IAC 54703) | Chapada do Araripe/CE; Jul/2013 | 7 | Caatinga | Byrsonimoid (A) |
| **62** | *Byrsonima* | *sericea* | Trovó 656 (RB) | Rio de Janeiro/RJ; Abril/2015 | 24 | Atlantic forest | Byrsonimoid (A) |
| **63** | *Byrsonima* | *spicata* | Almeida 902 (RB) | Ananindeua/PA; 18/04/2015 | 4 | Amazon rainforest | Byrsonimoid (A) |
| **64** | *Byrsonima* | *verbascifolia* | M7 (IAC 55279) | Mogi Guaçu/SP; Jan/2014 | 26 | Atlantic forest/Cerrado | Byrsonimoid (A) |
| **65** | *Byrsonima* | *verbascifolia***^§^** | M7 (IAC 55279) | Mogi Guaçu/SP; Jan/2014 | 26 | Atlantic forest/Cerrado | Byrsonimoid (A) |
| **66** | *Callaeum* | *antifebrile* | Tamaio 110 (RB) | Rio de Janeiro Botanical Garden; 21/09/2018 | 24 | Atlantic forest | Tetrapteroid (H) |
| **67** | *Callaeum* | *psilophyllum* | Almeida 734 (HUEFS) | Feira de Santana/BA; 10/08/2013 | 11 | Caatinga | Tetrapteroid (H) |
| **68** | *Camarea* | *affinis X hirsuta* | Francener 1255 (SP) | Teresina de Goiás/GO; 10/01/2013 | 13 | Cerrado | Stigmaphylloid (J) |
| **69** | *Camarea* | *ericoides* | Lovo 453 (SPF) | Serra do Cipó/MG; 2013 | 23 | Cerrado | Stigmaphylloid (J) |
| **70** | *Carolus* | *chasei* | Almeida 585 (HUEFS) | Miguel Calmon/BA; 13/08/2013 | 10 | Caatinga | Tetrapteroid (H) |
| **71** | *Christianella* | *multiglandulosa* | Francener 1173 (SP) | Corguinho/MS; 19/10/2012 | 18 | Cerrado | Tetrapteroid (H) |
| **72** | *Diacidia* | *aracaensis* | Amorim 8617 (CEPEC) | Barcelos/AM; 20/04/2014 | 1 | Amazon rainforest | Byrsonimoid (A) |
| **73** | *Dicella* | *bracteosa* | Lombardi 1048 (BHCB) | Belo Horizonte/MG; 09/05/2018 | 23 | Atlantic forest | Tetrapteroid (H) |
| **74** | *Dicella* | *macroptera* | Francener 1254 (SP) | Taipas do Tocantins/TO; 14/01/2013 | 13 | Cerrado | Tetrapteroid (H) |
| **75** | *Diplopterys* | *pubipetala* | Francener 1126 (SP) | Nova Mutum/ MT; 02/05/2012 | 15 | Cerrado | Stigmaphylloid (J) |
| **76** | *Diplopterys* | *pubipetala* | Francener 1334 (SP) | União do Sul/MT; 16/03/2013 | 16 | Cerrado | Stigmaphylloid (J) |
| **77** | *Diplopterys* | *pubipetala* | Rando 1030 (BHCB) | Serra do cipó/MG; 09/05/2018 | 23 | Cerrado | Stigmaphylloid (J) |
| **78** | *Ectopopterys* | *soejartoi* | Wurdack 2356 (US) | Loreto/Peru; 21/10/1962 | 2 | Amazon rainforest | Malpighioid (I) |
| **79** | *Galphimia* | *brasiliensis* | Felix 372 (BHCB) | Belo Horizonte/MG; 09/05/2018 | 23 | Atlantic forest | Byrsonimoid (A) |
| **80** | *Gaudichaudia* | *albida* | Pace 419 (SPF) | Oaxaca/México; 21/08/2013 | 32 | Seasonally dry forest | Stigmaphylloid (J) |
| **81** | *Gaudichaudia* | *albida* | Pace 423 (SPF) | Veracruz/México; 01/01/2013 | 31 | Seasonally dry forest | Stigmaphylloid (J) |
| **82** | *Glicophyllum* | *ambiguum* | Francener 1165 (SP) | Araguainha/MT; 13/08/2012 | 14 | Cerrado/Caatinga | Tetrapteroid (H) |
| **83** | *Glicophyllum* | *cardiophyllum* | Almeida 641 (HUEFS) | Rio de Contas/BA; 09/12/2013 | 12 | Cerrado/Caatinga | Tetrapteroid (H) |
| **84** | *Glicophyllum* | *cardiophyllum* | Almeida 793 (HUEFS) | Rio de Contas/BA; 30/10/2014 | 12 | Cerrado/Caatinga | Tetrapteroid (H) |
| **85** | *Glicophyllum* | *microphyllum* | Francener 1264 (SP) | Alto Paraíso/GO; 15/01/2013 | 13 | Cerrado | Tetrapteroid (H) |
| **86** | *Glicophyllum* | *microphyllum* | Almeida 835b (HUEFS) | Serra do cipó/MG; 09/05/2018 | 23 | Cerrado | Tetrapteroid (H) |
| **87** | *Glicophyllum* | *ramiflorum* | Francener 1170 (SP) | Corguinho/MS; 19/10/2012 | 18 | Cerrado | Tetrapteroid (H) |
| **88** | *Heladena* | *multiflora* | Francener 1181 (SP) | Dourados/MS; 22/10/2012 | 19 | Atlantic forest/Cerrado | Bunchosioid (F) |
| **89** | *Heteropterys* | *aenea* | Almeida 798 (HUEFS) | Itapema/SC; 25/11/2015 | 31 | Atlantic forest | Tetrapteroid (H) |
| **90** | *Heteropterys* | *bicolor* | Barros 5309 (HUEFS) | Niterói/RJ; 11/11/2016 | 24 | Atlantic forest | Tetrapteroid (H) |
| **91** | *Heteropterys* | *brachiata* | Pace 406 (SPF) | Veracruz/Mexico; 01/01/2013 | 31 | Seasonally dry forest | Tetrapteroid (H) |
| **92** | *Heteropterys* | *brunnea* | Almeida 684 (HUEFS) | Jequitinhonha/MG; 10/12/2014 | 12 | Atlantic forest | Tetrapteroid (H) |
| **93** | *Heteropterys* | *byrsonimifolia* | Almeida 723 (HUEFS) | Seabra/BA; 15/11/2014 | 10 | Caatinga | Tetrapteroid (H) |
| **94** | *Heteropterys* | *campestris* | Almeida 657 (HUEFS) | Alto Paraíso/GO; 26/01/2014 | 13 | Cerrado | Tetrapteroid (H) |
| **95** | *Heteropterys* | *chrysophylla* | Trovó 658 (RB) | Rio de Janeiro/RJ; Apr/2015 | 24 | Atlantic forest | Tetrapteroid (H) |
| **96** | *Heteropterys* | *coleoptera* | RBv 8909 (RB) | Rio de Janeiro Botanical Garden; 21/09/2018 | 24 | Atlantic forest | Tetrapteroid (H) |
| **97** | *Heteropterys* | *coleoptera* | RBv 8909 (RB) | Rio de Janeiro Botanical Garden; 14/07/2018 | 24 | Atlantic forest | Tetrapteroid (H) |
| **98** | *Heteropterys* | *eglandulosa* | Francener 1141 (SP) | Pirenópolis/GO; 08/08/2012 | 14 | Cerrado | Tetrapteroid (H) |
| **99** | *Heteropterys* | *eglandulosa* | Almeida 726 (HUEFS) | Correntina/BA; 15/11/2014 | 12 | Cerrado | Tetrapteroid (H) |
| **100** | *Heteropterys* | *hatschbachii* | Francener 1184 (SP) | Rio Verde de Mato Grosso/MS; 24/10/2012 | 18 | Cerrado | Tetrapteroid (H) |
| **101** | *Heteropterys* | *intermedia* | Pellegrini 491 (RB) | Petrópolis/RJ; 22/09/2018 | 24 | Atlantic forest | Tetrapteroid (H) |
| **102** | *Heteropterys* | *intermedia* | Almeida 504 (SP) | Dores do Rio Preto/ES; 18/11/2011 | 24 | Atlantic forest | Tetrapteroid (H) |
| **103** | *Heteropterys* | *intermedia* | Almeida 505 (SP) | Dores do Rio Preto/ES; 18/11/2011 | 24 | Atlantic forest | Tetrapteroid (H) |
| **104** | *Heteropterys* | *leona* | Amorim 8651 (BHCB) | Barcelos/AM; 25/04/2014 | 1 | Amazon rainforest | Tetrapteroid (H) |
| **105** | *Heteropterys* | *leona* | Amorim 8577 (BHCB) | Barcelos/AM; 15/04/2014 | 1 | Amazon rainforest | Tetrapteroid (H) |
| **106** | *Heteropterys* | *leona* | Amorim 8597 (BHCB) | Barcelos/AM; 16/04/2014 | 1 | Amazon rainforest | Tetrapteroid (H) |
| **107** | *Heteropterys* | *leschinaultiana* | Trovó 660 (RB) | Itatiaia/RJ; Sep/2015 | 24 | Atlantic forest | Tetrapteroid (H) |
| **108** | *Heteropterys* | *leschinaultiana* | Trovó 668 (RB) | Nova Friburgo/RJ; Sep/2015 | 24 | Atlantic forest | Tetrapteroid (H) |
| **109** | *Heteropterys* | *oberdanii* | Almeida 521 (SP) | Sooretama/ES; 20/01/2012 | 22 | Atlantic forest | Tetrapteroid (H) |
| **110** | *Heteropterys* | *pauciflora* | Almeida 804 (HUEFS) | Vacaria/RS; 04/12/2015 | 31 | Atlantic forest | Tetrapteroid (H) |
| **111** | *Heteropterys* | *tomentosa* | Francener 1198 (SP) | Costa Rica/MS; 26/10/2012 | 17 | Cerrado | Tetrapteroid (H) |
| **112** | *Heteropterys* | *umbellata* | Almeida 838b (RB) | Serra do cipó/MG; 09/05/2018 | 23 | Cerrado | Tetrapteroid (H) |
| **113** | *Heteropterys* | *umbellata* | Almeida 838b (RB) | Serra do cipó/MG; 09/05/2018 | 23 | Cerrado | Tetrapteroid (H) |
| **114** | *Hiptage* | *benghalensis* | RBv 3801 (RB) | Rio de Janeiro Botanical Garden; 21/09/2018 | 24 | Atlantic forest | Tetrapteroid (H) |
| **115** | *Hiraea* | *cuiabensis* | Francener 1218 (SP) | Araguainha/MT; 29/12/2012 | 14 | Atlantic forest | Hiraeoid (G) |
| **116** | *Hiraea* | *hatschbachii* | Almeida 548 (HUEFS) | Foz do Iguaçu/PR; 08/06/2013 | 30 | Atlantic forest | Hiraeoid (G) |
| **117** | *Hiraea* | *reclinata* | Pace 518 (SPF) | Loreto/Peru; 18/09/2014 | 2 | Amazon rainforest | Hiraeoid (G) |
| **118** | *Hiraea* | *restingae* | Almeida 518 (SP) | Soretano/ES; 20/01/2012 | 22 | Atlantic forest | Hiraeoid (G) |
| **119** | *Hiraea* | *restingae* | Almeida 542 (SP) | Guarapari/ES; 30/01/2012 | 22 | Atlantic forest | Hiraeoid (G) |
| **120** | *Janusia* | *janusioides* | Francener 1117 (SP) | Nova Mutum/MT; 01/05/2004 | 15 | Amazon rainforest/Cerrado | Stigmaphylloid (J) |
| **121** | *Janusia* | *mediterranea* | RBv 4767 (RB) | Rio de Janeiro Botanical Garden; 21/09/2018 | 24 | Atlantic forest | Stigmaphylloid (J) |
| **122** | *Janusia* | *mediterranea* | Fontella 517 (RB) | Rio de Janeiro Botanical Garden, 11/08/2016 | 24 | Atlantic forest | Stigmaphylloid (J) |
| **123** | *Janusia* | *occhionii* | Francener 1361 (SP) | Vila Bela de Santíssima Trindade/MT; 21/03/2013 | 6 | Amazon rainforest/Cerrado/Pantanal | Stigmaphylloid (J) |
| **124** | *Janusia* | *schwannioides* | Almeida 578 (HUEFS) | Boa Nova/BA; 01/07/2013 | 12 | Atlantic forest | Stigmaphylloid (J) |
| **125** | *Malpighia* | *mexicana* | Pace 413 (SPF) | Oaxaca/Mexico; 21/08/2013 | 32 | Seasonally dry forest | Malpighioid (I) |
| **126** | *Malpighia* | *mexicana* | Pace 417 (SPF) | Oaxaca/Mexico; 01/01/2013 | 32 | Seasonally dry forest | Malpighioid (I) |
| **127** | *Mascagnia* | *conformis* | Amorim 8660 (CEPEC) | Barcelos/AM; 25/04/2014 | 1 | Amazon rainforest | Malpighioid (I) |
| **128** | *Mascagnia* | *sepium* | Almeida 822 (HUEFS) | Marilândia/ES; 10/10/2015 | 22 | Atlantic forest | Malpighioid (I) |
| **129** | *Mcvaughia* | *bahiana* | Guedes 12148 (ALCB) | Monte Santo/BA; 12/01/2006 | 10 | Atlantic forest/Caatinga | Mcvaughioid (C) |
| **130** | *Niedenzuella* | *acutifolia* | Barros 5136 (RB) | Cachoeiras de Macacu/RJ; 21/09/2018 | 24 | Atlantic forest | Tetrapteroid (H) |
| **131** | *Niedenzuella* | *acutifolia***^§^** | Barros 5136 (RB) | Cachoeiras de Macacu/RJ; 21/09/2018 | 24 | Atlantic forest | Tetrapteroid (H) |
| **132** | *Niedenzuella* | *acutifolia* | Almeida 519 (SP) | Sooretama/ES; 20/01/2012 | 22 | Atlantic forest | Tetrapteroid (H) |
| **133** | *Niedenzuella* | *lasiandra* | Francener 1182 (SP) | Rio verde de Mato Grosso/MS; 24/10/2012 | 18 | Cerrado | Tetrapteroid (H) |
| **134** | *Niedenzuella* | *lucida* | Almeida 626 (HUEFS) | Barro Preto/BA; 09/12/2013 | 21 | Atlantic forest | Tetrapteroid (H) |
| **135** | *Niedenzuella* | *multiglandulosa* | Francener 1199 (SP) | Costa Rica, MS; 26/10/2012 | 17 | Cerrado | Tetrapteroid (H) |
| **136** | *Niedenzuella* | *multiglandulosa* | HMS 5206 (CGMS) | Campo Grande, MS; 22/11/2015 | 18 | Cerrado | Tetrapteroid (H) |
| **137** | *Niedenzuella* | *multiglandulosa* | Almeida 523 (SP) | Sooretama/ES; 21/01/2012 | 22 | Atlantic forest | Tetrapteroid (H) |
| **138** | *Niedenzuella* | *multiglandulosa* | Almeida 639 (HUEFS) | Rio de Contas/BA; 09/12/2013 | 12 | Cerrado/Caatinga | Tetrapteroid (H) |
| **139** | *Niedenzuella* | *poeppigiana* | Pellegrini 490 (RB) | Petrópolis/RJ; 21/02/2016 | 24 | Atlantic forest | Tetrapteroid (H) |
| **140** | *Niedenzuella* | *poeppigiana* | Pellegrini 490 (RB) | Petrópolis/RJ; 20/09/2018 | 24 | Atlantic forest | Tetrapteroid (H) |
| **141** | *Niedenzuella* | *poeppigiana***^§^** | Pellegrini 490 (RB) | Petrópolis/RJ; 20/09/2018 | 24 | Atlantic forest | Tetrapteroid (H) |
| **142** | *Niedenzuella* | *poeppigiana* | Almeida 508 (SP) | Santa Teresa/ES; 22/11/2011 | 22 | Atlantic forest | Tetrapteroid (H) |
| **143** | *Niedenzuella* | *sericea* | Almeida 823 (HUEFS) | Rio de Contas/BA; 09/12/2013 | 12 | Cerrado/Caatinga | Tetrapteroid (H) |
| **144** | *Niedenzuella* | *stannea* | Francener 1233 (SP) | Lagoa da Confusão/TO; 12/01/2013 | 13 | Cerrado | Tetrapteroid (H) |
| **145** | *Peixotoa* | *cordistipula* | Almeida 666 (HUEFS) | Campo Grande/MS; 30/01/2014 | 18 | Cerrado | Stigmaphylloid (J) |
| **146** | *Peixotoa* | *glabra* | Almeida 843 (RB) | Milho Verde/MG; 01/11/2017 | 23 | Cerrado | Stigmaphylloid (J) |
| **147** | *Peixotoa* | *hispidula* | Almeida 510 (SP) | Guarapari/ES; 23/11/2011 | 22 | Atlantic forest | Stigmaphylloid (J) |
| **148** | *Peixotoa* | *reticulata* | Almeida 812 (HUEFS) | Mogi Guaçu/SP; 12/08/2012 | 26 | Atlantic forest/Cerrado | Stigmaphylloid (J) |
| **149** | *Peixotoa* | *spinensis* | Almeida 609 (HUEFS) | Mucugê/BA; 29/07/2013 | 10 | Cerrado/Caatinga | Stigmaphylloid (J) |
| **150** | *Peixotoa* | *tomentosa* | Almeida 833 (HUEFS) | Ouro Branco/MG; 30/10/2017 | 23 | Atlantic forest | Stigmaphylloid (J) |
| **151** | *Peixotoa* | *tomentosa* | Sebastiani 299 (BHCB) | Serra do cipó/MG; 09/05/2018 | 23 | Cerrado | Stigmaphylloid (J) |
| **152** | *Ptilochaeta* | *bahiensis* | Almeida 860 (RB) | Porteirinha/MG; 05/11/2017 | 12 | Cerrado/Caatinga | Ptilochaetoid (E) |
| **153** | *Ptilochaeta* | *densiflora* | Carvalho 290 (HUEFS) | Corumbá/MS; 07/04/2010 | 20 | Pantanal | Ptilochaetoid (E) |
| **154** | *Sphedamnocarpus* | *angolensis* | Humbert 15422 (US) | Matopo/Zimbabwe; 15/04/1934 | 33 | African savanna | Stigmaphylloid (J) |
| **155** | *Stigmaphyllon* | *acuminatum* | Almeida 821 (HUEFS) | Mimoso do Sul/ES; 20/10/2015 | 24 | Atlantic forest | Stigmaphylloid (J) |
| **156** | *Stigmaphyllon* | *alternifolium* | Almeida 511 (SP) | Divino de São Lourenço/ES; 17/11/2011 | 24 | Atlantic forest | Stigmaphylloid (J) |
| **157** | *Stigmaphyllon* | *angustilobum* | Almeida 503 (SP) | Divino de São Lourenço/ES; 17/11/2011 | 24 | Atlantic forest | Stigmaphylloid (J) |
| **158** | *Stigmaphyllon* | *auriculatum* | Almeida 584 (HUEFS) | Miguel Calmon/BA; 13/08/2013 | 10 | Caatinga | Stigmaphylloid (J) |
| **159** | *Stigmaphyllon* | *blanchetii* | Almeida 532 (SP) | Conceição da Barra/ES; 23/01/2012 | 22 | Atlantic forest | Stigmaphylloid (J) |
| **160** | *Stigmaphyllon* | *bonariense* | Queiroz 13530 (HUEFS) | Corrientes/Argentina; Dec/2007 | 30 | Atlantic forest | Stigmaphylloid (J) |
| **161** | *Stigmaphyllon* | *caatingicola* | Almeida 577 (HUEFS) | Rio de Contas/BA; 23/07/13 | 12 | Cerrado/Caatinga | Stigmaphylloid (J) |
| **162** | *Stigmaphyllon* | *cavernulosum* | Cardoso 2083 (HUEFS) | Rio de Contas/BA; Data | 12 | Cerrado/Caatinga | Stigmaphylloid (J) |
| **163** | *Stigmaphyllon* | *cavernulosum* | Gomes 1149 (HUEFS) | Salvador/BA; 04/06/2012 | 11 | Atlantic forest | Stigmaphylloid (J) |
| **164** | *Stigmaphyllon* | *ciliatum* | Almeida 541 (SP) | Guarapari/ES; 29/01/2012 | 22 | Atlantic forest | Stigmaphylloid (J) |
| **165** | *Stigmaphyllon* | *ciliatum* | Almeida 795 (HUEFS) | Itapema/SC; 25/11/2015 | 31 | Atlantic forest | Stigmaphylloid (J) |
| **166** | *Stigmaphyllon* | *convolvulifolium* | Francener 1330 (SP) | Carlinda/MT; 14/03/2013 | 15 | Amazon rainforest | Stigmaphylloid (J) |
| **167** | *Stigmaphyllon* | *gayanum* | Almeida 500 (SP) | Divino de São Lourenço/ES; 17/11/2011 | 24 | Atlantic forest | Stigmaphylloid (J) |
| **168** | *Stigmaphyllon* | *harleyi* | Santos 378 (HUEFS) | Morro do Chapéu/BA; 22/07/2005 | 10 | Caatinga | Stigmaphylloid (J) |
| **169** | *Stigmaphyllon* | *hatschbachii* | Almeida 903 (RB) | Wenceslau Guimarães/BA; 2015 | 11 | Atlantic forest | Stigmaphylloid (J) |
| **170** | *Stigmaphyllon* | *hatschbachii* | Sobrinho 10 (HUEFS) | Santa Teresinha/BA; 27/07/2000 | 11 | Atlantic forest/Caatinga | Stigmaphylloid (J) |
| **171** | *Stigmaphyllon* | *hispidum* | Marinho 854 (HUEFS) | Wenceslau Guimarães/BA; 2015 | 11 | Atlantic forest | Stigmaphylloid (J) |
| **172** | *Stigmaphyllon* | *lalandianum* | Almeida 816 (HUEFS) | Mogi Guaçu/SP; 12/08/2012 | 26 | Atlantic forest/Cerrado | Stigmaphylloid (J) |
| **173** | *Stigmaphyllon* | *lalandianum* | Almeida 840 (HUEFS) | Serra do cipó/MG; 09/05/2018 | 23 | Cerrado | Stigmaphylloid (J) |
| **174** | *Stigmaphyllon* | *macropodum* | Almeida 539 (SP) | Una/BA; 25/01/2012 | 21 | Atlantic forest | Stigmaphylloid (J) |
| **175** | *Stigmaphyllon* | *occidentale* | Melo 7226 (HUEFS) | Mateiros/TO; 11/11/2009 | 13 | Cerrado | Stigmaphylloid (J) |
| **176** | *Stigmaphyllon* | *palmatum* | Almeida 904 (RB) | Belém do Pará/PA; 15/04/2015 | 4 | Amazon rainforest | Stigmaphylloid (J) |
| **177** | *Stigmaphyllon* | *paralias* | R1928 (IAC 54701) | Chapada do Araripe/CE; Jul/2013 | 7 | Caatinga | Stigmaphylloid (J) |
| **178** | *Stigmaphyllon* | *paralias* | Almeida 820 (HUEFS) | Santa Teresa/ES; 15/10/2015 | 22 | Atlantic forest | Stigmaphylloid (J) |
| **179** | *Stigmaphyllon* | *puberulum* | Perdiz 732 (HUEFS) | Ilhéus/BA; 22/01/2010 | 21 | Atlantic forest | Stigmaphylloid (J) |
| **180** | *Stigmaphyllon* | *puberulum* | Almeida 905 (RB) | Wenceslau Guimarães/BA; 30/11/2014 | 11 | Atlantic forest | Stigmaphylloid (J) |
| **181** | *Stigmaphyllon* | *salzmannii* | Almeida 526 (SP) | São Mateus/ES; 21/01/2012 | 22 | Atlantic forest | Stigmaphylloid (J) |
| **182** | *Stigmaphyllon* | *saxicola* | Bardini S/N (HUEFS 198029) | Ponte Nova/MG; 21/05/1980 | 23 | Atlantic forest | Stigmaphylloid (J) |
| **183** | *Stigmaphyllon* | *saxicola* | Almeida 551 (HUEFS) | Catas Altas/MG; 22/07/2013 | 23 | Atlantic forest | Stigmaphylloid (J) |
| **184** | *Stigmaphyllon* | *saxicola* | Almeida 580 (HUEFS) | Boa Nova/BA; 01/07/2013 | 12 | Atlantic forest | Stigmaphylloid (J) |
| **185** | *Stigmaphyllon* | *sinuatum* | Amorim 8643 (CEPEC) | Barcelos/AM; 25/04/2014 | 1 | Amazon rainforest | Stigmaphylloid (J) |
| **186** | *Stigmaphyllon* | *tomentosum* | Almeida 679 (HUEFS) | Jequitinhonha/MG; 10/12/2014 | 12 | Atlantic forest | Stigmaphylloid (J) |
| **187** | *Stigmaphyllon* | *urenifolium* | Guedes 13932 (HUEFS) | Bom Jesus da Lapa/BA; 22/09/2007 | 10 | Caatinga/Cerrado | Stigmaphylloid (J) |
| **188** | *Stigmaphyllon* | *urenifolium* | Almeida 728 (HUEFS) | Correntina/BA; 15/11/2014 | 12 | Cerrado | Stigmaphylloid (J) |
| **189** | *Stigmaphyllon* | *vitifolium* | RBv 3820 (RB) | Rio de Janeiro Botanical Garden; 21/09/2018 | 24 | Atlantic forest | Stigmaphylloid (J) |
| **190** | *Tetrapterys* | *mucronata* | Almeida 517 (SP) | Santa Teresa/ES; 17/01/2012 | 22 | Atlantic forest | Tetrapteroid (H) |
| **191** | *Tetrapterys* | *phlomoides* | Moraes 40 (RB) | Petrópolis/RJ; 20/09/2018 | 24 | Atlantic forest | Tetrapteroid (H) |
| **192** | *Tetrapterys* | *phlomoides***^§^** | Moraes 40 (RB) | Petrópolis/RJ; 20/09/2018 | 24 | Atlantic forest | Tetrapteroid (H) |
| **193** | *Tetrapterys* | *schiedeana* | Pace 402 (SPF) | Veracruz/Mexico; 11/08/2013 | 31 | Seasonally dry forest | Tetrapteroid (H) |
| **194** | *Tetrapterys* | *xylosteifolia* | Almeida 800 (HUEFS) | Itapema/SC; 25/11/2015 | 31 | Atlantic forest | Tetrapteroid (H) |
| **195** | *Thryallis* | *latifolia* | Lombardi 685 (BHCB) | Belo Horizonte/MG; 09/05/2018 | 23 | Atlantic forest | Bunchosioid (F) |
| **196** | *Verrucularina* | *glaucophylla* | Roque 4265 (HUEFS) | Palmeiras/BA; 24/05/2014 | 10 | Cerrado/Caatinga | Byrsonimoid (A) |
| **197** | *Verrucularina* | *piresii* | Amorim 8619 (CEPEC) | Barcelos/AM; 20/04/2014 | 1 | Amazon rainforest | Byrsonimoid (A) |

**^†^**It is important to emphasize that very recently, some species have been synonymized: *Aenigmatanthera lasiandra* to *Niedenzuella lasiandra*; *Tetrapterys ambigua* to *Glicophyllum ambiguum*; *Tetrapterys cardiophylla* to *Glicophyllum cardiophyllum*; *Tetrapterys microphylla* to *Glicophyllum microphyllum*; *Tetrapterys ramiflora* to *Glicophyllum ramiflorum*.

**^‡^ALCB:** Herbarium Alexandre Leal Costa – Federal University of Bahia; **BHCB:** Herbarium of the Federal University of Minas Gerais; **CEPEC:** Herbarium André Maurício V. de Carvalho; **CGMS:** Herbarium of Federal University of Mato Grosso do Sul; **HUEFS:** Herbarium of State University of Feira de Santana; **IAC:** Agronomic Institute of Campinas; **RB:** Rio de Janeiro Botanical Garden Herbarium; **RBv:** Arboretum of Rio de Janeiro Botanical Garden (living collection); **SP:** Institute of Botany of São Paulo; **SPF:** Herbarium of the University of São Paulo; **US:** Smithsonian Institution United States National Herbarium.

**^§^**Bark.

**Supplementary Table 2.** Characters retrieved from the ancestral characters reconstruction (all clades and genera) based on the classifications obtained *in silico* for Malpighiaceae samples. The ionization mode in which each classification was obtained is described (pos = positive ionization mode; neg = negative ionization mode; both = both ionization modes).

|  | **Classes present^†^** | **Classes absent** |
| --- | --- | --- |
| **Clade A (Byrsonimoid clade)** | Macrolactams (both); Sulfenyl compounds (pos) | – |
| Tribe Galphimieae (*Galphimia*+*Verrucularina*) | Naphthopyrans (pos); Oxazinanes (pos) | – |
| *Galphimia* | (3'→5')-dinucleotides and analogues (neg); Imidolactams (both); Keto acids and derivatives (neg); Metalloheterocyclic compounds (pos); Organofluorides (pos); Oxacyclic compounds (both) | Benzopyrans (both); Lactams (both); Lignan glycosides (both); Organochlorides (both); Peptidomimetics (both)**^§^**, Pyrans (both); Pyrimidine nucleosides (both); Tetrahydrofurans (both); Thiocarbonyl compounds (pos) |
| *Verrucularina* | Benzofurans (neg); Biotin and derivatives (both); Diazanaphthalenes (pos); Dithioles (neg); Furanoid lignans (both); Hydroxy acids and derivatives (neg); Linear 1,3-diarylpropanoids (neg) | Naphthalenes (both) |
| Tribe Acmanthereae (*Acmanthera*) | Diazanaphthalenes (pos); Propargyl-type 1,3-dipolar organic compounds (pos) | Benzopyrans (both); Lactams (both); Lignan glycosides (both); Pyrimidine nucleosides (both); Pyrimidine nucleotides (both)**^§^**, Saccharolipids (both)**^§^**, Sulfenyl compounds (pos) |
| Acmanthereae+Byrsonimeae clade | – | (5'→5')-dinucleotides (both); Organic phosphonic acids and derivatives (pos); Pteridines and derivatives (both) |
| Tribe Byrsonimeae | Hydroxy acids and derivatives (neg); Imidolactams (both); Keto acids and derivatives (neg); Organic phosphoric acids and derivatives (both); Organofluorides (pos) | Oxanes (both) |
| *Byrsonima* | Furanoid lignans (both) | Macrolactams (both); Organochlorides (both) |
| *Blepharandra*+*Diacidia* clade | Anthracenes (pos); Pteridines and derivatives (both) | Thiocarbonyl compounds (pos) |
| *Blepharandra* | (5'→5')-dinucleotides (both); Benzothiadiazoles (pos); Diazanaphthalenes (pos); Dithioles (neg); Oxacyclic compounds (both); Oxanes (both); Oxazinanes (pos); Phenanthrenes and derivatives (both); Sulfoxides (pos); Tetralins (neg); Triazolopyrimidines (pos)**^§^** | – |
| *Diacidia* | Depsides and depsidones (neg); Isoflavonoids (both); Piperidines (pos); Quinolizines (pos); Tetrahydroisoquinolines (both) | Benzimidazoles (both); Coumarins and derivatives (both); Imidolactams (both); Keto acids and derivatives (neg); Lignan glycosides (both); Organofluorides (pos); Organothiophosphorus compounds (pos); Pyrans (both); Pyrimidine nucleosides (both); Sulfenyl compounds (pos); Tetrapyrroles and derivatives (both) |
| **Clade B (Acridocarpoid clade)** | Diazanaphthalenes (pos); Isoflavonoids (both); Oxacyclic compounds (both) | Tetrahydrofurans (both) |
| Clade B+remaining clades | Dithioles (neg); Furanoid lignans (both); Organic phosphoric acids and derivatives (both); Propargyl-type 1,3-dipolar organic compounds (pos) | – |
| **Clade C (Mcvaughioid clade)** | Linear 1,3-diarylpropanoids (neg) | Dithioles (neg); Indoles and derivatives (both) |
| *Burdachia* | Borinic acid derivatives (pos); Isocoumarins and derivatives (both); Keto acids and derivatives (neg); Organochlorides (both); Oxacyclic compounds (both); Oxazinanes (pos); Thiocarbonyl compounds (pos) | (5'→5')-dinucleotides (both); Anthracenes (pos); Benzopyrans (both); Propargyl-type 1,3-dipolar organic compounds (pos); Thiophenes (both) |
| *Mcvaughia* | Biotin and derivatives (both); Isoflavonoids (both); Sulfinyl compounds (pos) | Benzimidazoles (both); Furanoid lignans (both); Organic carbonic acids and derivatives (both); Organic phosphonic acids and derivatives (pos); Pyrimidine nucleosides (both) |
| Clade C+remaining clades | Anthracenes (pos); Hydroxy acids and derivatives (neg); Naphthopyrans (pos); Organofluorides (pos); Thiophenes (both)**^§^** | Organochlorides (both); Pyrans (both); Thiocarbonyl compounds (pos) |
| **Clade D (Barnebyoid clade)** | Diarylheptanoids (neg); Keto acids and derivatives (neg); Oxazinanes (pos) | Benzopyrans (both); Furanoid lignans (both); Glycerophospholipids (both)**^§^**, Lignan glycosides (both); Naphthalenes (both); Naphthopyrans (pos); Propargyl-type 1,3-dipolar organic compounds (pos); Pteridines and derivatives (both); Tetrahydrofurans (both) |
| Clade D+remaining clades | Metalloheterocyclic compounds (pos) | Oxanes (both) |
| **Clade E (Ptilochaetoid clade)** | 2-arylbenzofuran flavonoids (neg); Dibenzylbutane lignans (neg); Isoflavonoids (both); Oxacyclic compounds (both); Oxanes (both); Pyrrolidines (neg); Thiocarbonyl compounds (pos) | Organothiophosphorus compounds (pos); Thiophenes (both) |
| Clade E+remaining clades | Benzofurans (neg); Biotin and derivatives (both); Macrolactams (both); Pyrans (both); Sulfenyl compounds (pos) | Organic thiophosphoric acids and derivatives (both); Organofluorides (pos) |
| **Clade F (Bunchosioid clade)** | Azolidines (pos); Benzodioxoles (both); Organochlorides (both); Quinolizines (pos) | Organic carbonic acids and derivatives (both); Organic phosphoric acids and derivatives (both) |
| *Heladena* | (3'→5')-dinucleotides and analogues (neg); 5'-deoxyribonucleosides (both); Halohydrins (neg) | Benzofurans (neg); Benzopyrans (both); Dithioles (neg); Furanoid lignans (both); Organic sulfuric acids and derivatives (both); Propargyl-type 1,3-dipolar organic compounds (pos); Pteridines and derivatives (both); Tetrahydrofurans (both) |
| *Bunchosia* | Lactams (both); Linear 1,3-diarylpropanoids (neg); Organic thiophosphoric acids and derivatives (both); Organofluorides (pos); Phenylpropanoic acids (neg); Sulfoxides (pos) | Benzodioxoles (both); Biotin and derivatives (both); Organochlorides (both); Pyrans (both); Quinolizines (pos); Tetrapyrroles and derivatives (both) |
| *Bunchosia*+*Thryallis* clade | Thiocarbonyl compounds (pos) | Sulfenyl compounds (pos); Thiophenes (both) |
| *Thryallis* | Diarylheptanoids (neg); Dibenzylbutane lignans (neg); Furans (pos)**^§^**, Lactones (neg)**^§^**, Metalloheterocyclic compounds (pos); Organic carbonic acids and derivatives (both); Organic phosphoric acids and derivatives (both); Oxanes (both); Sulfinyl compounds (pos) | Anthracenes (pos); Azolidines (pos); Naphthalenes (both); Pyrimidine nucleosides (both) |
| Clade F+remaining clades | Diazanaphthalenes (pos) | (5'→5')-dinucleotides (both); Lactams (both); Metalloheterocyclic compounds (pos) |
| **Clade G (Hiraeoid clade)** | Piperidines (pos) | Benzofurans (neg); Benzopyrans (both); Dithioles (neg); Furanoid lignans (both); Hydroxy acids and derivatives (neg); Naphthopyrans (pos); Pteridines and derivatives (both); Pyrimidine nucleosides (both) |
| Clade G+remaining clades | Borinic acid derivatives (pos); Organic thiophosphoric acids and derivatives (both) | Anthracenes (pos); Macrolactams (both) |
| **Clade H (Tetrapteroid clade)** | – | Organic phosphonic acids and derivatives (pos) |
| *Glicophyllum* | Arylnaphthalene lignans (neg); Lactams (both); Linear 1,3-diarylpropanoids (neg); Macrolactams (both); Organofluorides (pos) | Naphthopyrans (pos); Tetrahydrofurans (both); Thiophenes (both) |
| *Glicophyllum*+*Niedenzuella* clade | Organochlorides (both) | Sulfenyl compounds (pos) |
| *Niedenzuella* | Anthracenes (pos); Thiocarbonyl compounds (pos) | Borinic acid derivatives (pos); Organic thiophosphoric acids and derivatives (both); Oxanes (both); Pyrimidine nucleosides (both) |
| *Tetrapterys* | (5'→5')-dinucleotides (both); Benzothiadiazoles (pos); Depsides and depsidones (neg); Linear 1,3-diarylpropanoids (neg); Macrolactams (both); Phenylpropanoic acids (neg); Tetralins (neg) | Metalloheterocyclic compounds (pos); Naphthopyrans (pos); Organic thiophosphoric acids and derivatives (both); Propargyl-type 1,3-dipolar organic compounds (pos); Thiophenes (both) |
| Remaining clades | – | Benzofurans (neg); Benzopyrans (both); Diazanaphthalenes (pos); Furanoid lignans (both); Lignan glycosides (both); Pyrimidine nucleosides (both) |
| *Christianella* | Isoindoles and derivatives (both); Oxacyclic compounds (both); Tetrahydroisoquinolines (both) | Coumarins and derivatives (both); Dithioles (neg); Naphthopyrans (pos); Organothiophosphorus compounds (pos); Pteridines and derivatives (both); Pyrans (both); Tannins (neg)**^§^**, Tetrahydrofurans (both) |
| *Christianella*+remaining clades | Lactams (both); Organochlorides (both) | Sulfenyl compounds (pos) |
| *Alicia* | Anthracenes (pos); Benzothiadiazoles (pos); Benzothiazoles (both); Diazanaphthalenes (pos); Imidolactams (both); Organofluorides (pos); Thiocarbonyl compounds (pos) | Biotin and derivatives (both); Borinic acid derivatives (pos); Lactams (both); Organic thiophosphoric acids and derivatives (both); Organochlorides (both); Oxanes (both); Thiophenes (both) |
| *Alicia*+*Callaeum* clade | Arylnaphthalene lignans (neg); Macrolactams (both); Organic phosphonic acids and derivatives (pos) | Metalloheterocyclic compounds (pos) |
| *Callaeum* | 5'-deoxyribonucleosides (both); Benzodioxoles (both); Benzofurans (neg); Benzopyrans (both); Depsides and depsidones (neg); Dibenzylbutane lignans (neg); Isocoumarins and derivatives (both); Lignan glycosides (both); Lignan lactones (pos); Phenylpropanoic acids (neg); Piperidines (pos); Sulfenyl compounds (pos) | – |
| *Heteropterys* | Lignan glycosides (both); Pyrimidine nucleosides (both) | Borinic acid derivatives (pos); Propargyl-type 1,3-dipolar organic compounds (pos) |
| *Heteropterys*+remaining clades | – | – |
| *Dicella* | Depsides and depsidones (neg); Furanoid lignans (both); Isoflavonoids (both); Oxacyclic compounds (both); Sulfoxides (pos) | – |
| *Dicella*+remaining clades | (5'→5')-dinucleotides (both); Benzopyrans (both); Quinolizines (pos); Tetrahydroisoquinolines (both) | Dithioles (neg); Oxanes (both) |
| *Carolus* | Isocoumarins and derivatives (both); Organochlorides (both); Oxanes (both); Phenylpropanoic acids (neg) | (5'→5')-dinucleotides (both); Coumarins and derivatives (both); Hydroxy acids and derivatives (neg); Indoles and derivatives (both); Metalloheterocyclic compounds (pos); Organic carbonic acids and derivatives (both); Organothiophosphorus compounds (pos); Pyrans (both); Sulfenyl compounds (pos); Tetrahydrofurans (both); Tetrapyrroles and derivatives (both); Thiophenes (both); Triazines (pos)**^§^** |
| *Carolus*+*Hiptage* clade | 5'-deoxyribonucleosides (both) | – |
| *Hiptage* | Diazanaphthalenes (pos); Dithioles (neg) | Benzopyrans (both); Borinic acid derivatives (pos); Naphthopyrans (pos); Organic thiophosphoric acids and derivatives (both); Propargyl-type 1,3-dipolar organic compounds (pos); Pteridines and derivatives (both); Quinolines and derivatives (both)**^§^**, Quinolizines (pos) |
| Clade H+remaining clades | Metalloheterocyclic compounds (pos); Oxanes (both) | – |
| **Clade I (Malpighioid clade)** | (3'→5')-dinucleotides and analogues (neg); Imidolactams (both); Piperidines (pos) | Benzofurans (neg); Furanoid lignans (both); Lignan glycosides (both) |
| *Ectopopterys* | Organofluorides (pos); Sulfoxides (pos) | Benzimidazoles (both); Coumarins and derivatives (both); Dithioles (neg); Naphthopyrans (pos); Organic phosphonic acids and derivatives (pos); Organic sulfuric acids and derivatives (both); Organic thiophosphoric acids and derivatives (both); Pyrans (both); Thiocarbonyl compounds (pos) |
| *Amorimia* | – | Biotin and derivatives (both); Hydroxy acids and derivatives (neg); Metalloheterocyclic compounds (pos); Piperidines (pos); Pyrimidine nucleosides (both); Thiophenes (both) |
| *Amorimia*+remaining clades | – | Diazanaphthalenes (pos) |
| *Malpighia* | Anthracenes (pos); Diarylheptanoids (neg); Keto acids and derivatives (neg); Lignan glycosides (both); Linear 1,3-diarylpropanoids (neg); Organofluorides (pos); Quinolizines (pos); Sulfinyl compounds (pos) | Propargyl-type 1,3-dipolar organic compounds (pos); Sulfenyl compounds (pos) |
| *Malpighia*+*Mascagnia* clade | Borinic acid derivatives (pos); Isoflavonoids (both); Oxocins (neg)**^§^** | (3'→5')-dinucleotides and analogues (neg); Benzopyrans (both) |
| *Mascagnia* | (5'→5')-dinucleotides (both); Benzodioxoles (both); Depsides and depsidones (neg); Isoindoles and derivatives (both); Lactams (both); Lignan lactones (pos); Macrolactams (both); Oxacyclic compounds (both); Phenanthrenes and derivatives (both) | Imidolactams (both) |
| Clades I+J | Thiocarbonyl compounds (pos) | Borinic acid derivatives (pos) |
| **Clade J (Stigmaphylloid clade)** | Macrolactams (both) | Biotin and derivatives (both); Sulfenyl compounds (pos) |
| *Bronwenia* | Anthracenes (pos); Azolidines (pos); Lactams (both); Linear 1,3-diarylpropanoids (neg); Organofluorides (pos); Phenylpropanoic acids (neg); Sulfoxides (pos) | Metalloheterocyclic compounds (pos); Organic carbonic acids and derivatives (both); Organic phosphoric acids and derivatives (both); Oxanes (both); Pyrans (both); Tetrapyrroles and derivatives (both); Thiophenes (both) |
| *Diplopterys* | (5'→5')-dinucleotides (both); 5'-deoxyribonucleosides (both); Azolidines (pos); Depsides and depsidones (neg); Imidolactams (both); Organofluorides (pos); Sulfenyl compounds (pos) | Benzofurans (neg); Dithioles (neg); Furanoid lignans (both); Indoles and derivatives (both); Naphthalenes (both); Naphthopyrans (pos); Organic phosphonic acids and derivatives (pos); Organochlorides (both); Oxanes (both); Propargyl-type 1,3-dipolar organic compounds (pos) |
| *Stigmaphyllon* | Biotin and derivatives (both); Pyrimidine nucleosides (both) | Macrolactams (both) |
| *Diplopterys*+*Stigmaphyllon*+remaining genera | Organochlorides (both) | Pyrimidine nucleosides (both) |
| *Banisteriopsis* | Diazanaphthalenes (pos) | Benzofurans (neg); Benzopyrans (both); Dithioles (neg); Furanoid lignans (both); Macrolactams (both); Metalloheterocyclic compounds (pos); Tetrahydrofurans (both) |
| *Sphedamnocarpus* | 2-arylbenzofuran flavonoids (neg); 5'-deoxyribonucleosides (both); Anthracenes (pos); Arylnaphthalene lignans (neg); Biotin and derivatives (both); Borinic acid derivatives (pos); Depsides and depsidones (neg); Isoflavonoids (both); Lactams (both); Linear 1,3-diarylpropanoids (neg); Oxacyclic compounds (both); Pyrimidine nucleosides (both); Pyrrolidines (neg); Sulfenyl compounds (pos) | Thiophenes (both) |
| *Banisteriopsis*+*Sphedamnocarpus*+remaining clades | – | Diazanaphthalenes (pos) |
| *Peixotoa* | Dibenzylbutane lignans (neg); Linear 1,3-diarylpropanoids (neg) | Organic carbonic acids and derivatives (both); Organic phosphonic acids and derivatives (pos); Pyrans (both); Thiophenes (both) |
| *Peixotoa*+remaining clades | – | Thiocarbonyl compounds (pos) |
| *Aspicarpa* | Depsides and depsidones (neg); Diazanaphthalenes (pos); Dithioles (neg); Imidolactams (both); Phenanthrenes and derivatives (both); Thiocarbonyl compounds (pos) | Biotin and derivatives (both); Lignan glycosides (both); Organic phosphoric acids and derivatives (both); Piperidines (pos); Sulfenyl compounds (pos); Tetrapyrroles and derivatives (both) |
| *Aspicarpa*+*Janusia* clade | Lactams (both) | Macrolactams (both) |
| *Janusia* | Metalloheterocyclic compounds (pos) | Naphthopyrans (pos); Organic carbonic acids and derivatives (both); Thiophenes (both) |
| *Aspicarpa*+*Janusia*+remaining clades | Biotin and derivatives (both); Piperidines (pos); Sulfenyl compounds (pos) | Benzofurans (neg); Dithioles (neg); Furanoid lignans (both); Metalloheterocyclic compounds (pos); Propargyl-type 1,3-dipolar organic compounds (pos) |
| *Camarea* | Benzothiazoles (both); Depsides and depsidones (neg); Diazanaphthalenes (pos); Halohydrins (neg); Keto acids and derivatives (neg); Propargyl-type 1,3-dipolar organic compounds (pos) | Benzopyrans (both); Organic phosphonic acids and derivatives (pos) |
| *Camarea*+*Gaudichaudia* clade | Phenylpropanoic acids (neg) | Organochlorides (both); Oxanes (both) |
| *Gaudichaudia* | (3'→5')-dinucleotides and analogues (neg); Benzodioxoles (both); Isoindoles and derivatives (both); Organofluorides (pos); Pyrimidine nucleosides (both); Quinolizines (pos); Thiocarbonyl compounds (pos) | – |

^†^**Classes retrieved as present in all Malpighiaceae clades:** Alkyl halides (both); Allyl-type 1,3-dipolar organic compounds (both); Aryl halides (both); Azacyclic compounds (both); Azoles (both); Benzene and substituted derivatives (both); Boronic acid derivatives (both); Carboxylic acids and derivatives (both); Cinnamic acids and derivatives (both); Diazinanes (pos); Diazines (both); Fatty Acyls (both); Flavonoids (both); Glycerolipids (both); Heteroaromatic compounds (both); Imidazopyrimidines (both); Macrolides and analogues (pos); Organic metal salts (pos); Organic sulfonic acids and derivatives (both); Organonitrogen compounds (both); Organooxygen compounds (both); Phenol ethers (both); Phenols (both); Prenol lipids (both); Purine nucleosides (both); Purine nucleotides (both); Pyridines and derivatives (both); Sphingolipids (both); Steroids and steroid derivatives (both); Sulfonyls (pos); Thioethers (both).

**^§^**Synapomorphy.
